# Supplementary material for: A CTP-dependent gating mechanism enables ParB spreading on DNA
Source: eLife. 2021 Aug 16;10:e69676. doi: 10.7554/eLife.69676 (PMC8367383; doi:10.7554/eLife.69676)
Supplement: Figure 5—source data 1. [file elife-69676-fig5-data1.zip › Figure5/PanelB/Annotation.pdf]

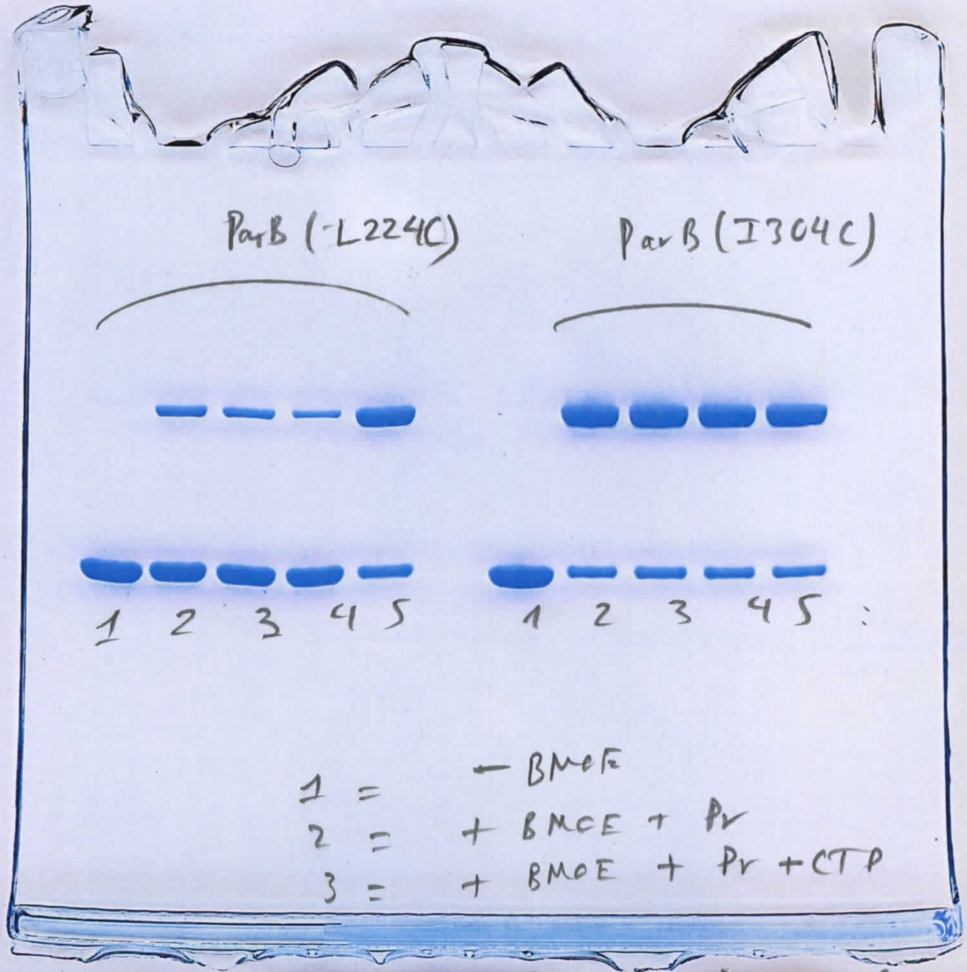

Lane # same as appeared in figure.

4 = + BMOE + Pr + parS
5 = + BMOE + Pr + parS + CTP

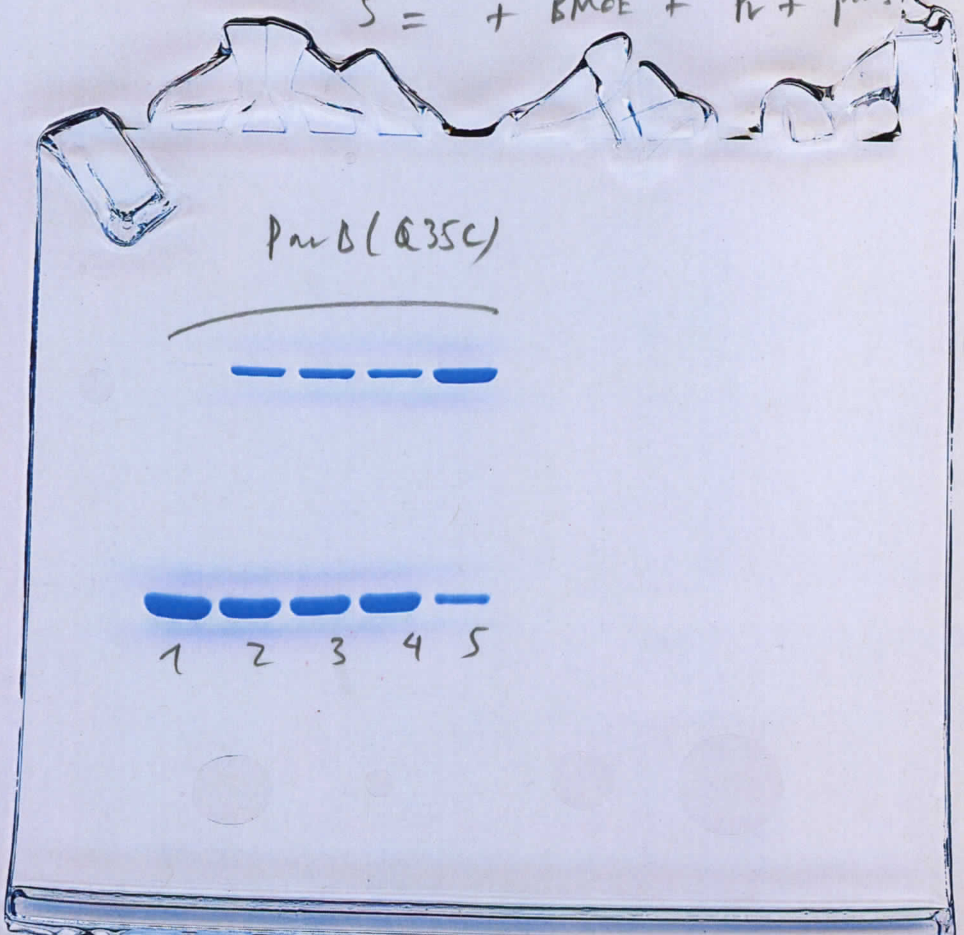

5B
