# Supplementary material for: A CTP-dependent gating mechanism enables ParB spreading on DNA
Source: eLife. 2021 Aug 16;10:e69676. doi: 10.7554/eLife.69676 (PMC8367383; doi:10.7554/eLife.69676)
Supplement: Figure 5—source data 1. [file elife-69676-fig5-data1.zip › Figure5/PanelC/Annotation.pdf]

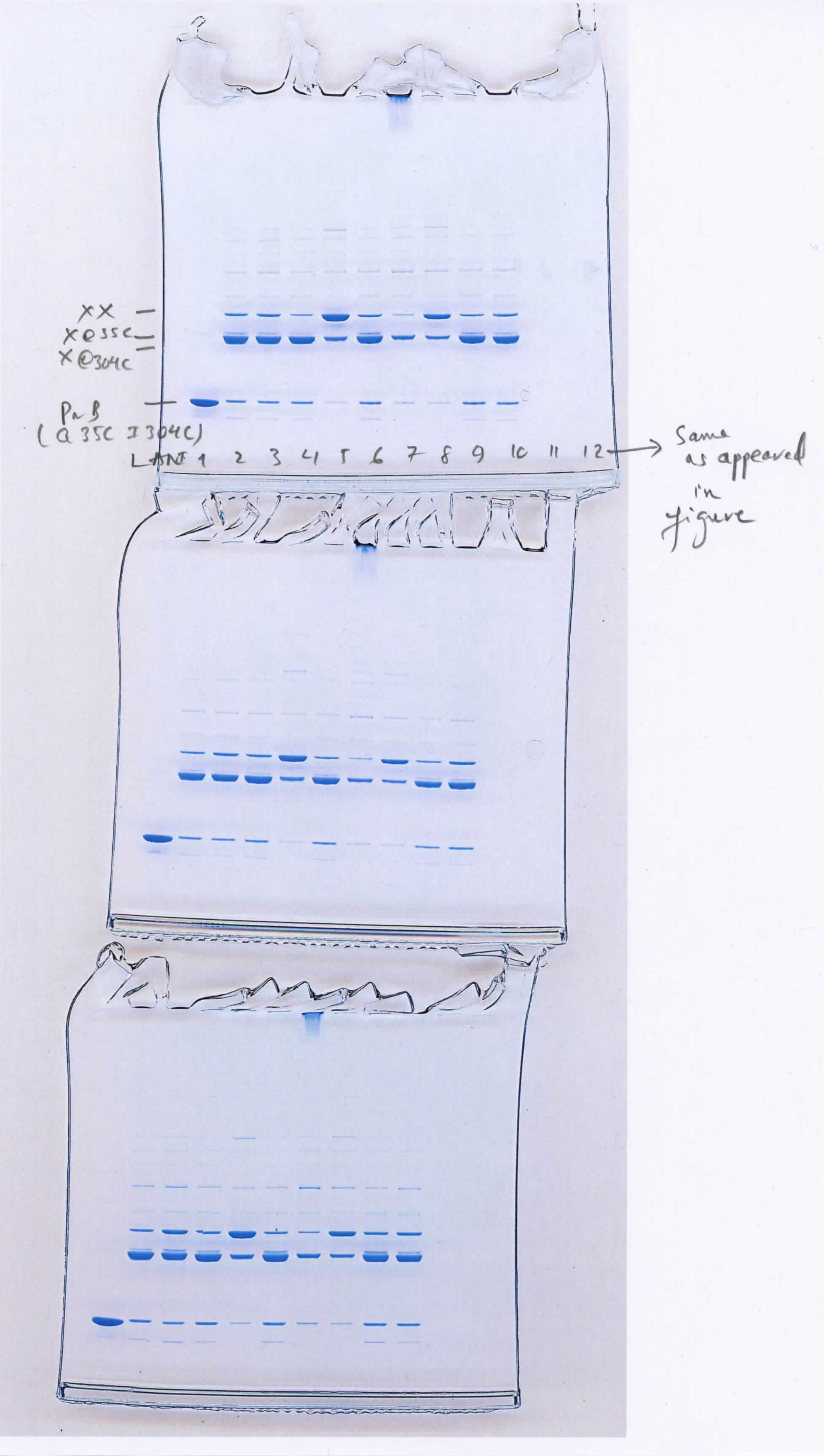
XX
X@35C
X@304C
PnB
(Q35C I304C)
LANE 1 2 3 4 5 6 7 8 9 10 11 12
Same as appeared in figure

5C

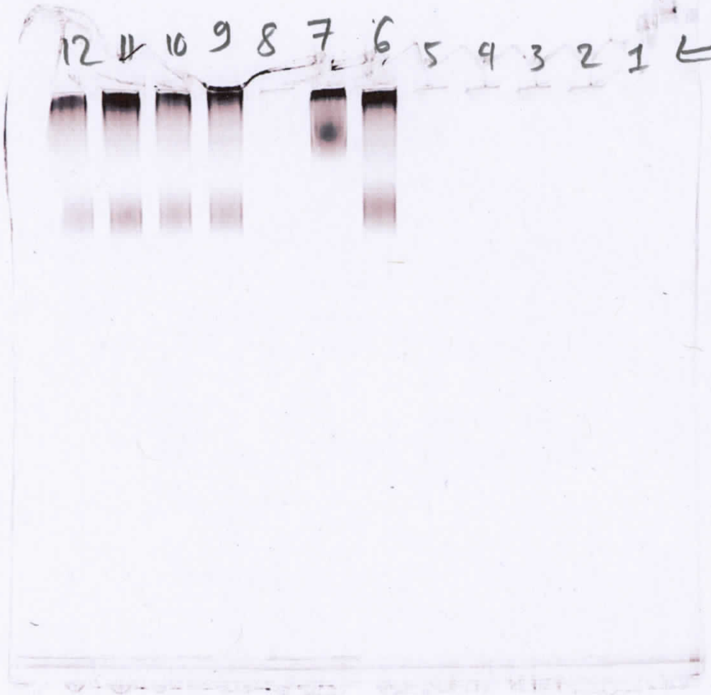

← Lane #: same as appeared in figure

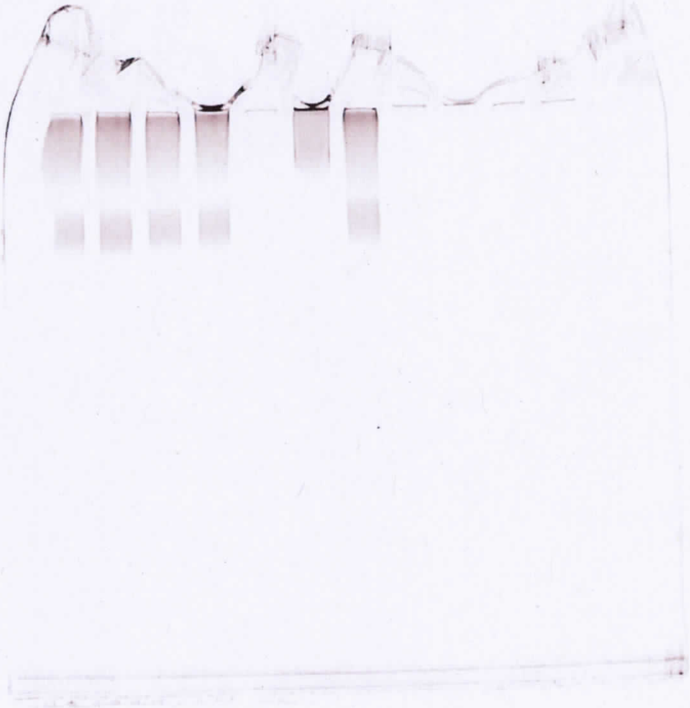

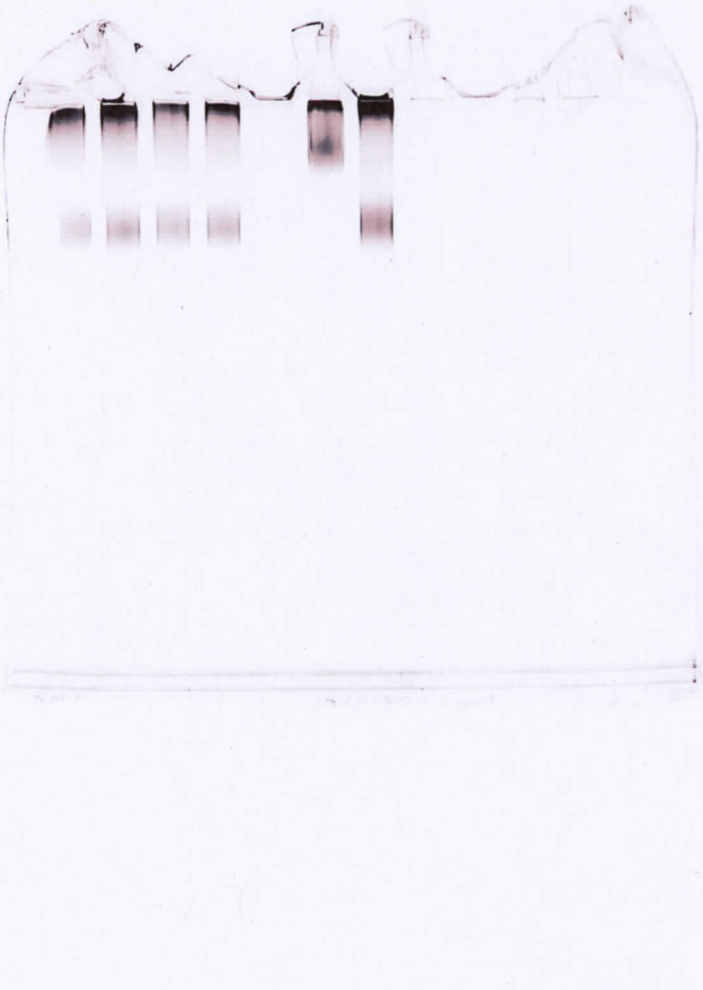

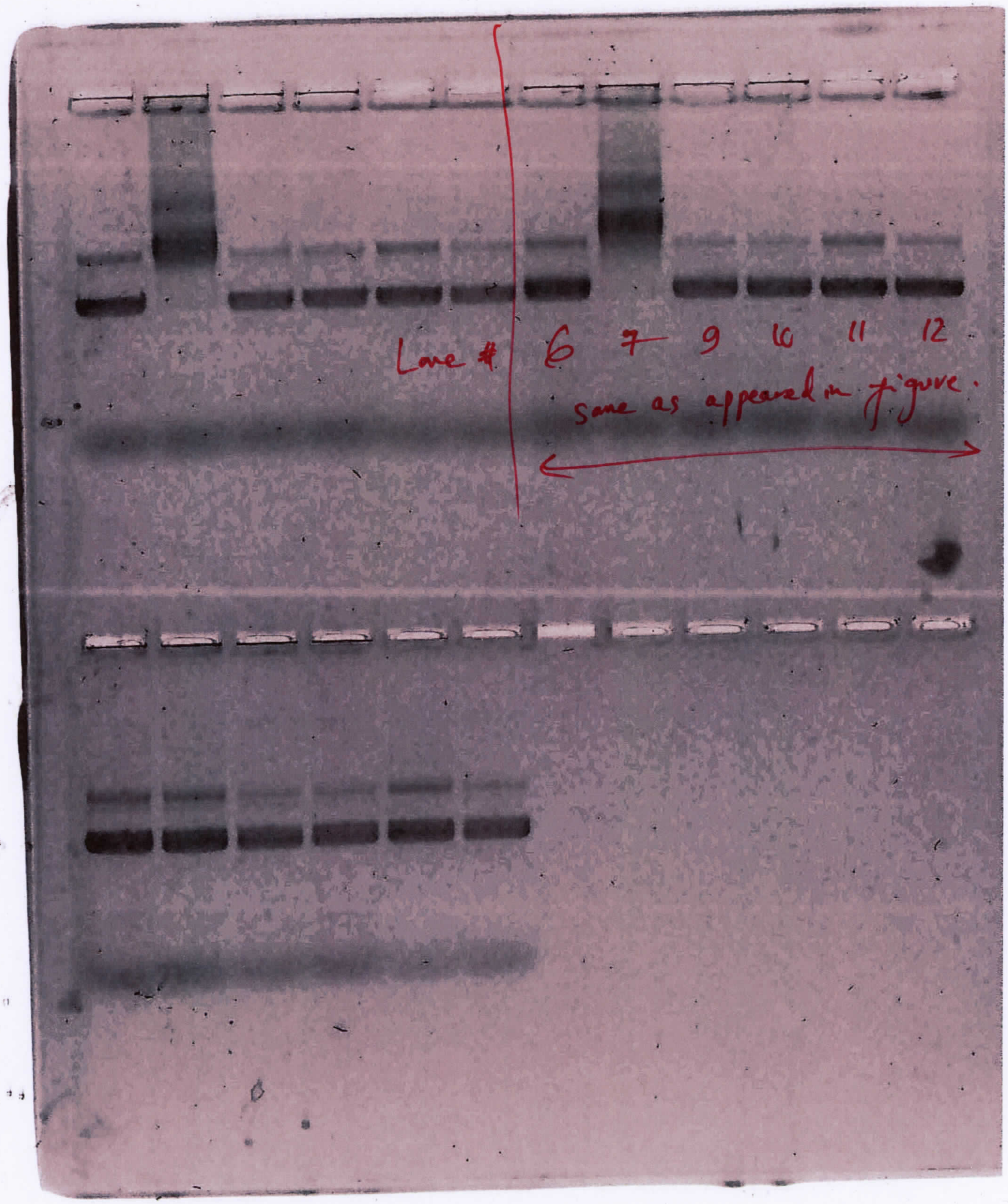
Lane #
6 7 9 10 11 12
same as appeared in figure.
