# Supplementary figures and images for: A CTP-dependent gating mechanism enables ParB spreading on DNA
Source: eLife. 2021 Aug 16;10:e69676. doi: 10.7554/eLife.69676 (PMC8367383; doi:10.7554/eLife.69676)

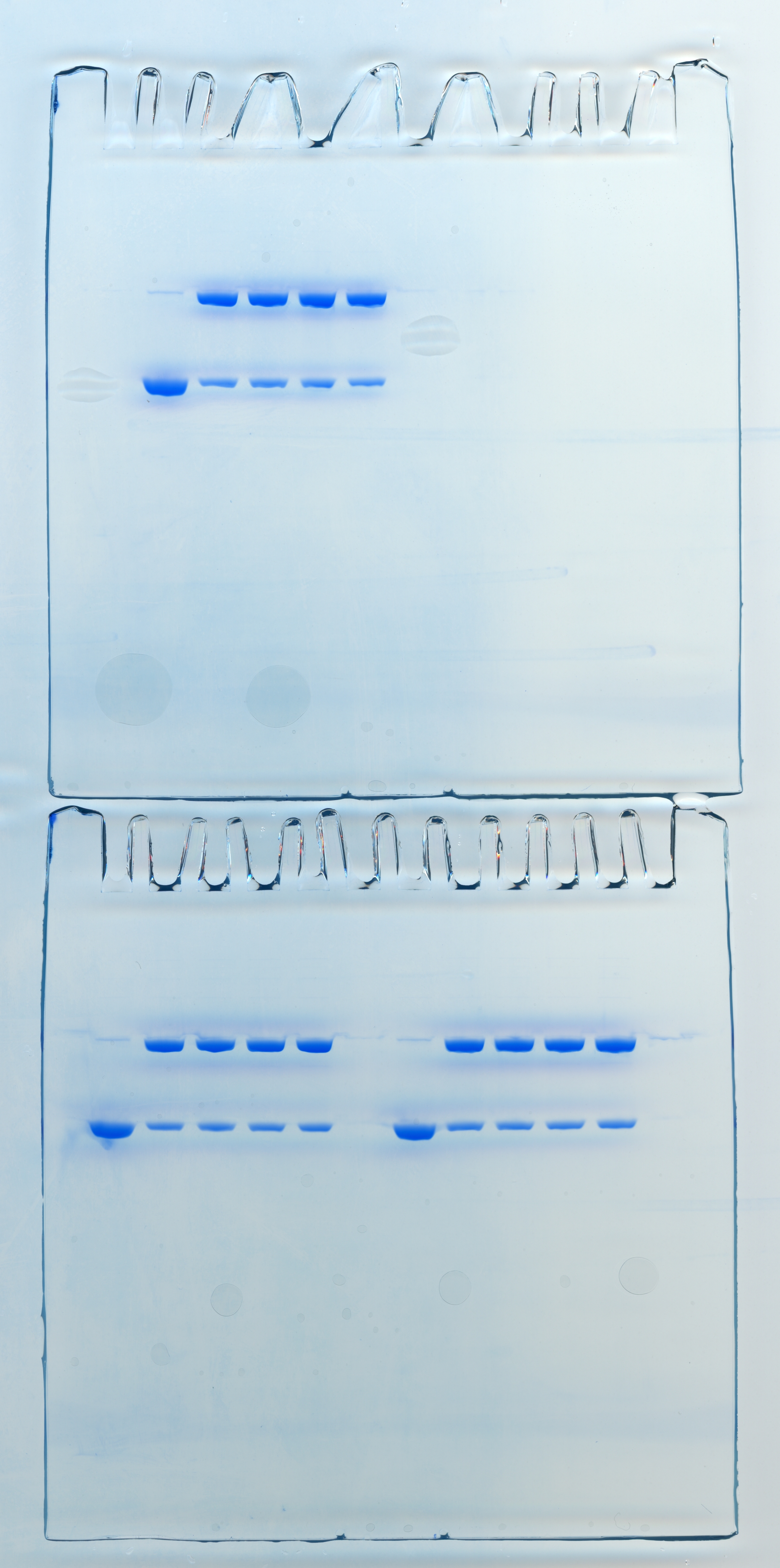

Supplement: Figure 5—source data 1. [file elife-69676-fig5-data1.zip › Figure5/PanelB/other_replicates/I304C_Xlink_other_replicates.jpg]

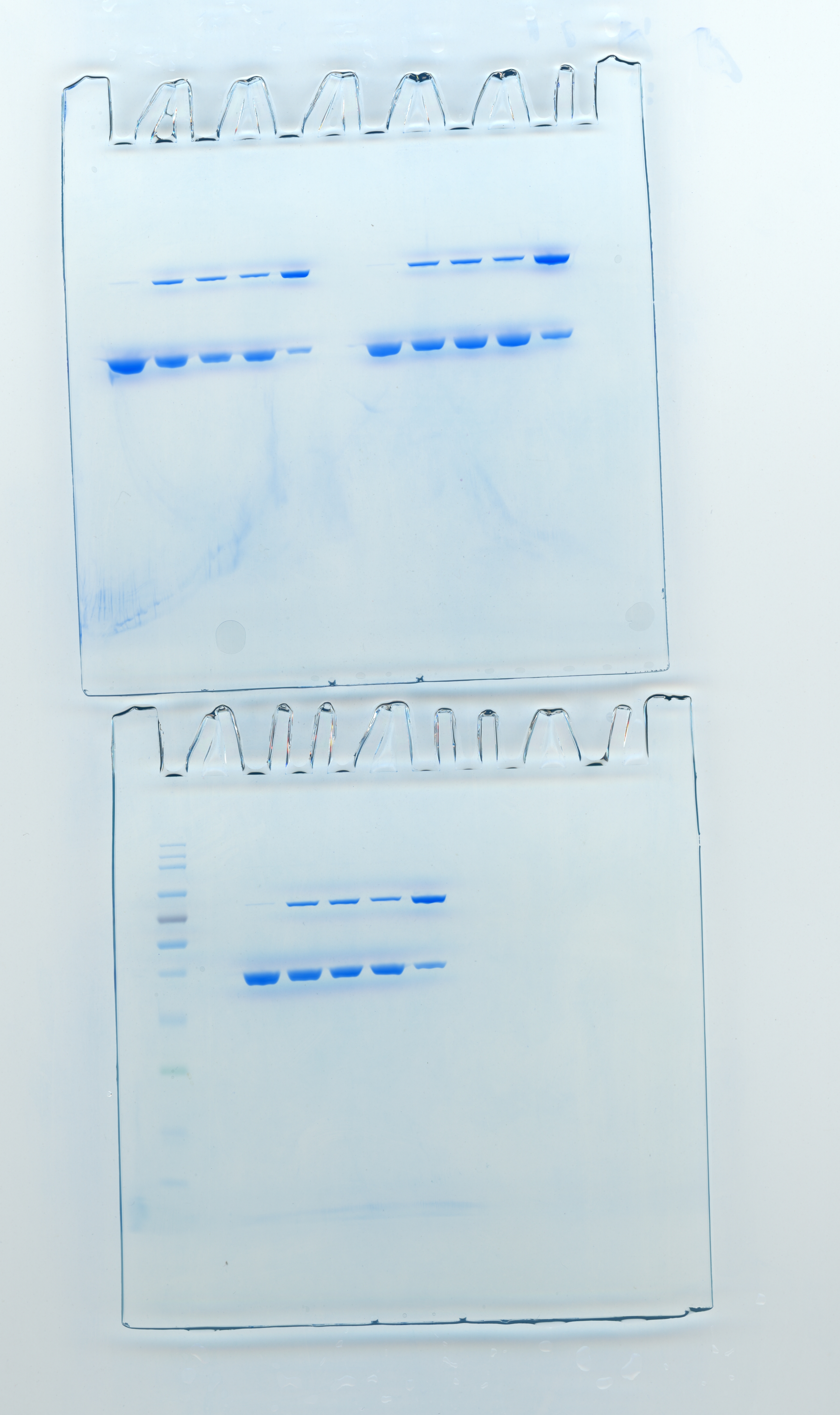

Supplement: Figure 5—source data 1. [file elife-69676-fig5-data1.zip › Figure5/PanelB/other_replicates/L224C_Xlink_other_replicates.jpg]

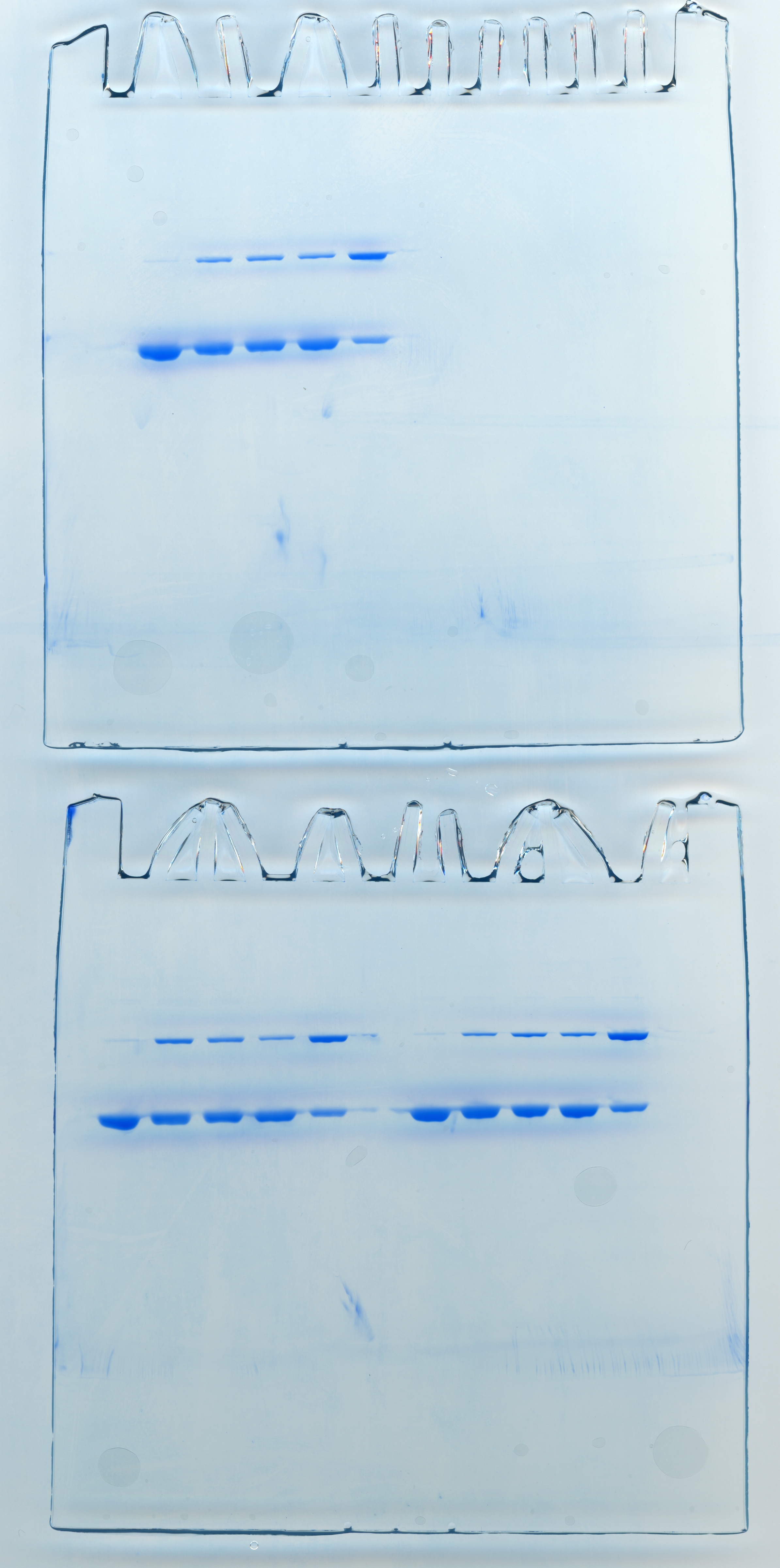

Supplement: Figure 5—source data 1. [file elife-69676-fig5-data1.zip › Figure5/PanelB/other_replicates/Q35C_Xlink_other_replicates.jpg]

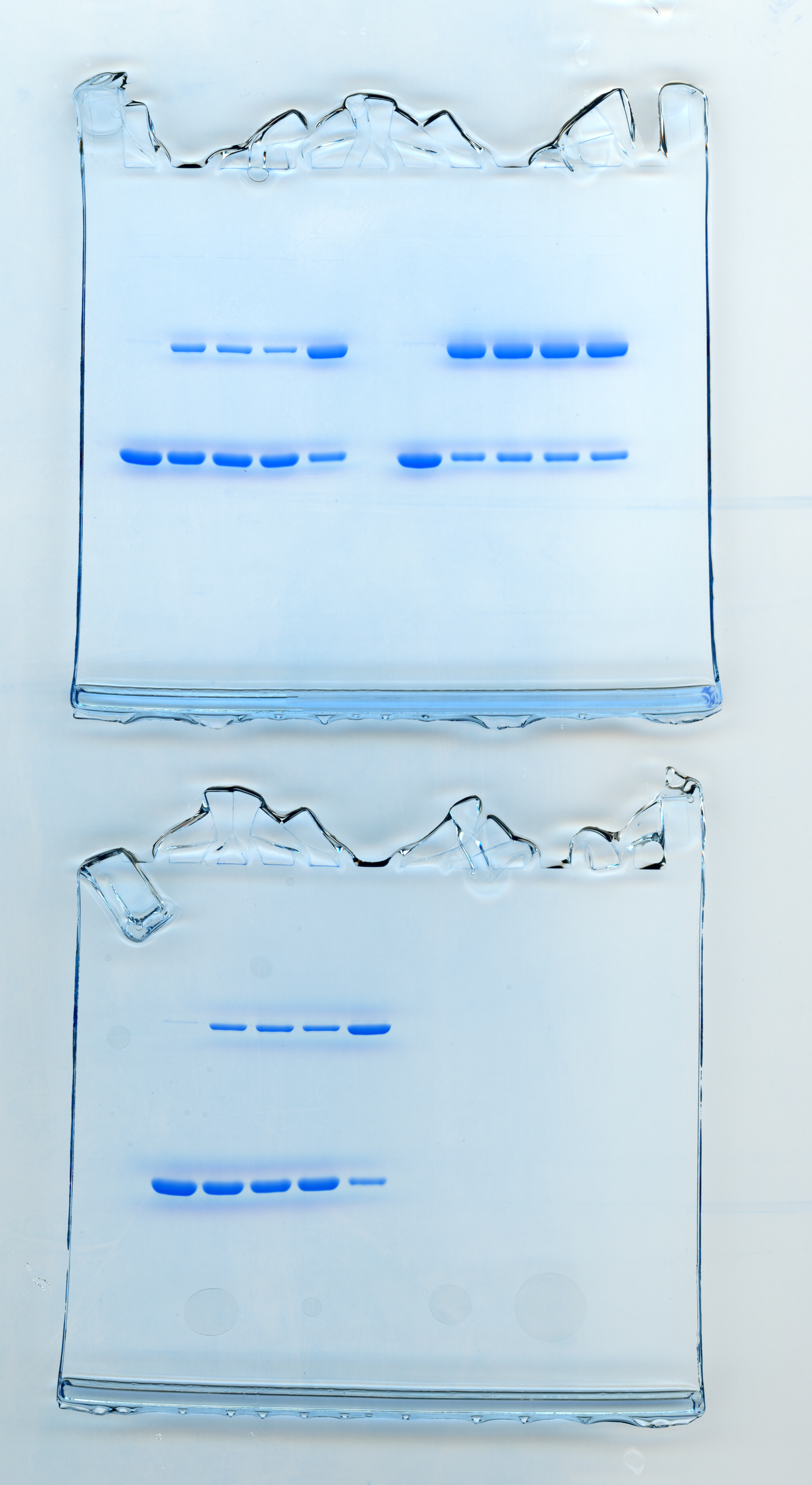

Supplement: Figure 5—source data 1. [file elife-69676-fig5-data1.zip › Figure5/PanelB/Xlink_L224C_I304C_Q35C.tif]

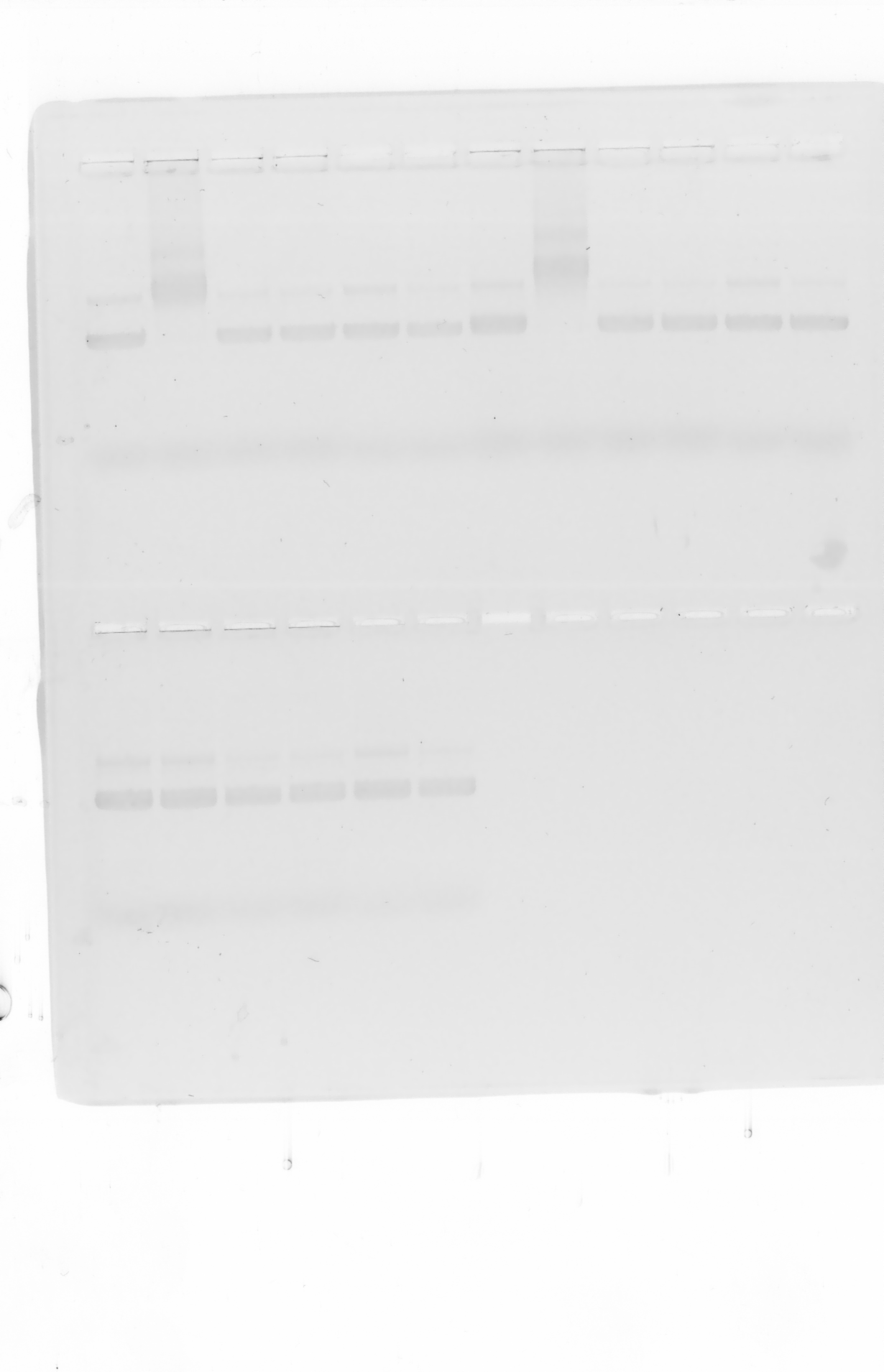

Supplement: Figure 5—source data 1. [file elife-69676-fig5-data1.zip › Figure5/PanelC/Double_Xlink_agarose_topright_gel.tif]

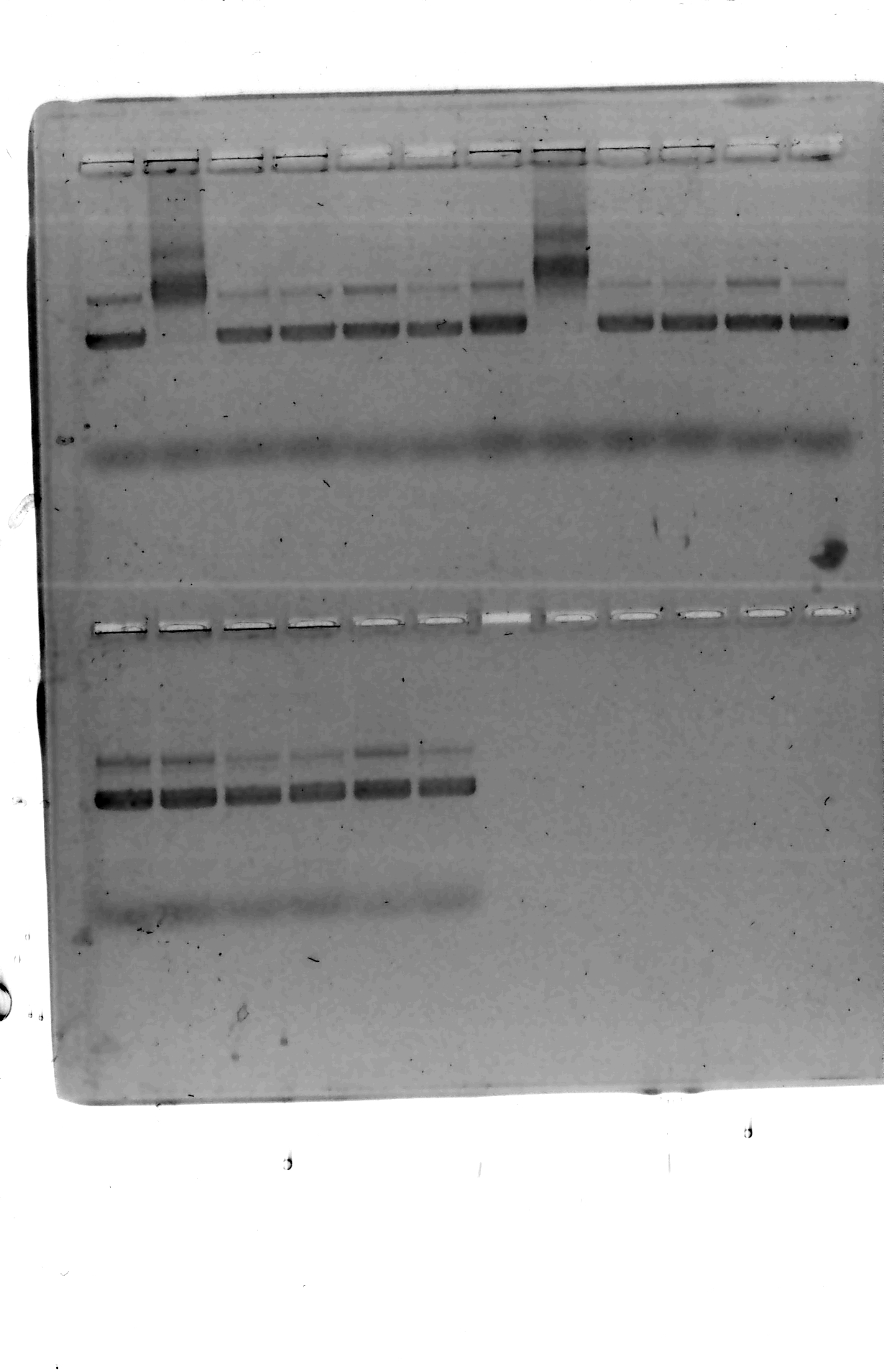

Supplement: Figure 5—source data 1. [file elife-69676-fig5-data1.zip › Figure5/PanelC/DoubleXlink_agarose_topright_gel.jpg]

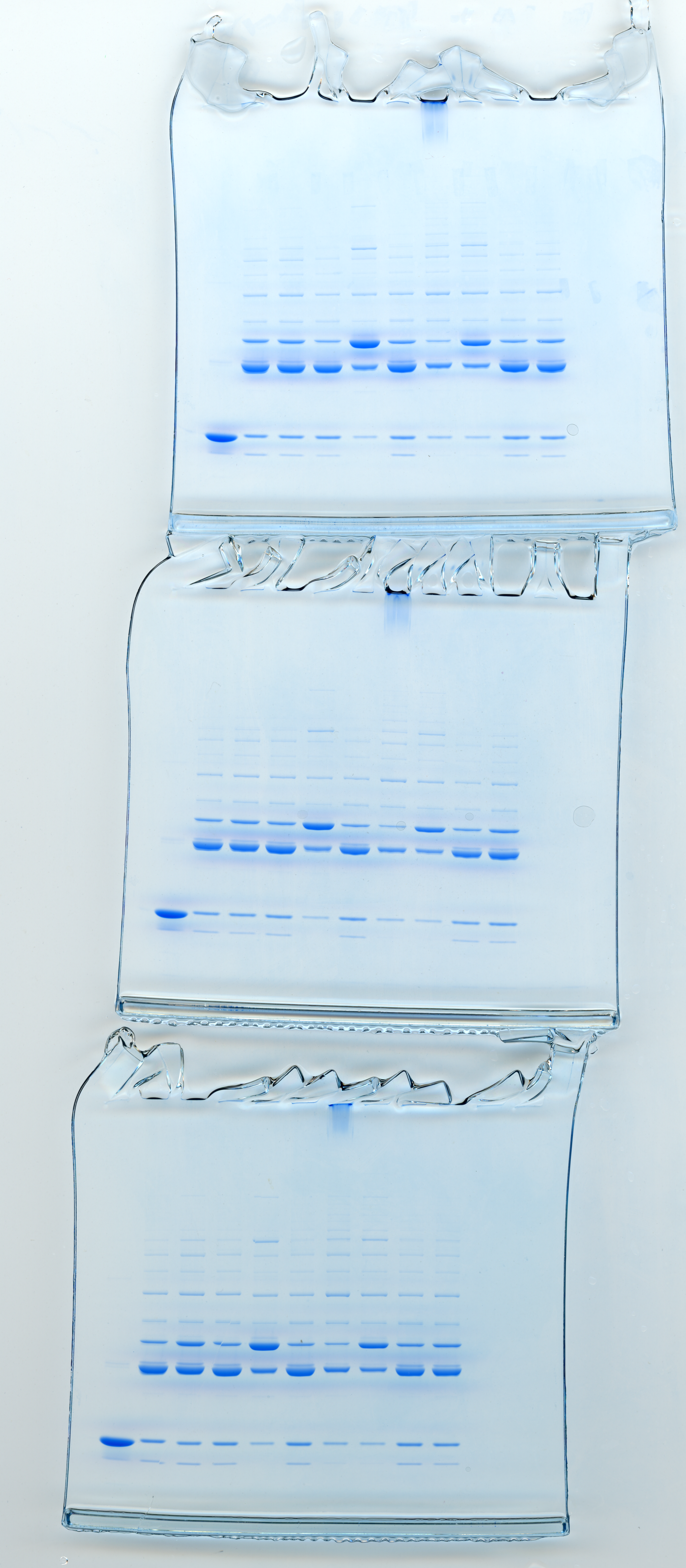

Supplement: Figure 5—source data 1. [file elife-69676-fig5-data1.zip › Figure5/PanelC/DoubleXlink_N_C_lane1-12_Coomassie.tif]

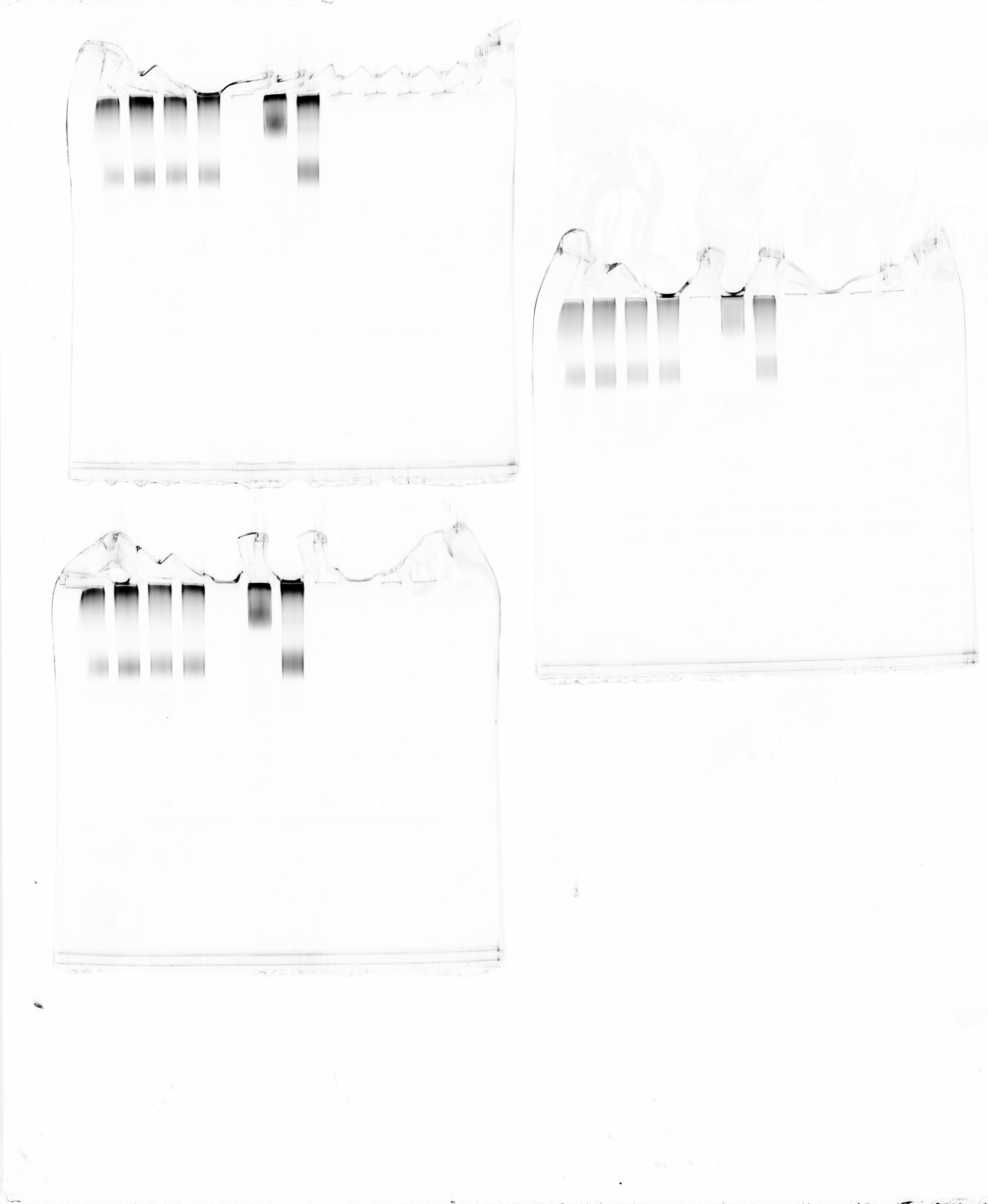

Supplement: Figure 5—source data 1. [file elife-69676-fig5-data1.zip › Figure5/PanelC/DoubleXlink_N_C_sybrgreen.tif]

XX —  
 X 304C —  
 X 224C —  
 PurB —  
 (L224C I304C)  
 LANE

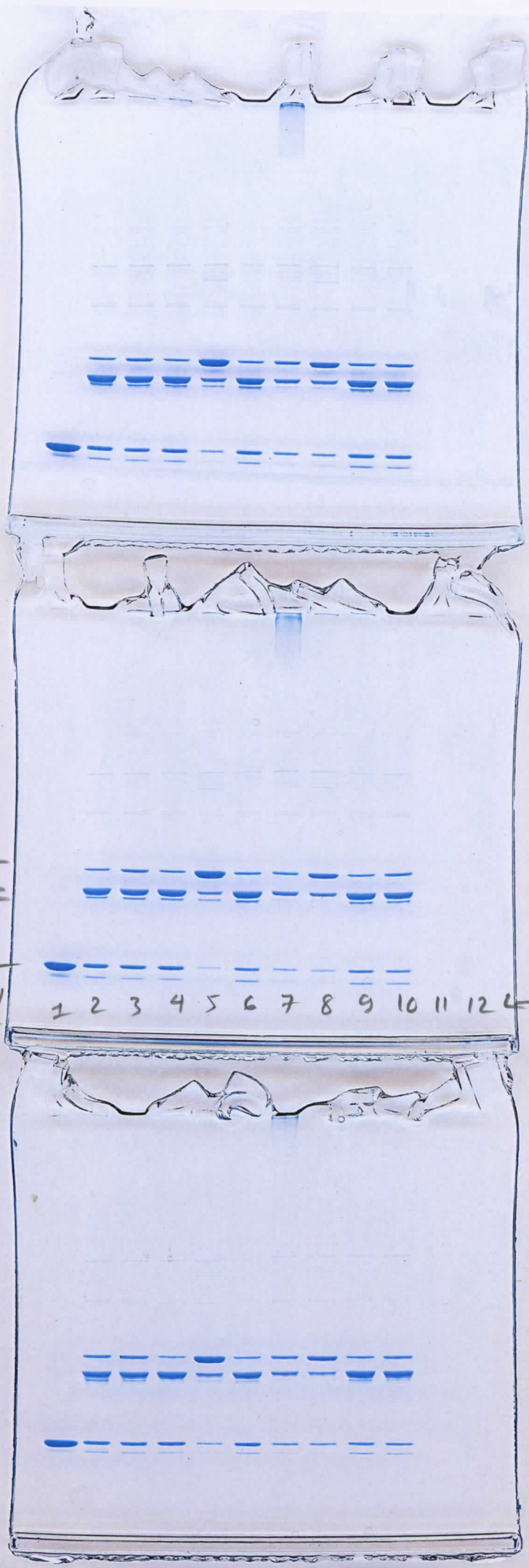

lanes  
as appeared  
in figure.

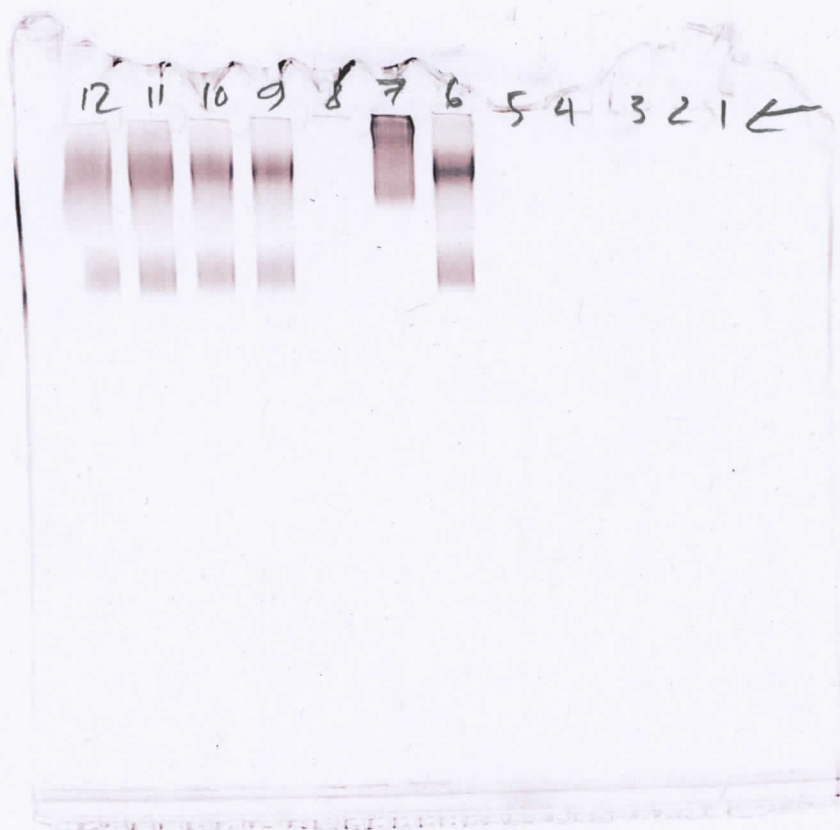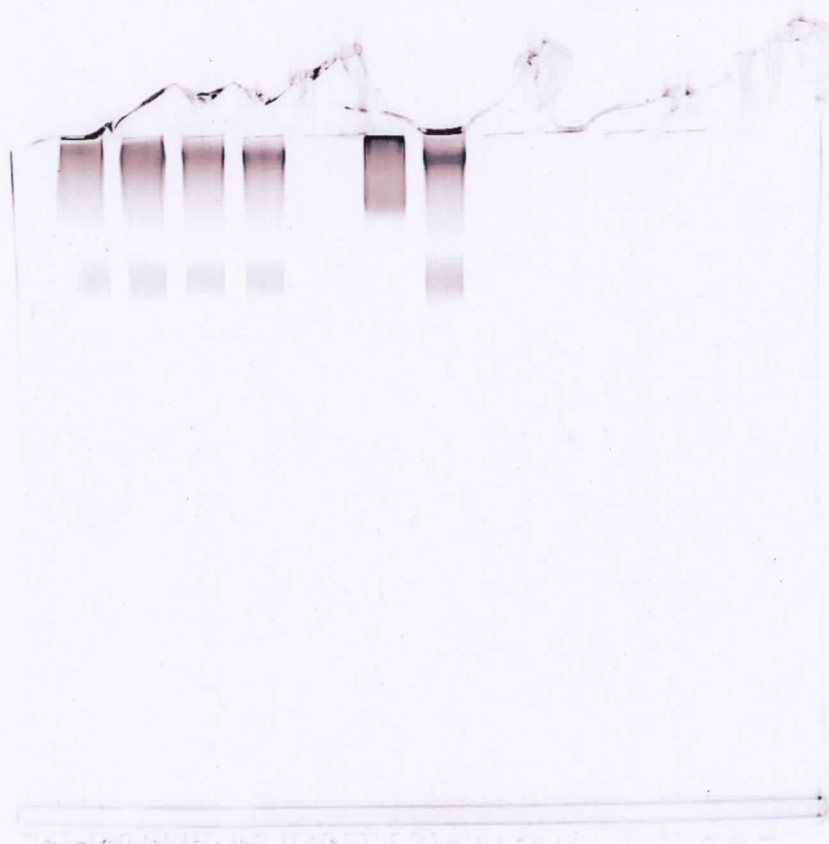

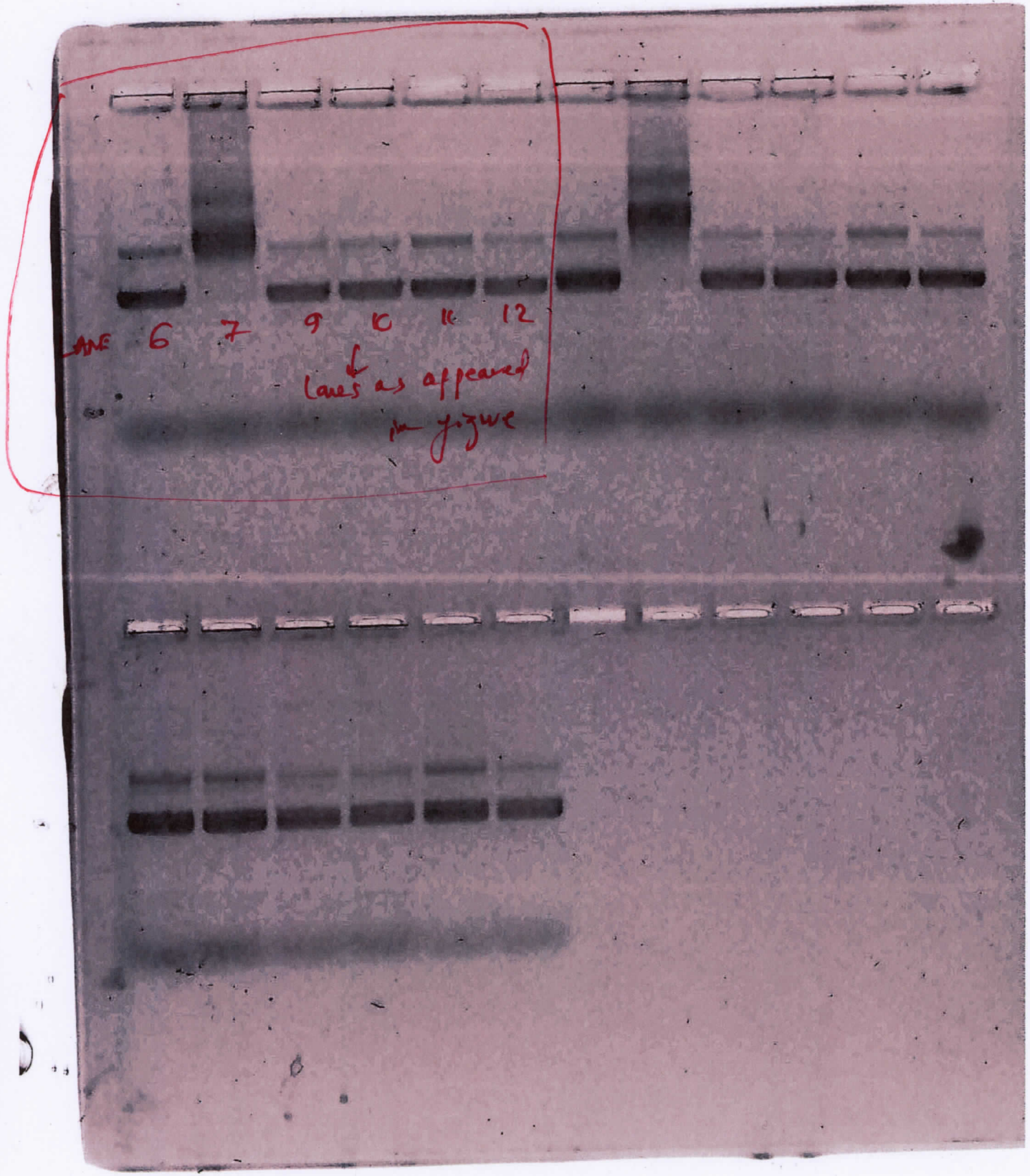

LANE 6 7 9 10 11 12  
↓  
lanes as appeared  
in figure

Supplement: Figure 5—source data 1. [file elife-69676-fig5-data1.zip › Figure5/PanelD/Annotation.pdf]

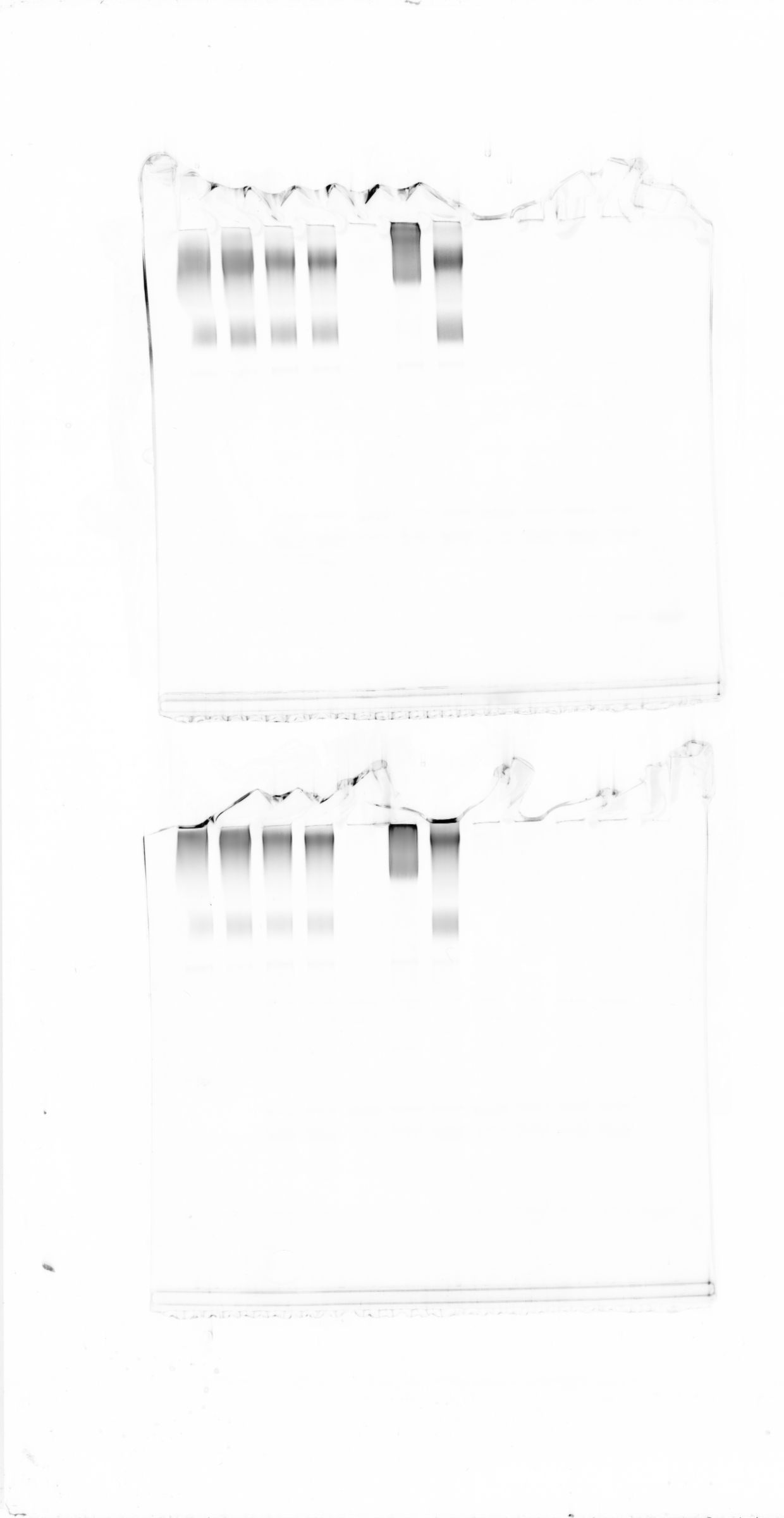

Supplement: Figure 5—source data 1. [file elife-69676-fig5-data1.zip › Figure5/PanelD/DooubleXlink_C_DBD_sybrgreen.tif]

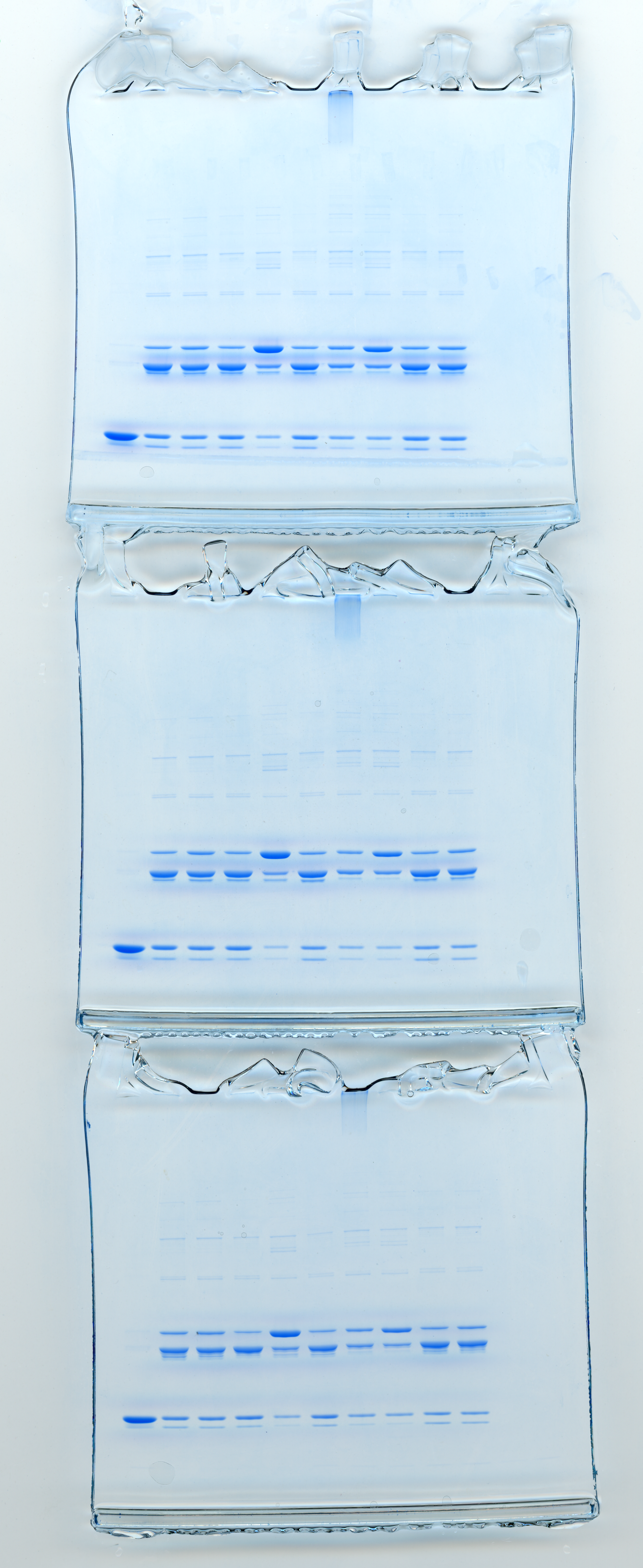

Supplement: Figure 5—source data 1. [file elife-69676-fig5-data1.zip › Figure5/PanelD/DoubleXlink_C_DBD_lane1-12_Coomassie.tif]

Fig 5 - fig suppl 1

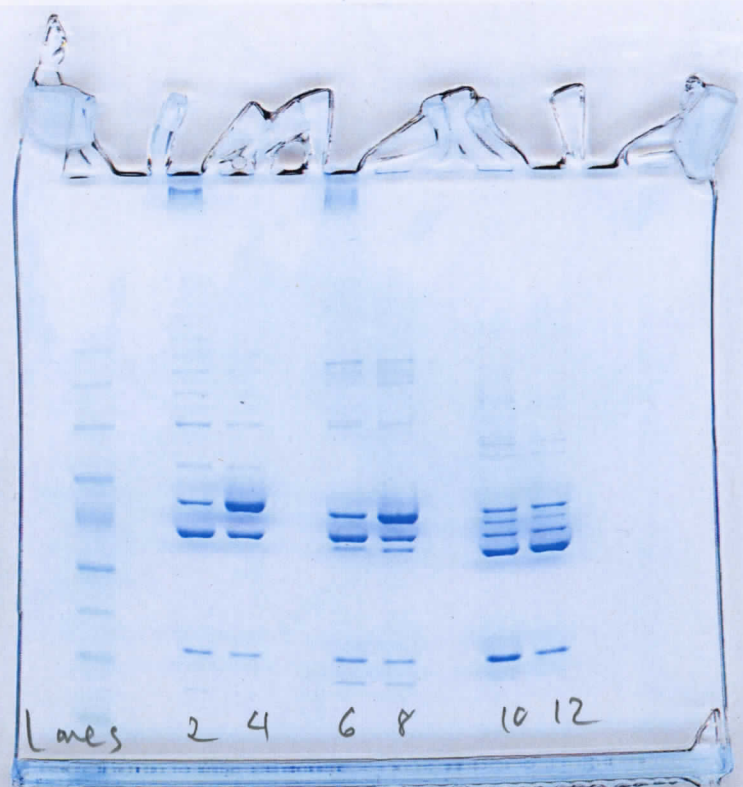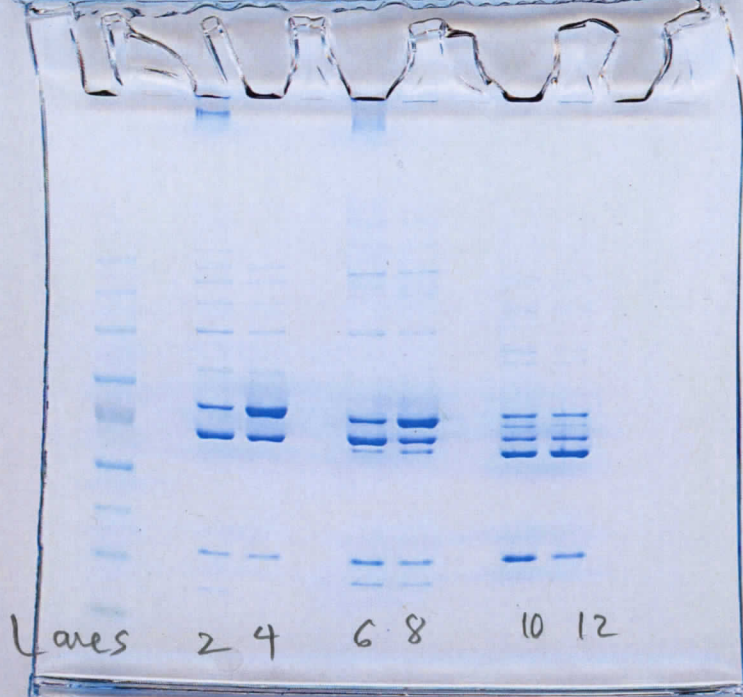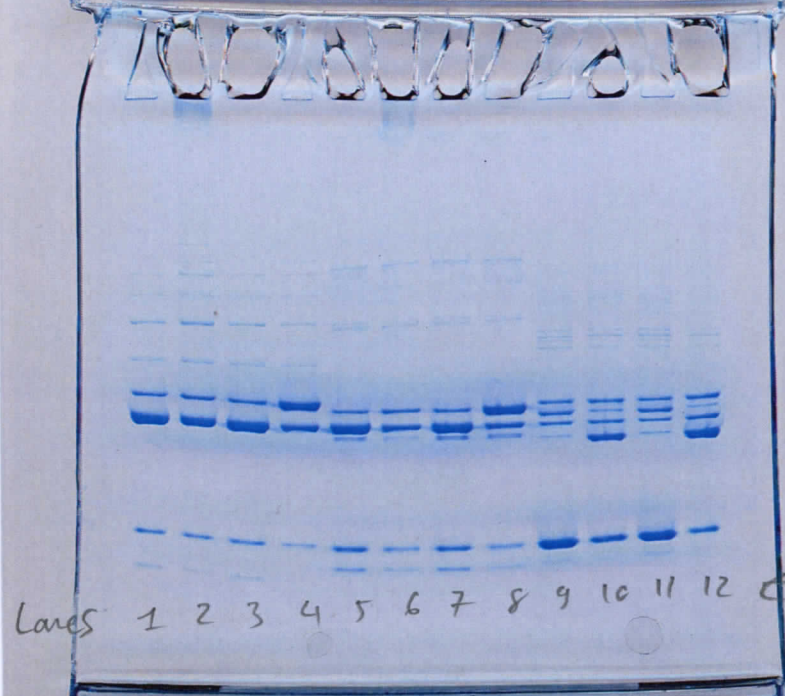

in figure  
as in figure.

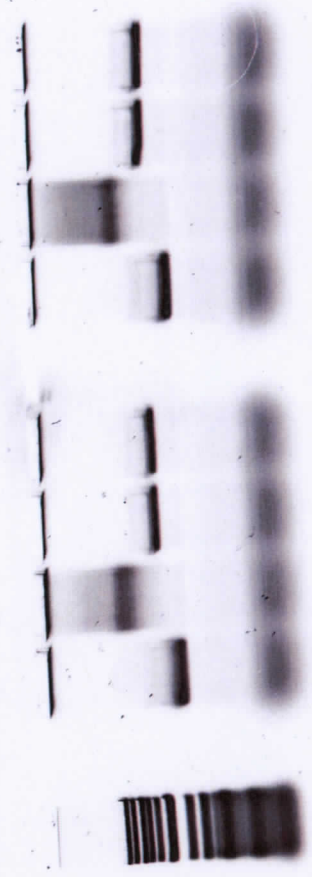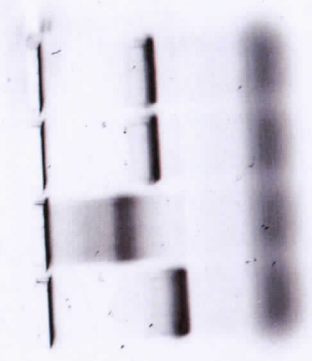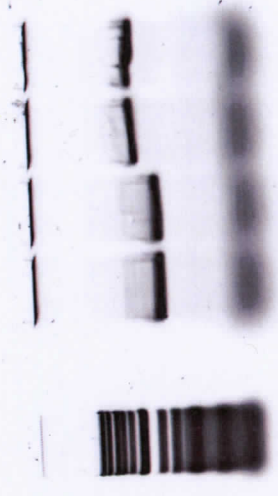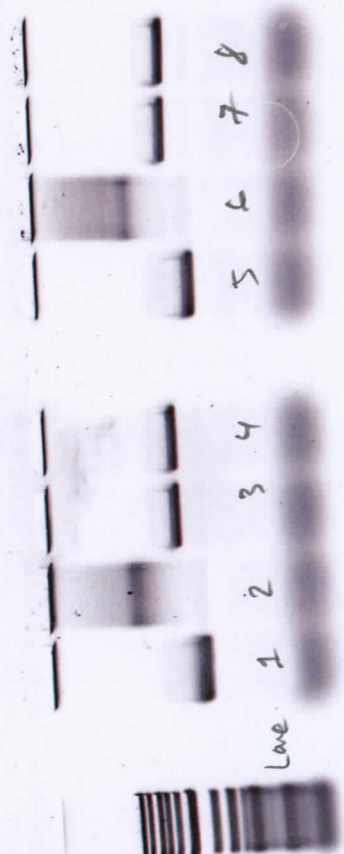

5 6 7 8

3 4

Lane 1 2

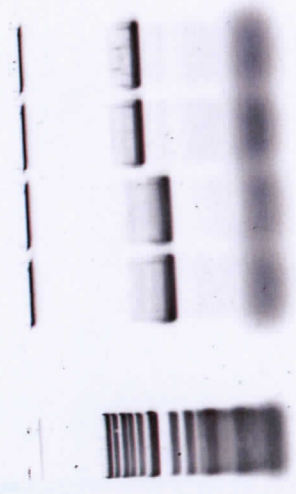

9 10 11 12 ← as in figure

Supplement: Figure 5—figure supplement 1—source data 1. [file elife-69676-fig5-figsupp1-data1.zip › Figure5_figure_supplement1/Annotation.pdf]

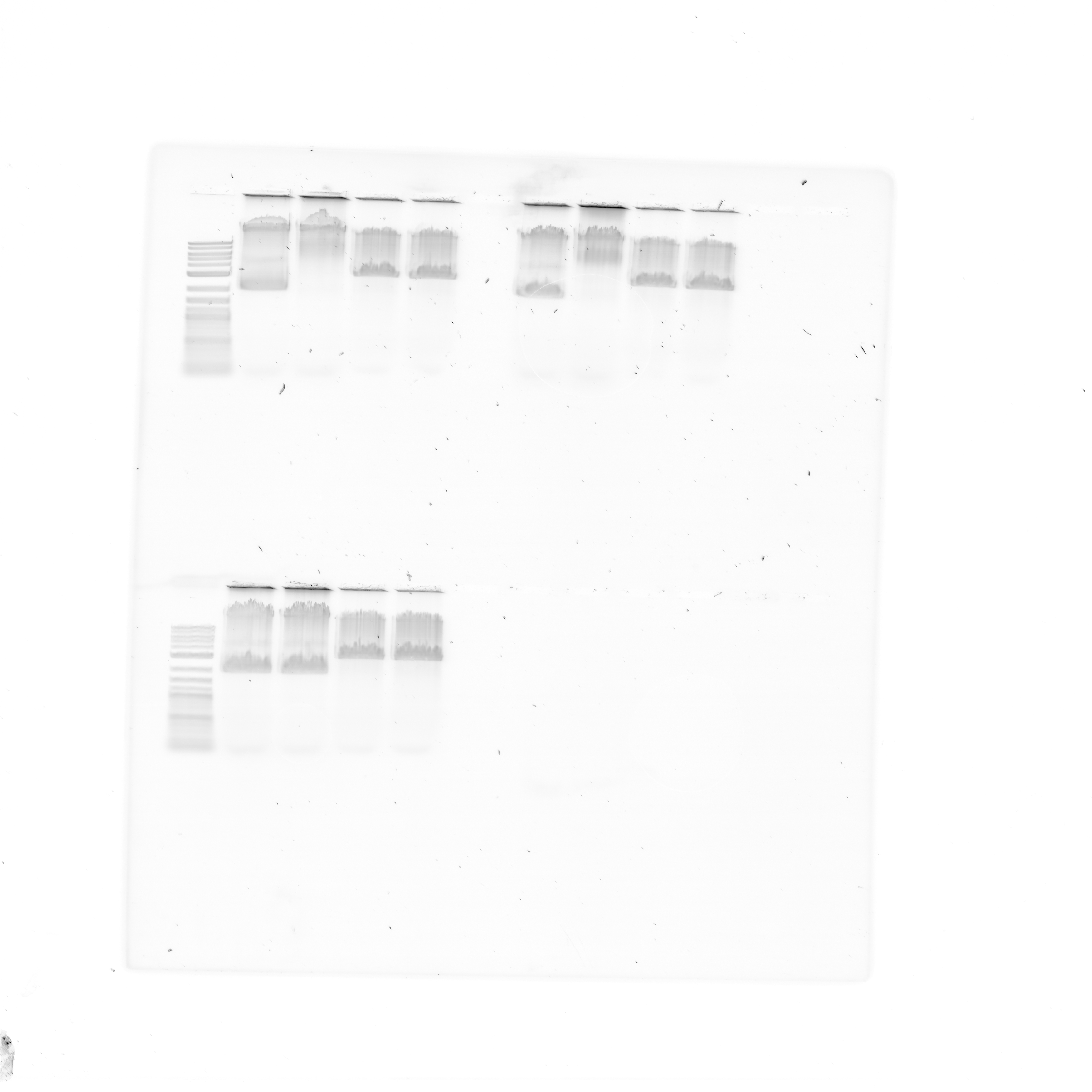

Supplement: Figure 5—figure supplement 1—source data 1. [file elife-69676-fig5-figsupp1-data1.zip › Figure5_figure_supplement1/linearDNA_doubleXlink_agarose_replicate1.tif]

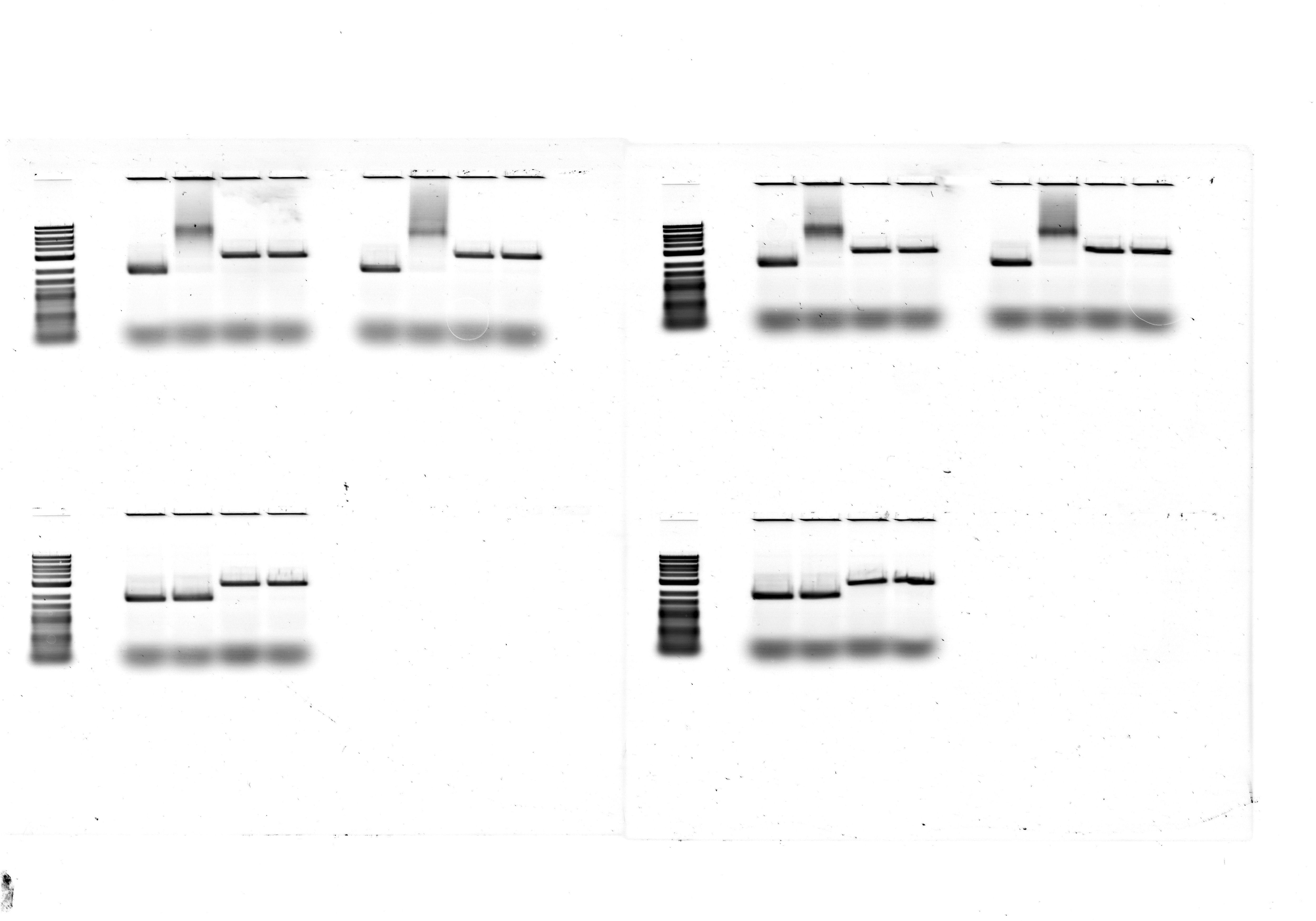

Supplement: Figure 5—figure supplement 1—source data 1. [file elife-69676-fig5-figsupp1-data1.zip › Figure5_figure_supplement1/linearDNA_doubleXlink_agarose_replicates2_3.jpg]

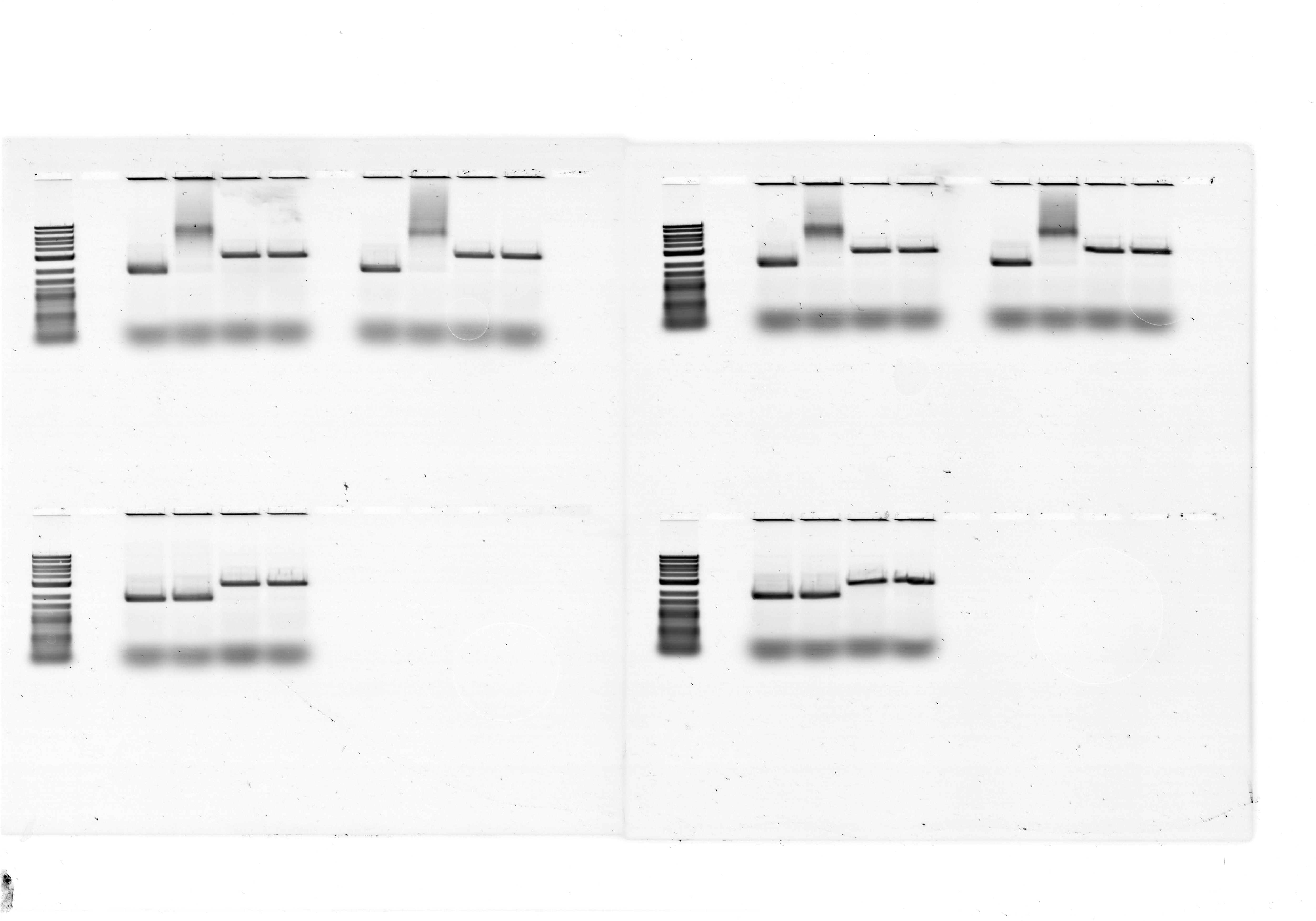

Supplement: Figure 5—figure supplement 1—source data 1. [file elife-69676-fig5-figsupp1-data1.zip › Figure5_figure_supplement1/linearDNA_doubleXlink_agarose_replicates2_3.tif]

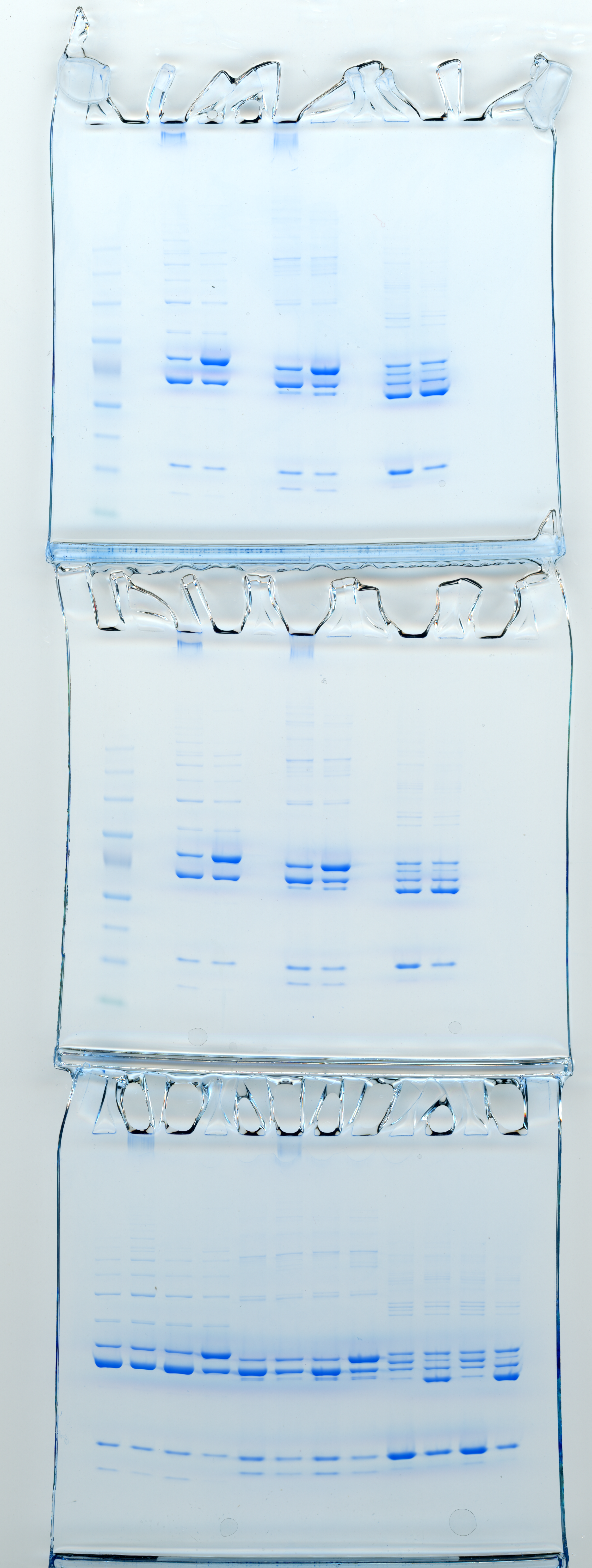

Supplement: Figure 5—figure supplement 1—source data 1. [file elife-69676-fig5-figsupp1-data1.zip › Figure5_figure_supplement1/linearDNA_doubleXlink_and_replicates_SDSPAGE.tif]

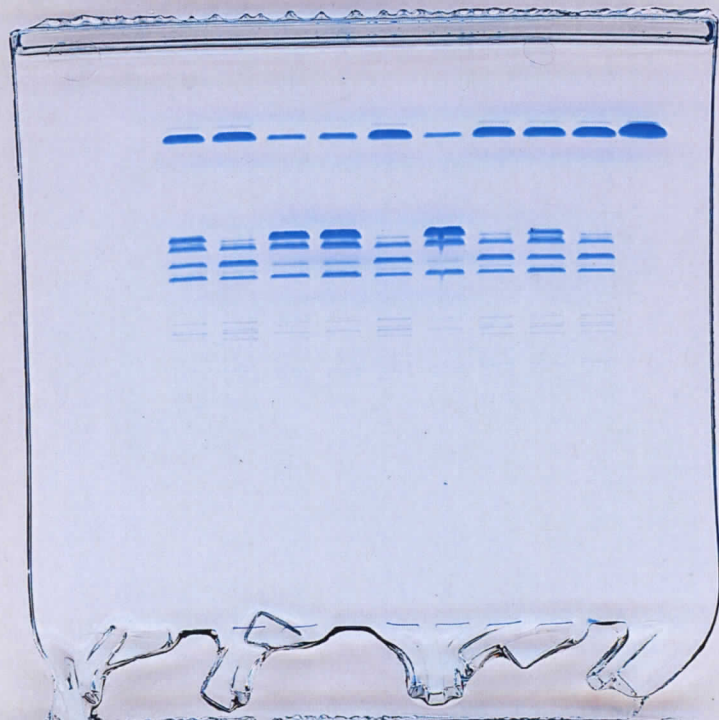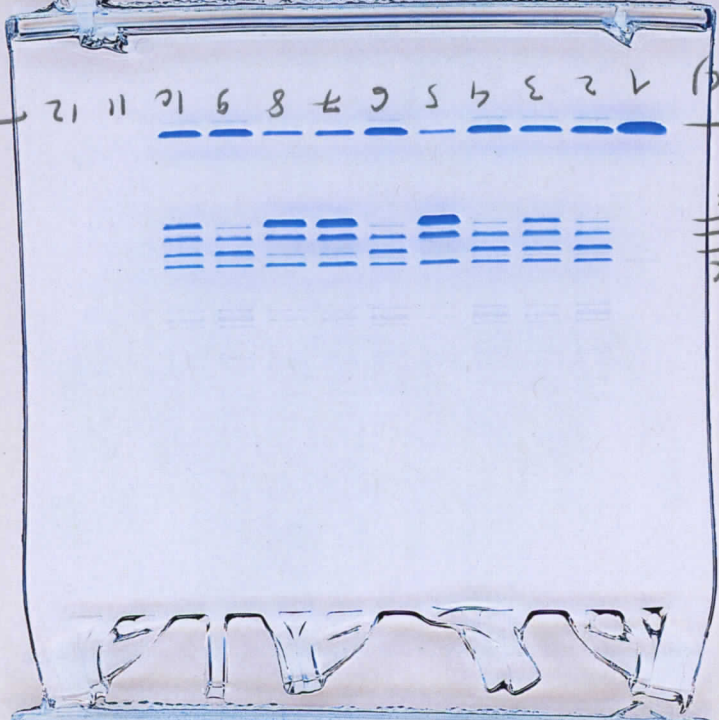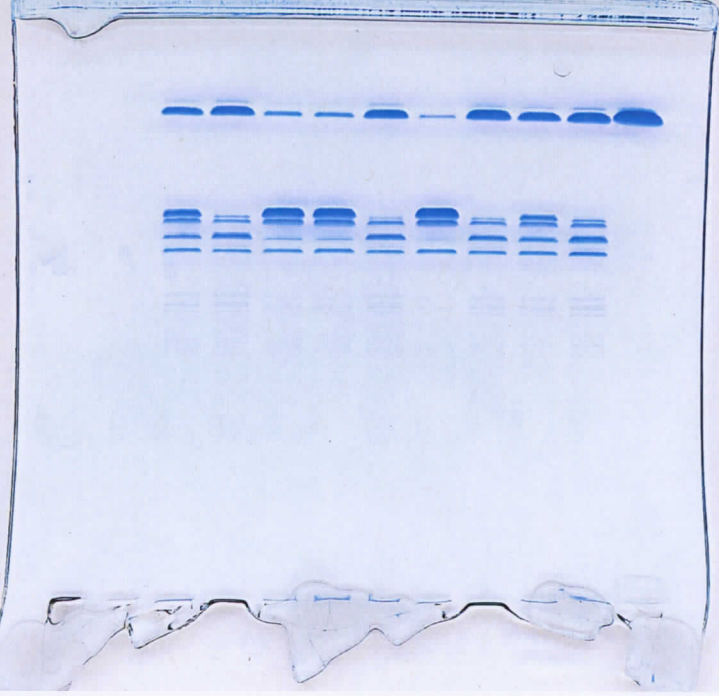

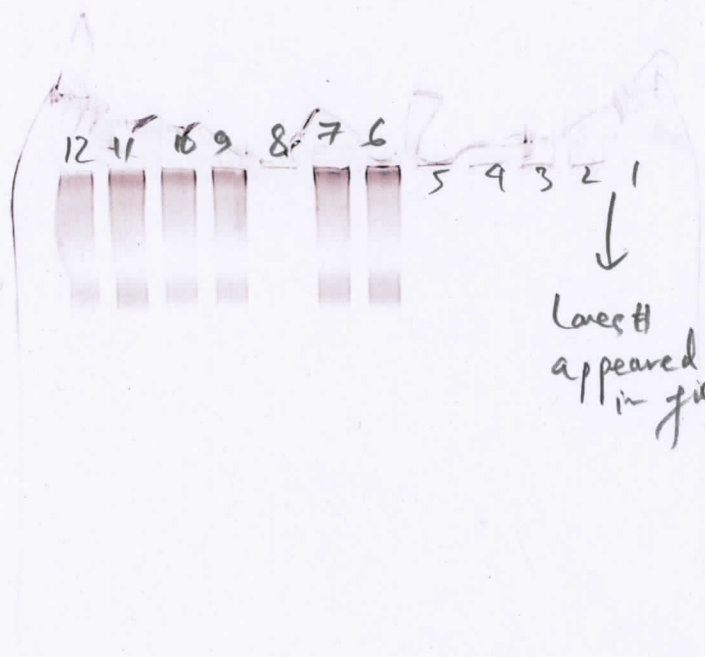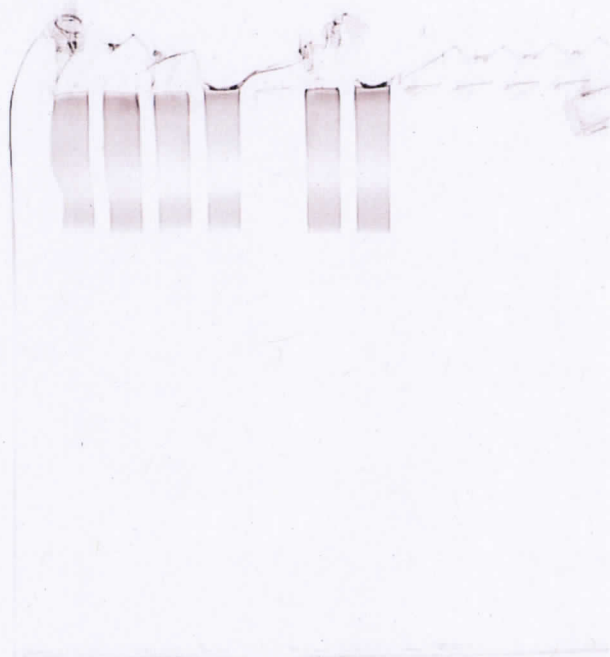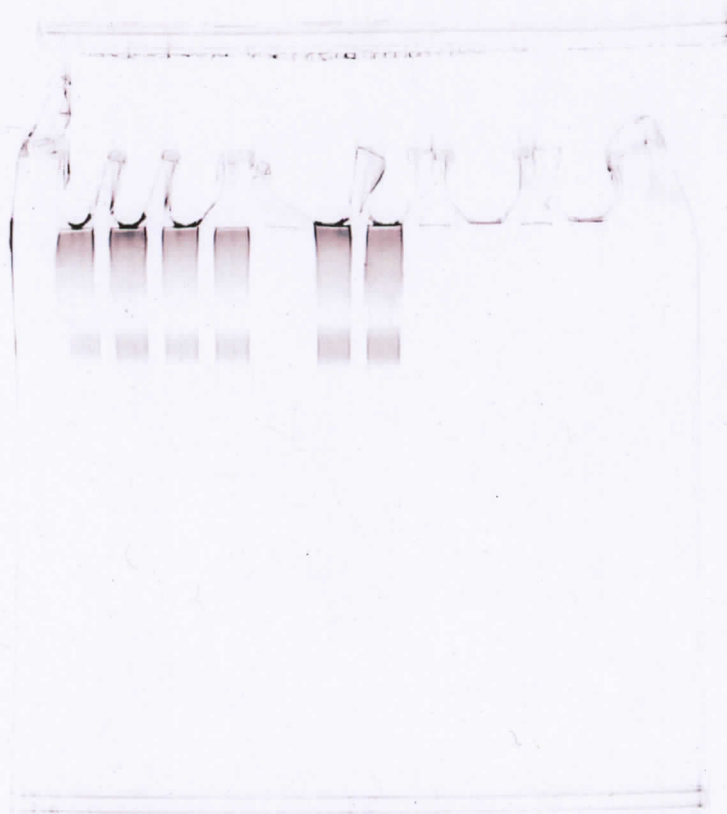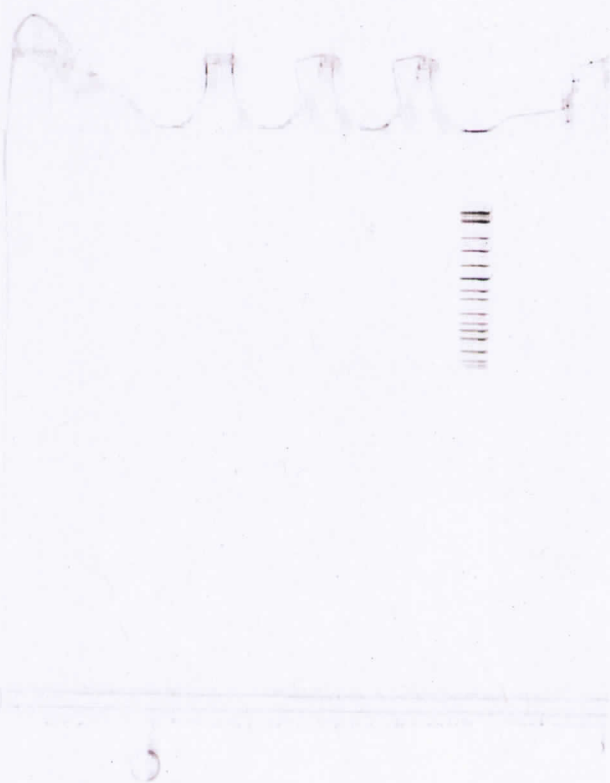

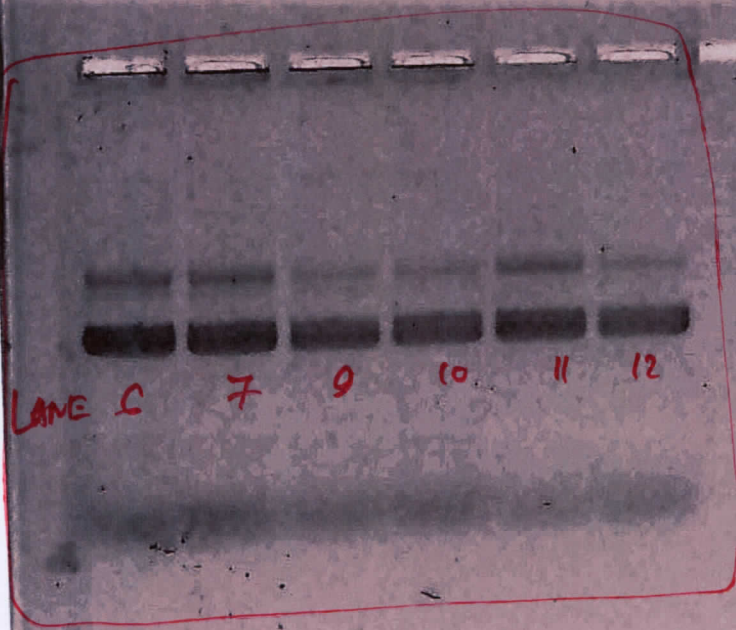

Supplement: Figure 5—figure supplement 2—source data 1. [file elife-69676-fig5-figsupp2-data1.zip › Figure5_figure_supplement2/Annotation.pdf]

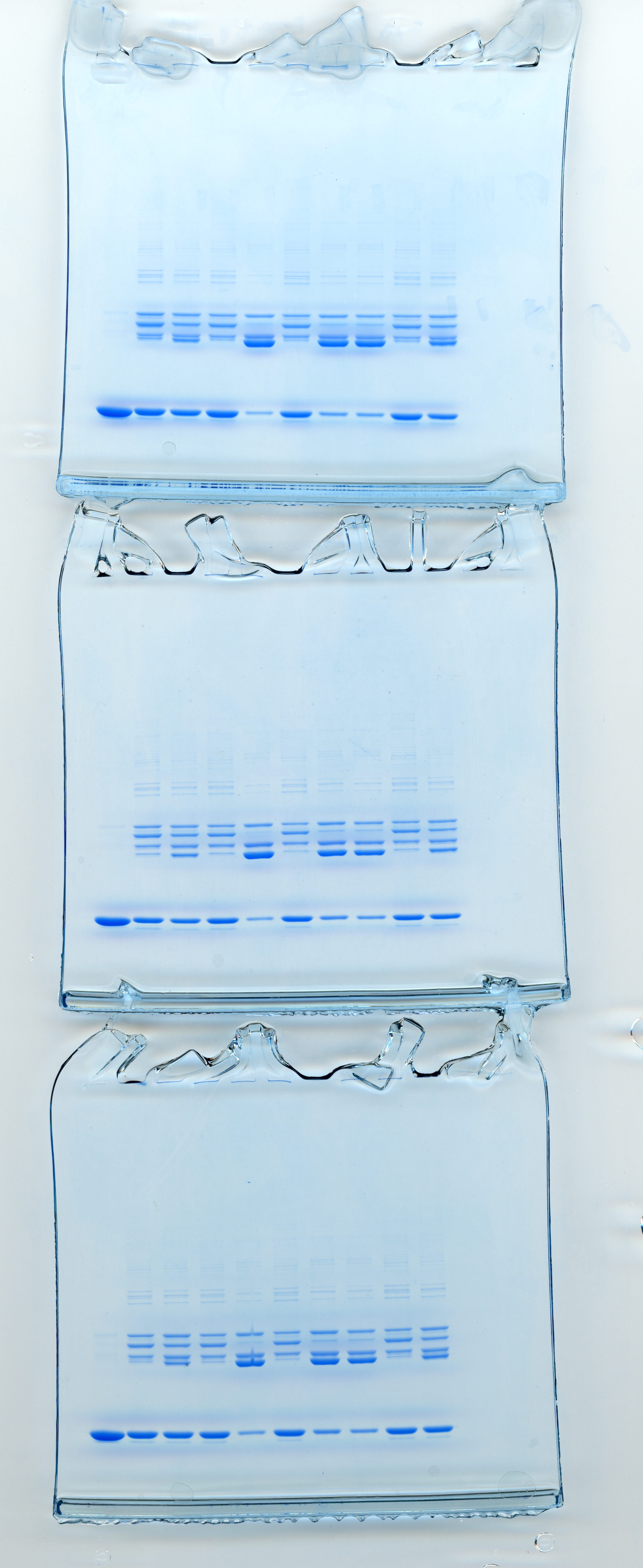

Supplement: Figure 5—figure supplement 2—source data 1. [file elife-69676-fig5-figsupp2-data1.zip › Figure5_figure_supplement2/DoubleXlink_N_DBD_lane1-12_Coomassie.tif]

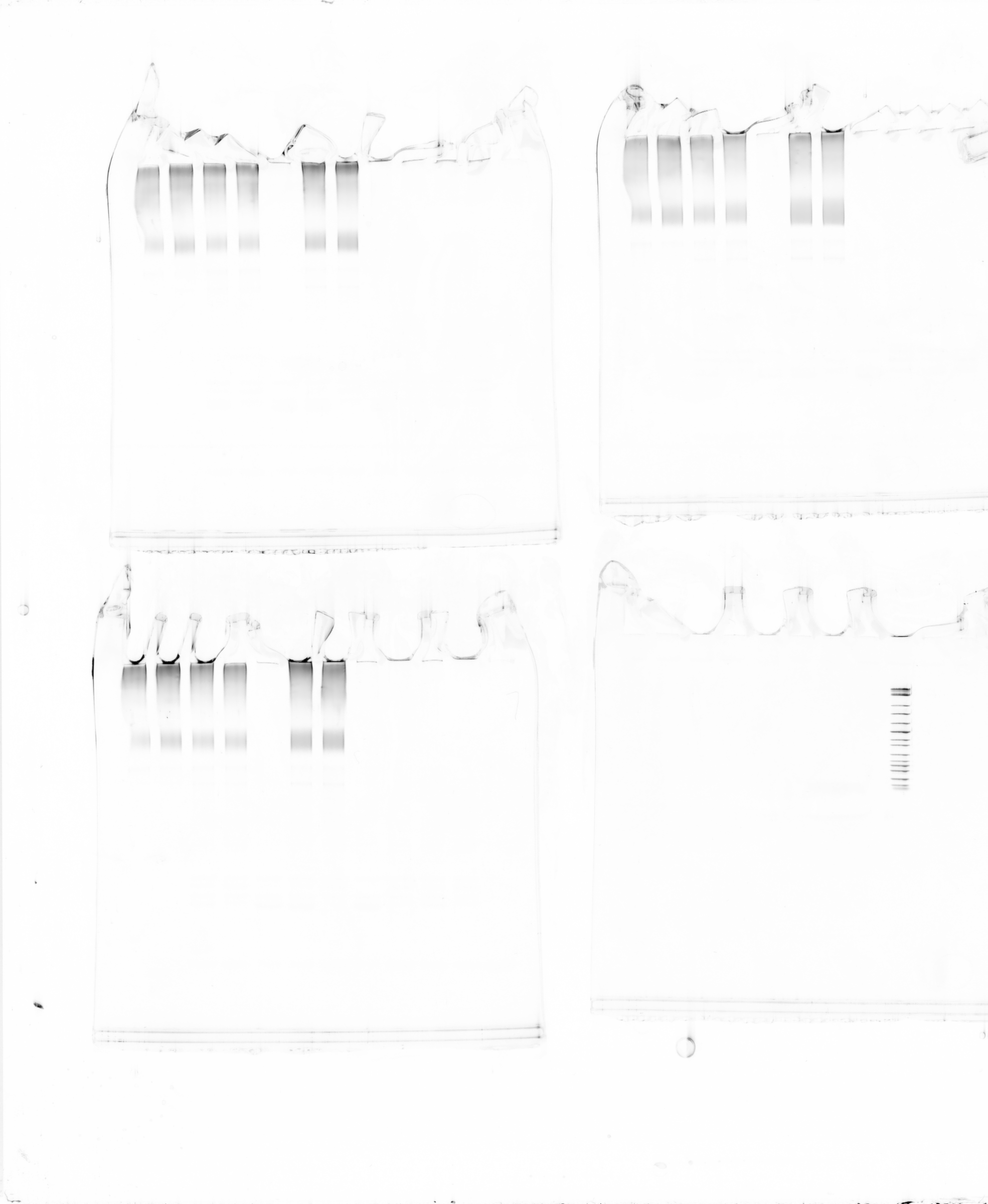

Supplement: Figure 5—figure supplement 2—source data 1. [file elife-69676-fig5-figsupp2-data1.zip › Figure5_figure_supplement2/DoubleXlink_N_DBD_sybrgreen.tif]

P<sub>W</sub>B(1224C I304C)-TEU

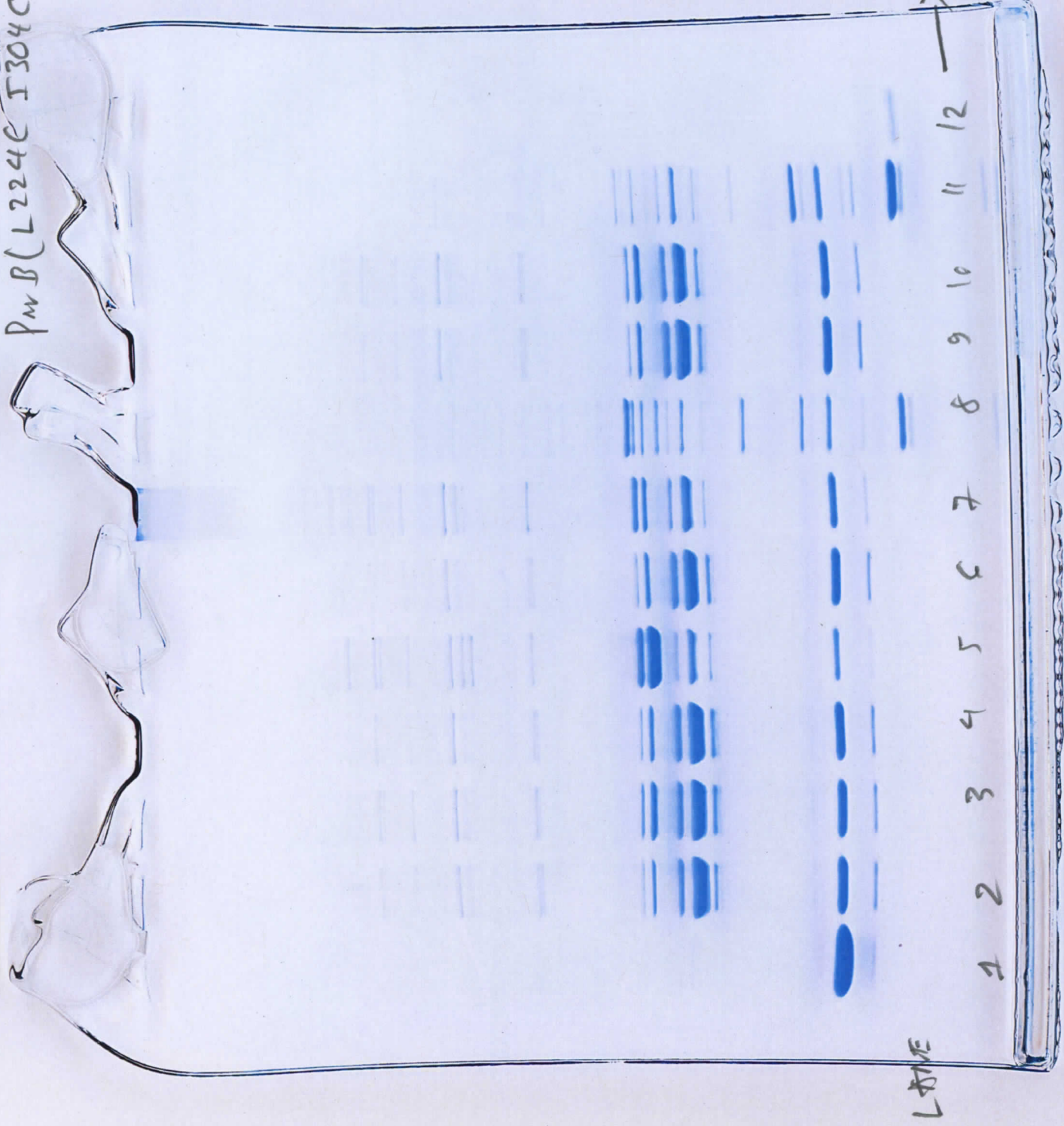

Lane #  
as appeared  
in figure.

Lane

1 2 3 4 5 6 7 8 9 10 11 12

Supplement: Figure 5—figure supplement 3—source data 1. [file elife-69676-fig5-figsupp3-data1.zip › Figure5_figure_supplement3/PanelA/Annotation.pdf]

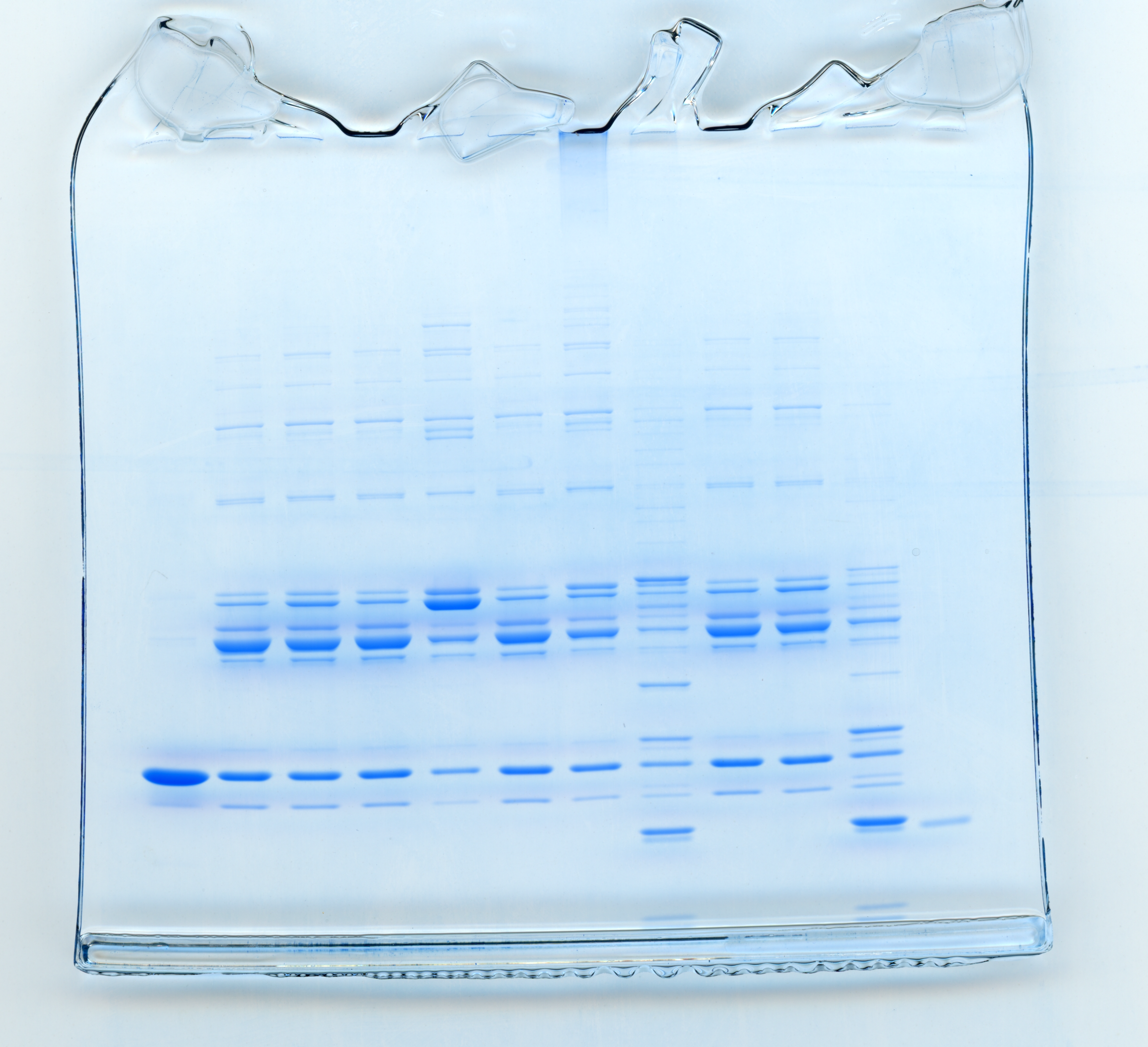

Supplement: Figure 5—figure supplement 3—source data 1. [file elife-69676-fig5-figsupp3-data1.zip › Figure5_figure_supplement3/PanelA/DoubleXlink_ParB_TEV.tif]

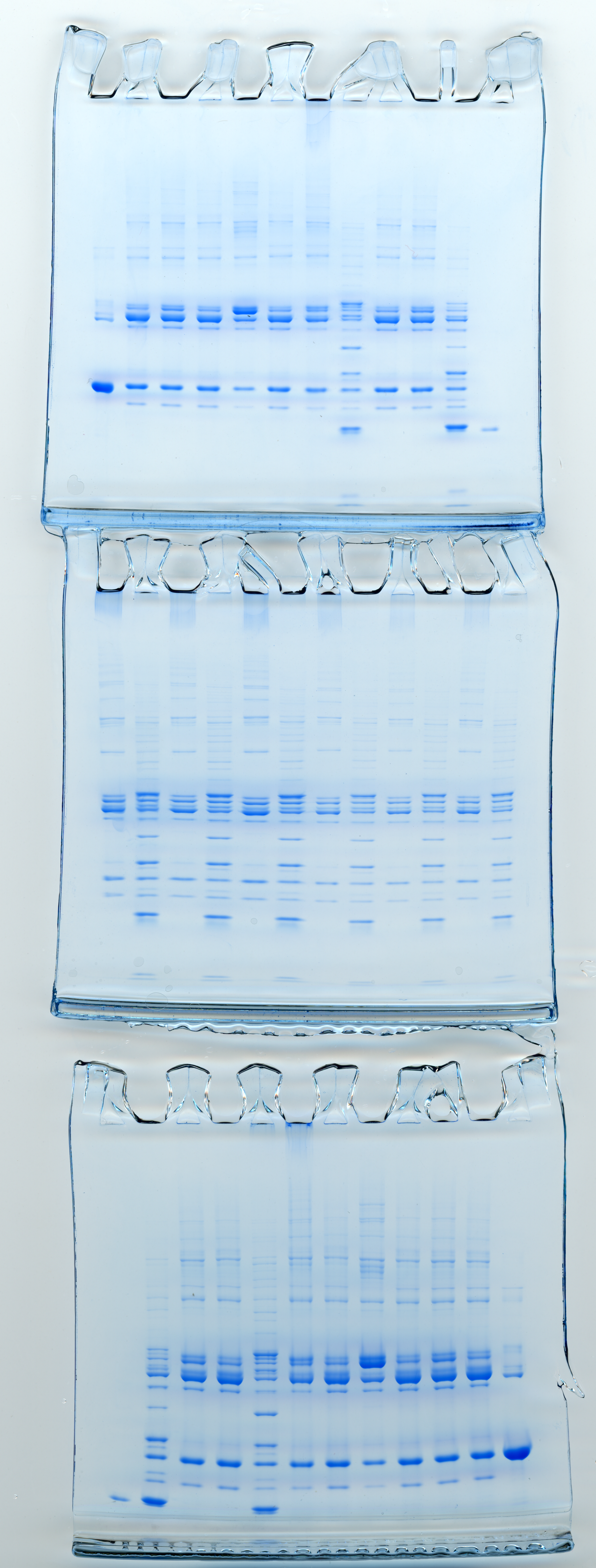

Supplement: Figure 5—figure supplement 3—source data 1. [file elife-69676-fig5-figsupp3-data1.zip › Figure5_figure_supplement3/PanelA/other_replicates/AcTEV_doubleXlink.tif]

Fig 5 - ~~fig~~ supp 3  
(A)

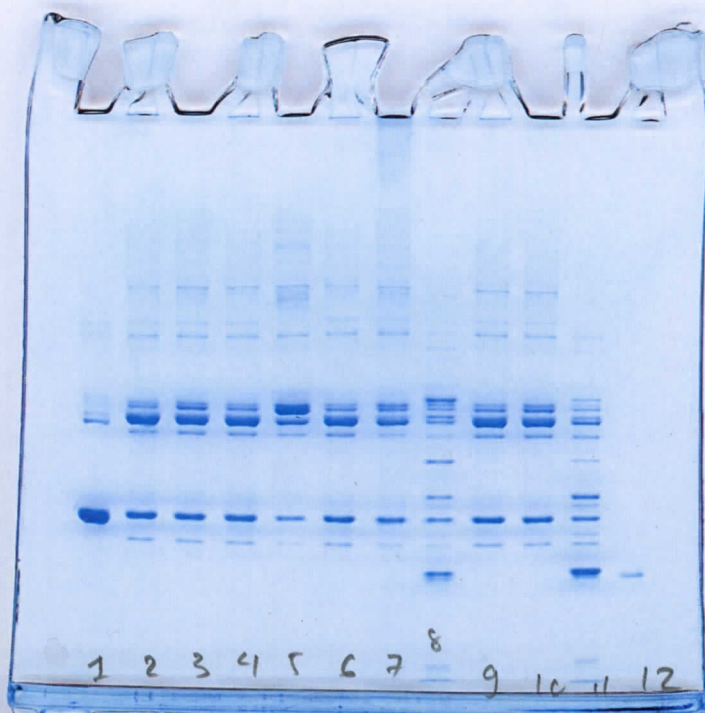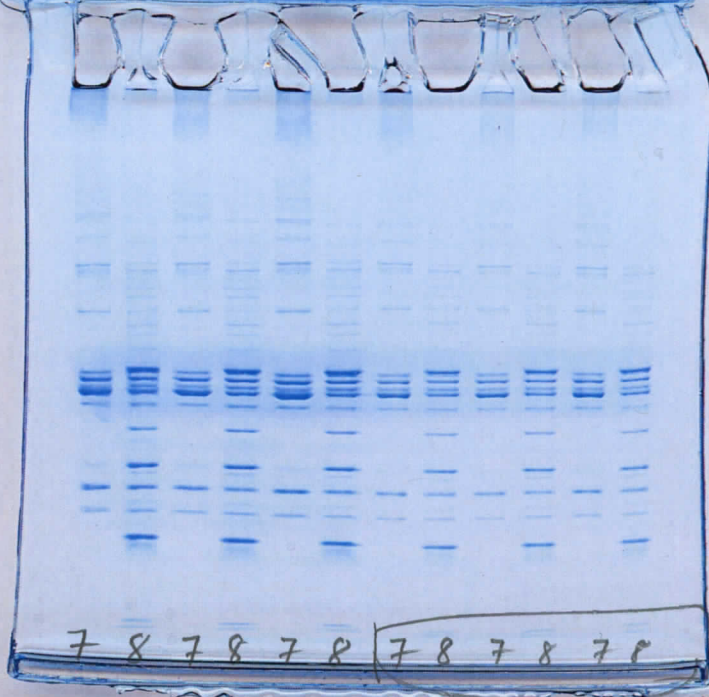

→ less volume loaded

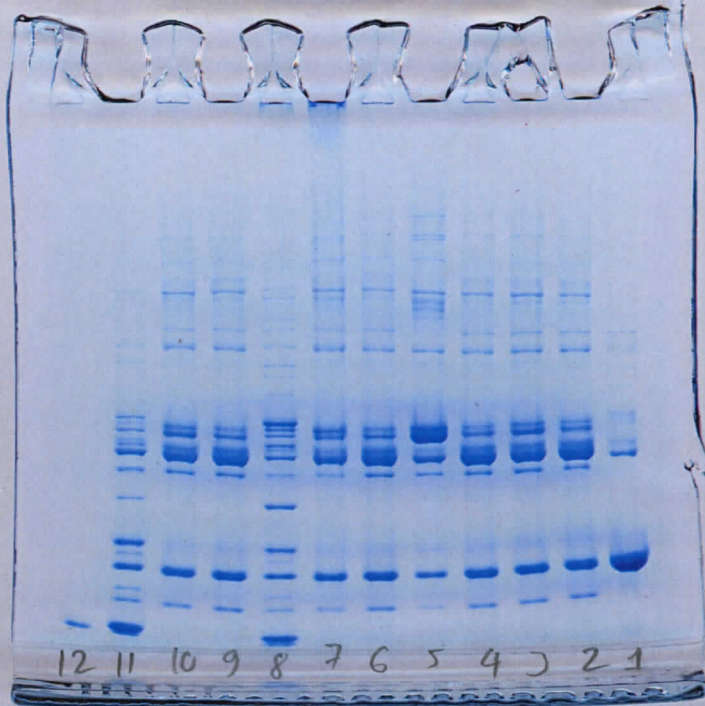

LANES (as in figure)

Supplement: Figure 5—figure supplement 3—source data 1. [file elife-69676-fig5-figsupp3-data1.zip › Figure5_figure_supplement3/PanelA/other_replicates/Annotation.pdf]

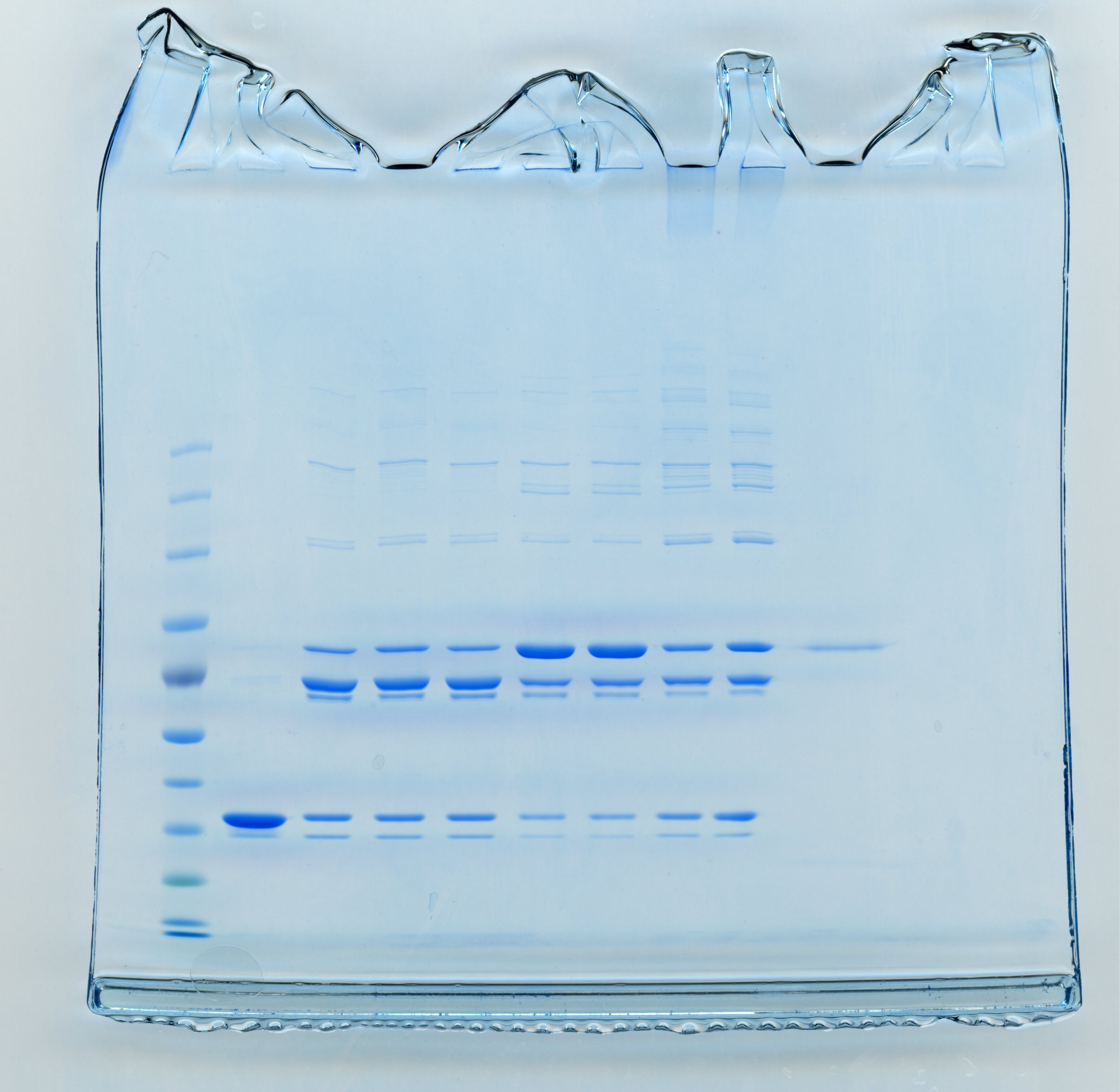

Supplement: Figure 5—figure supplement 3—source data 1. [file elife-69676-fig5-figsupp3-data1.zip › Figure5_figure_supplement3/PanelB/DoubleXlink_C_DBD_lane1-8_with_gelextract_lane9.tif]

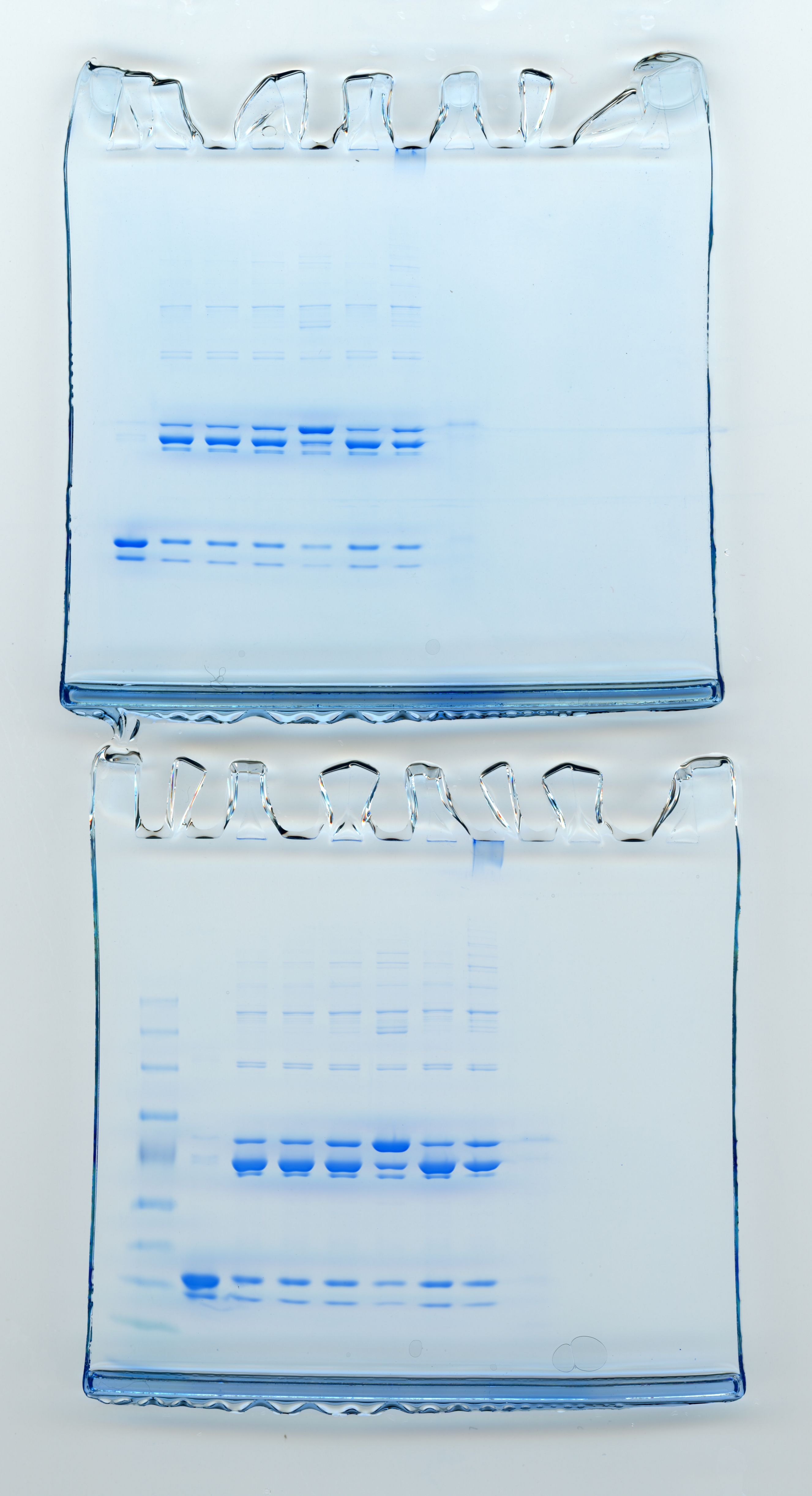

Supplement: Figure 5—figure supplement 3—source data 1. [file elife-69676-fig5-figsupp3-data1.zip › Figure5_figure_supplement3/PanelB/other_replicates/gelextract_doubleXlink.tif]

→ no protein control.

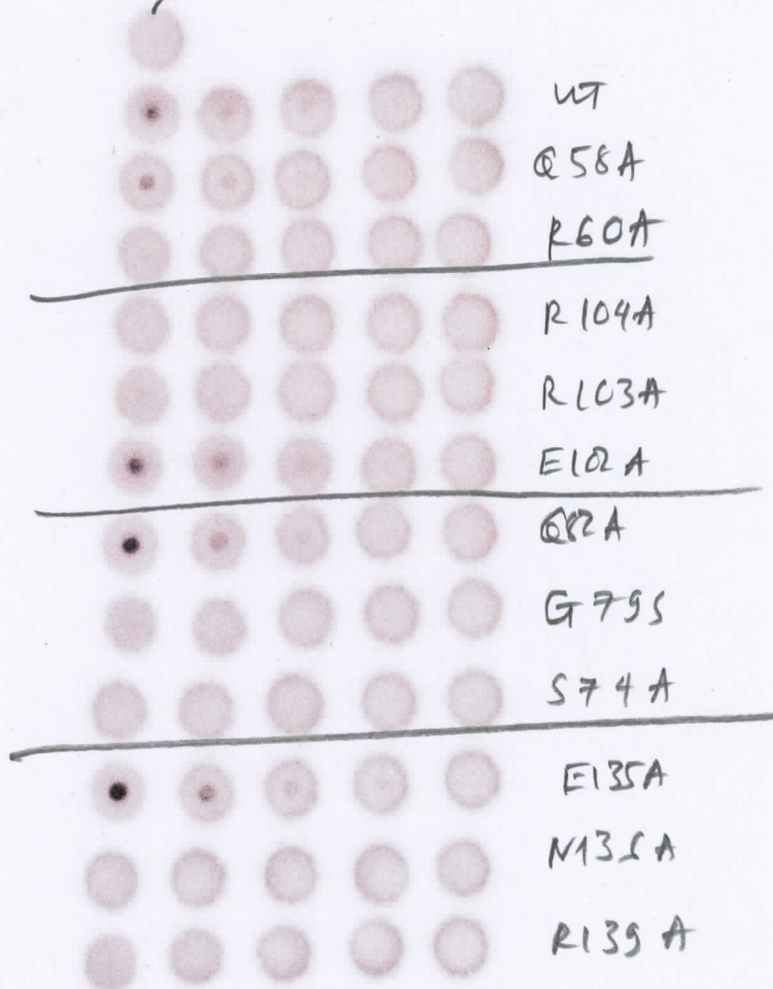

Supplement: Figure 6—source data 1. [file elife-69676-fig6-data1.zip › Figure6/PanelA/Annotation.pdf]

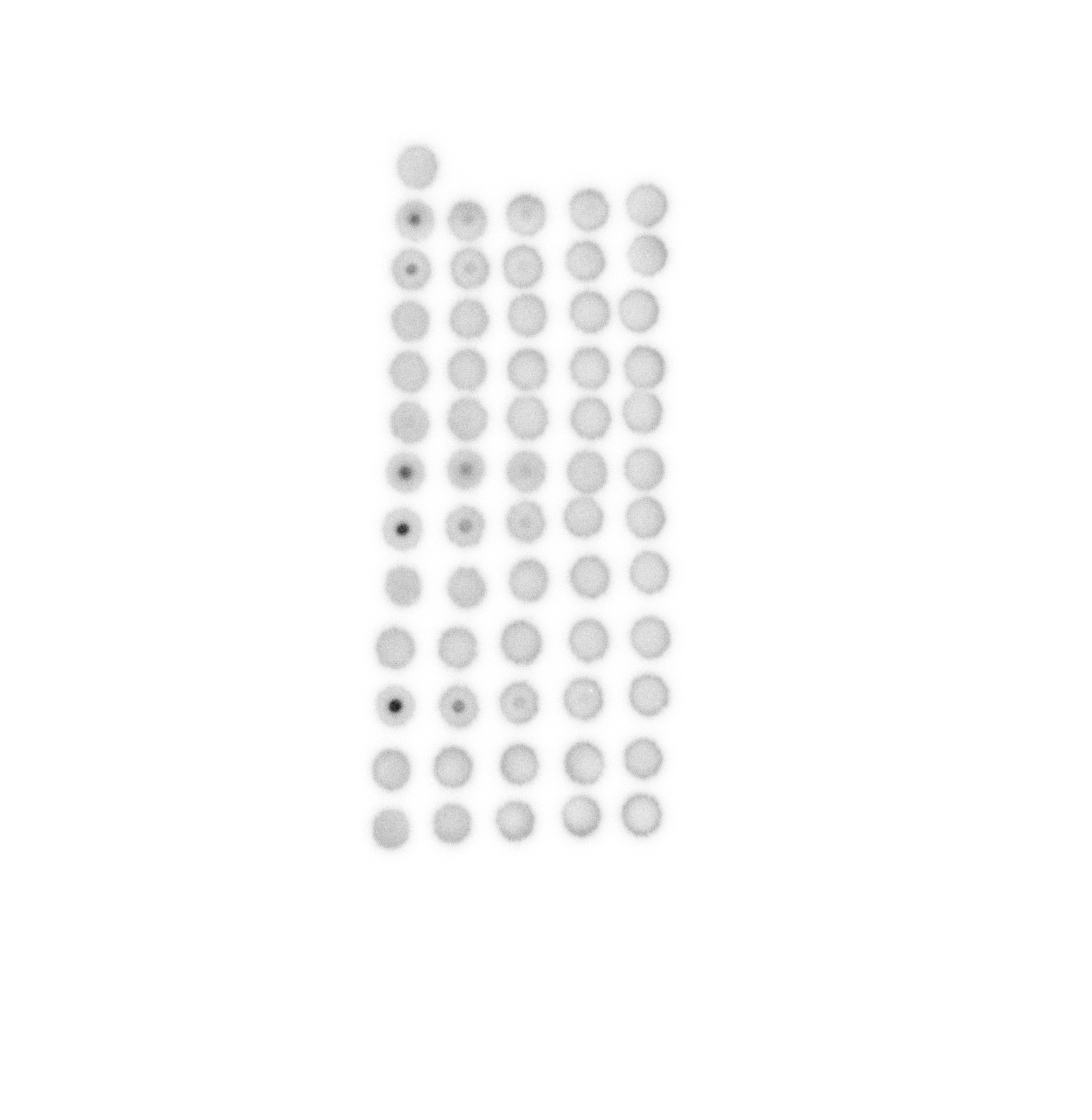

Supplement: Figure 6—source data 1. [file elife-69676-fig6-data1.zip › Figure6/PanelA/DRaCALA_assay.tif]

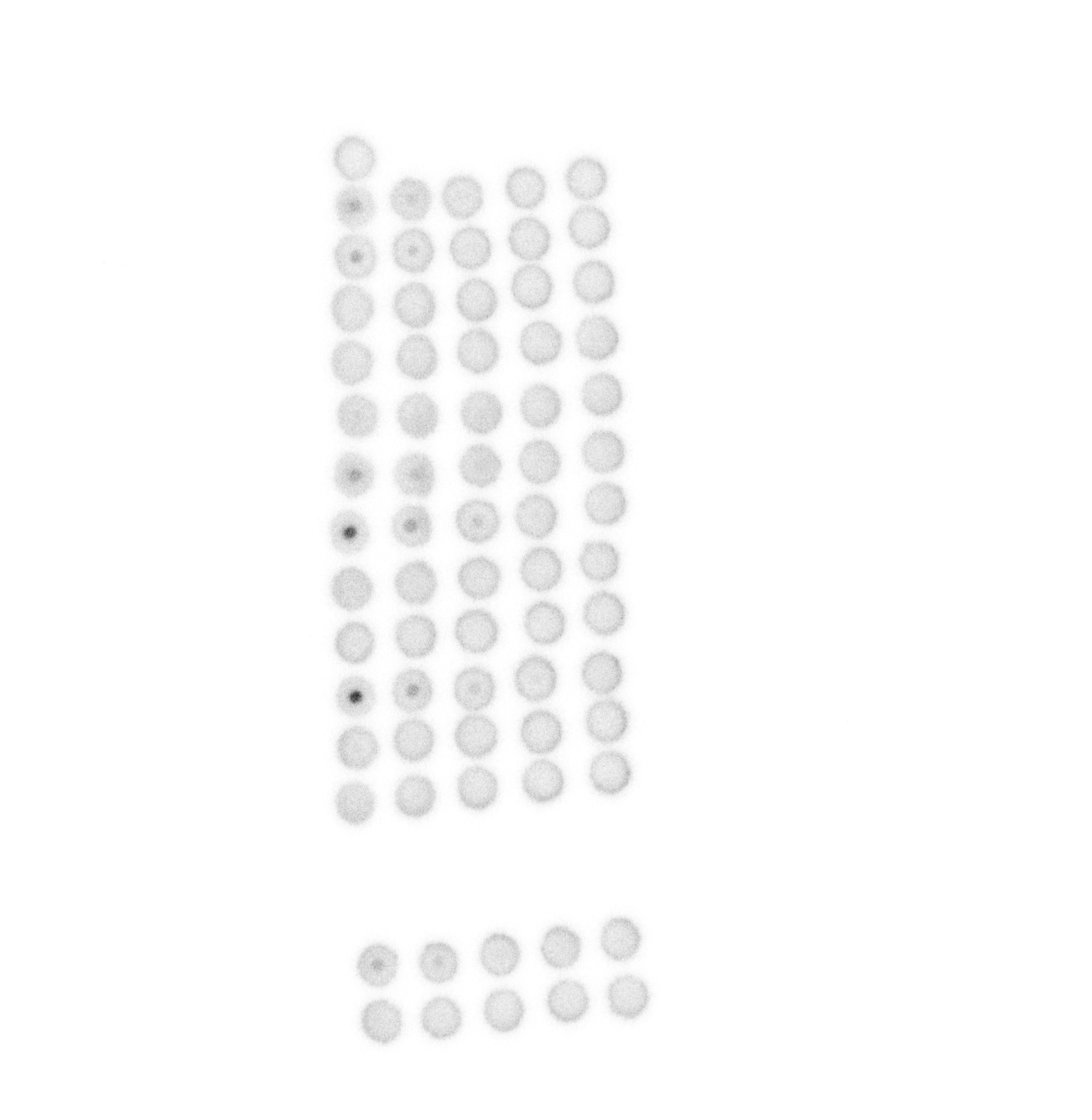

Supplement: Figure 6—source data 1. [file elife-69676-fig6-data1.zip › Figure6/PanelA/other_replicates/DraCALA_assay_rep2.jpg]

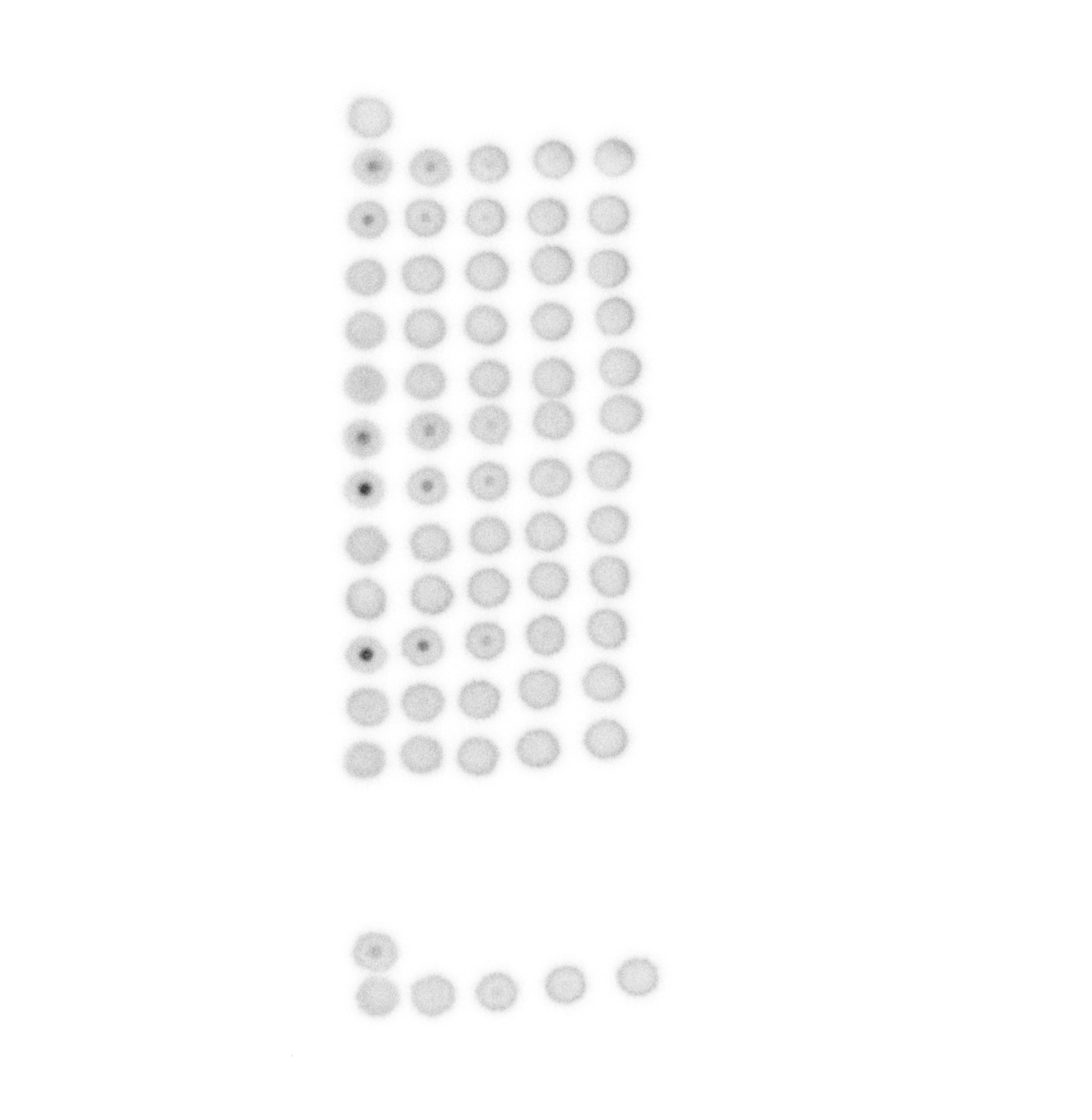

Supplement: Figure 6—source data 1. [file elife-69676-fig6-data1.zip › Figure6/PanelA/other_replicates/DraCALA_assay_rep3.jpg]

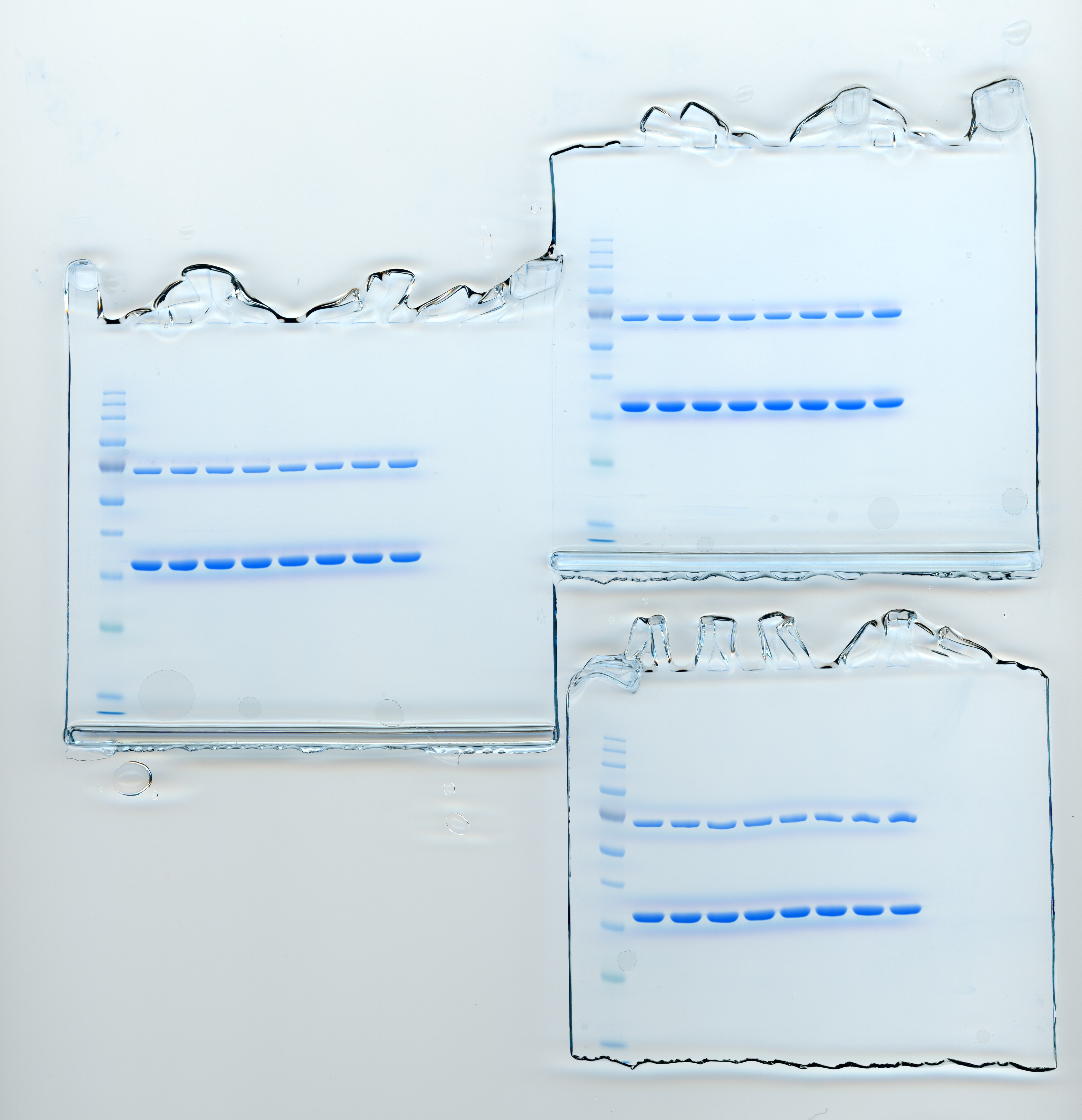

Supplement: Figure 6—figure supplement 1—source data 1. [file elife-69676-fig6-figsupp1-data1.zip › Figure6_figure_supplement1/Xlink_titration_1_010.jpg]

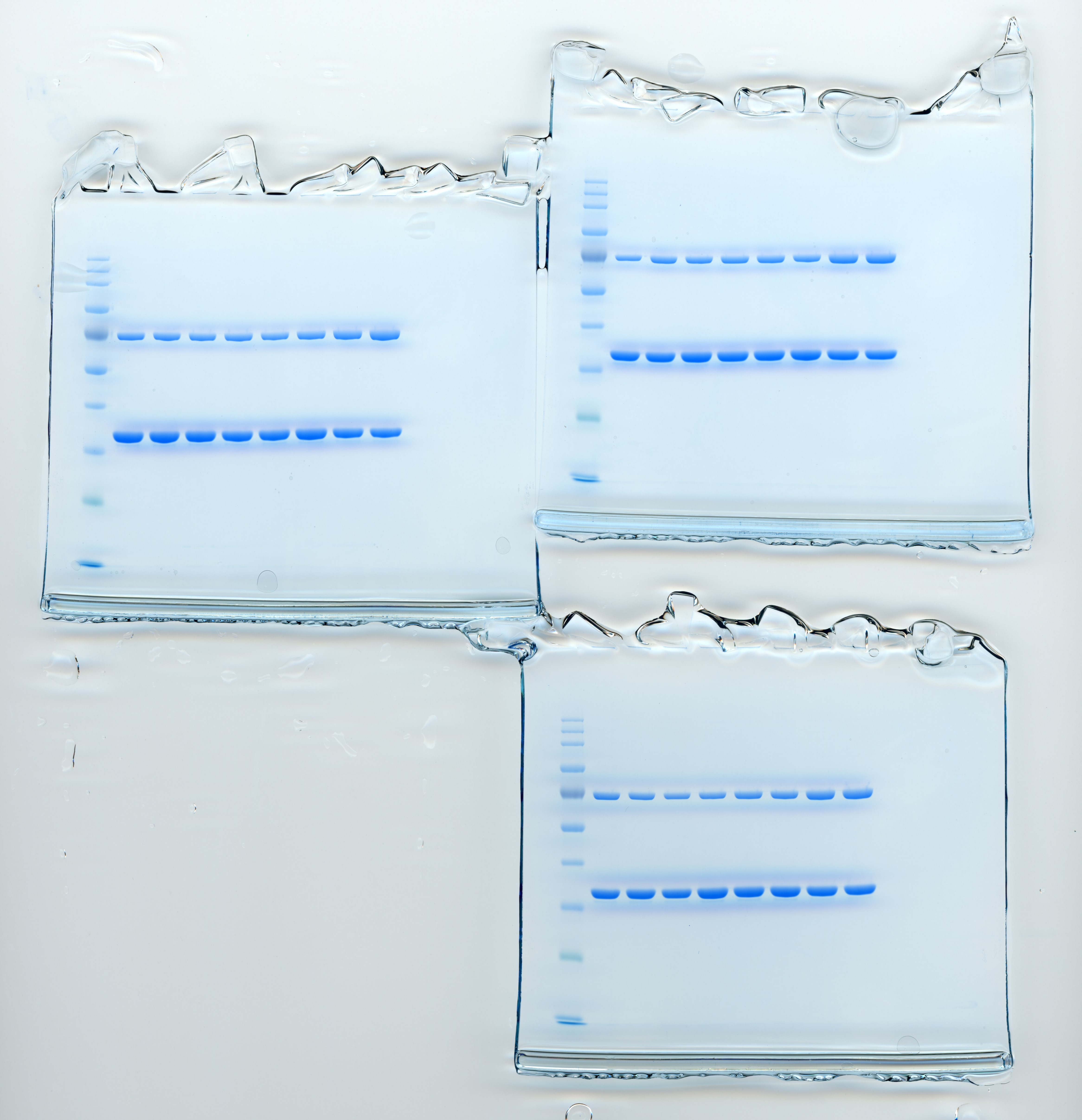

Supplement: Figure 6—figure supplement 1—source data 1. [file elife-69676-fig6-figsupp1-data1.zip › Figure6_figure_supplement1/Xlink_titration_10_002.jpg]

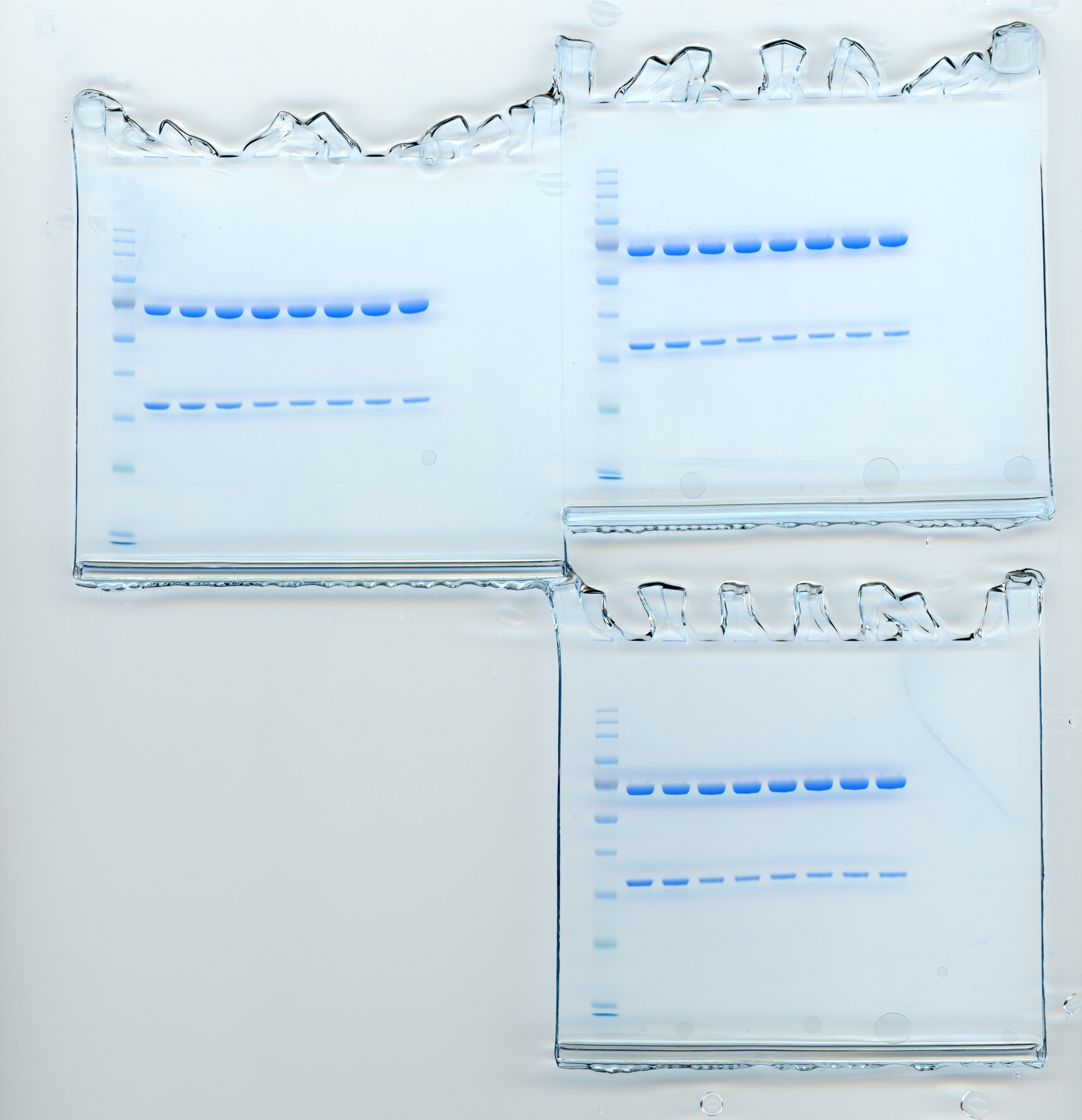

Supplement: Figure 6—figure supplement 1—source data 1. [file elife-69676-fig6-figsupp1-data1.zip › Figure6_figure_supplement1/Xlink_titration_11_007.jpg]

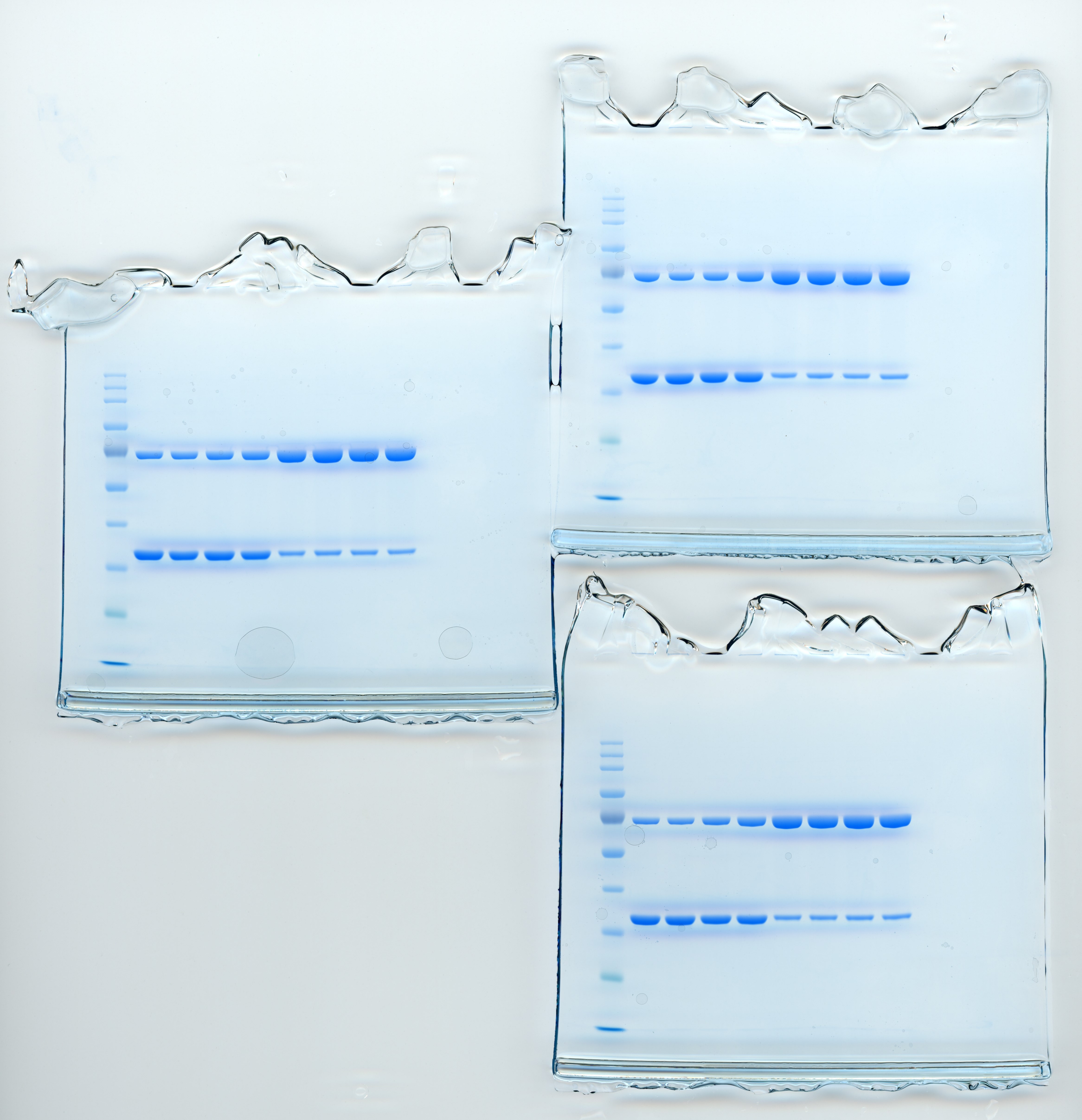

Supplement: Figure 6—figure supplement 1—source data 1. [file elife-69676-fig6-figsupp1-data1.zip › Figure6_figure_supplement1/Xlink_titration_13_001.jpg]

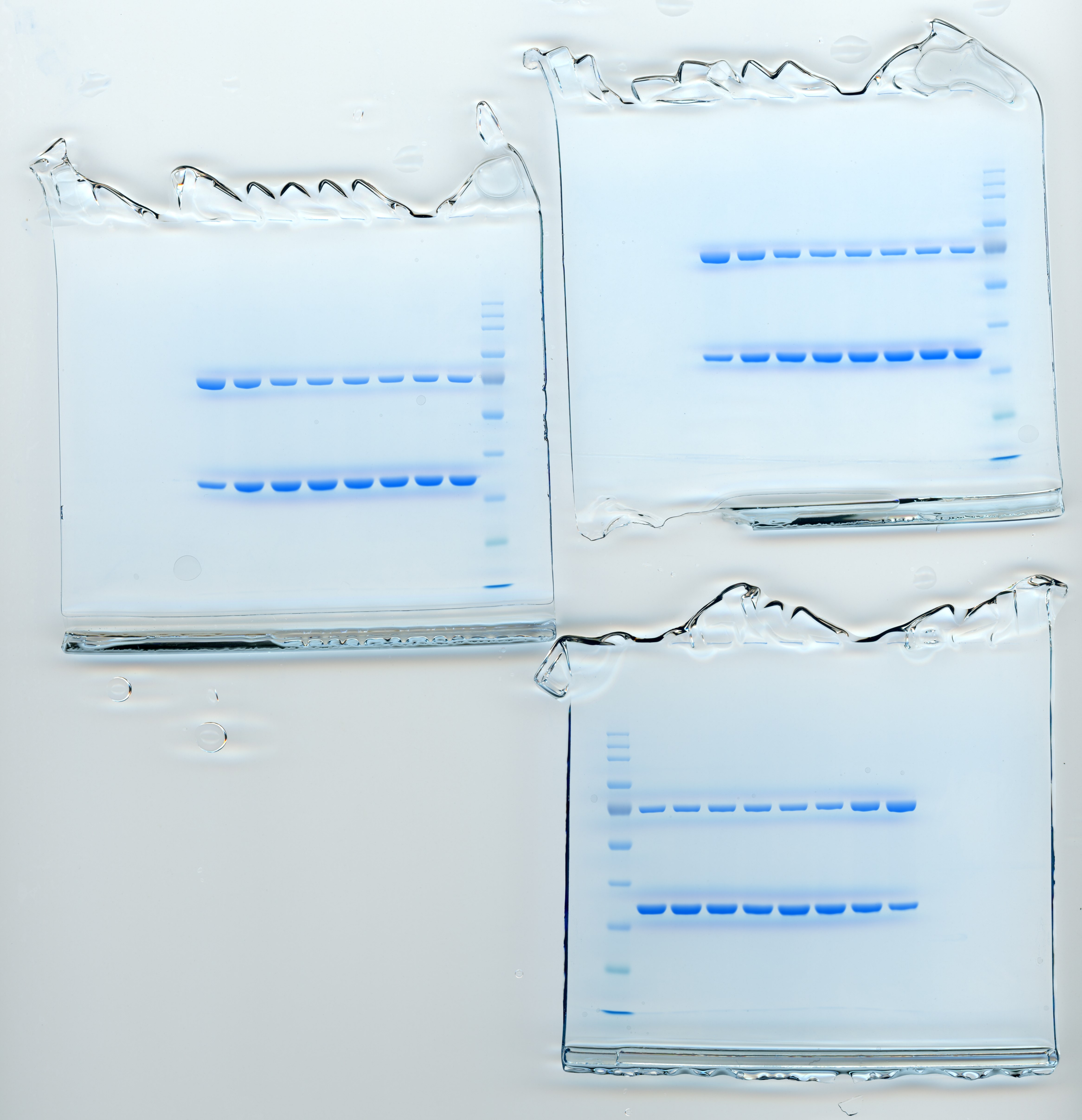

Supplement: Figure 6—figure supplement 1—source data 1. [file elife-69676-fig6-figsupp1-data1.zip › Figure6_figure_supplement1/Xlink_titration_14_003.jpg]

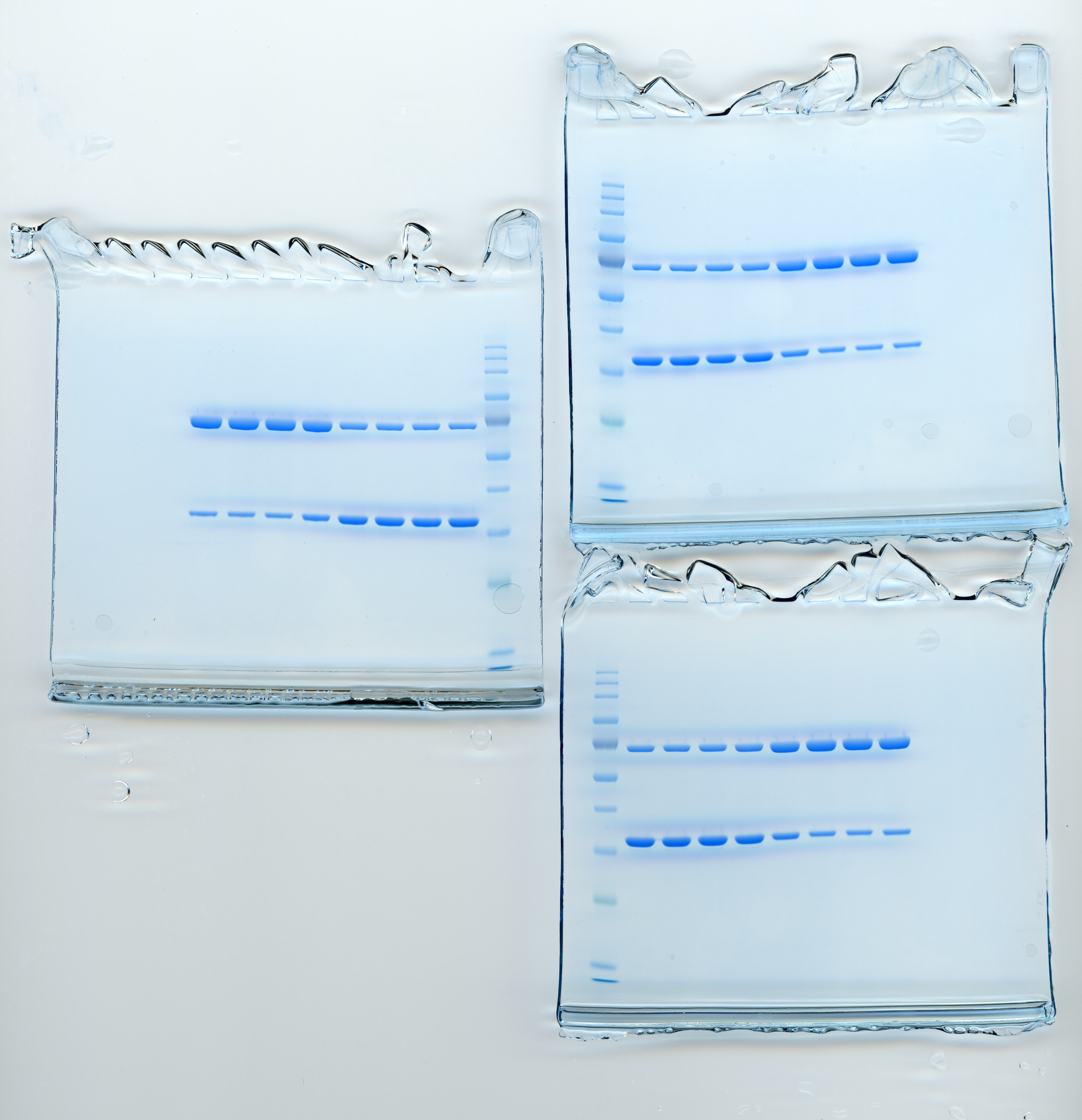

Supplement: Figure 6—figure supplement 1—source data 1. [file elife-69676-fig6-figsupp1-data1.zip › Figure6_figure_supplement1/Xlink_titration_2_004.jpg]

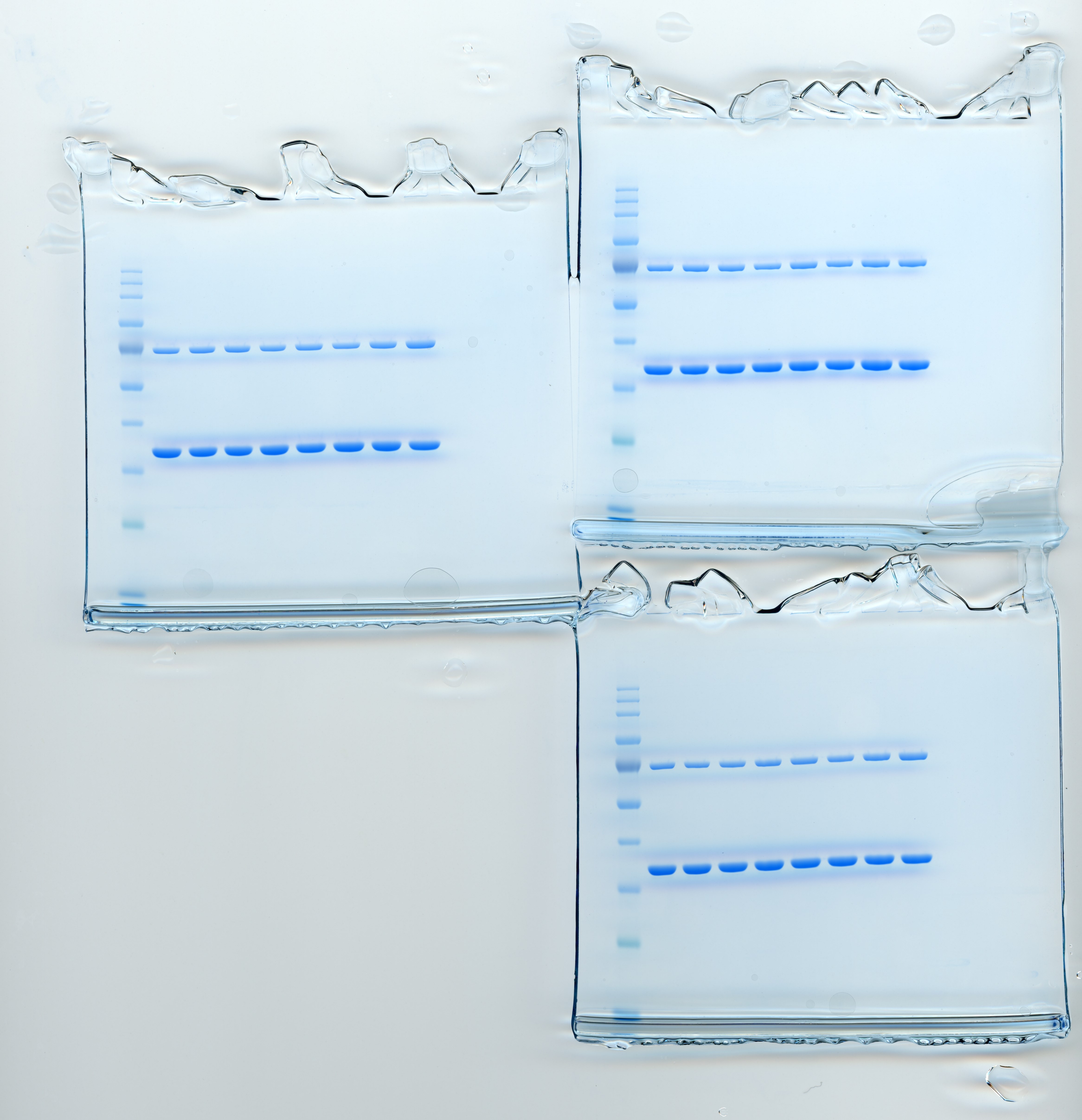

Supplement: Figure 6—figure supplement 1—source data 1. [file elife-69676-fig6-figsupp1-data1.zip › Figure6_figure_supplement1/Xlink_titration_3_005.jpg]

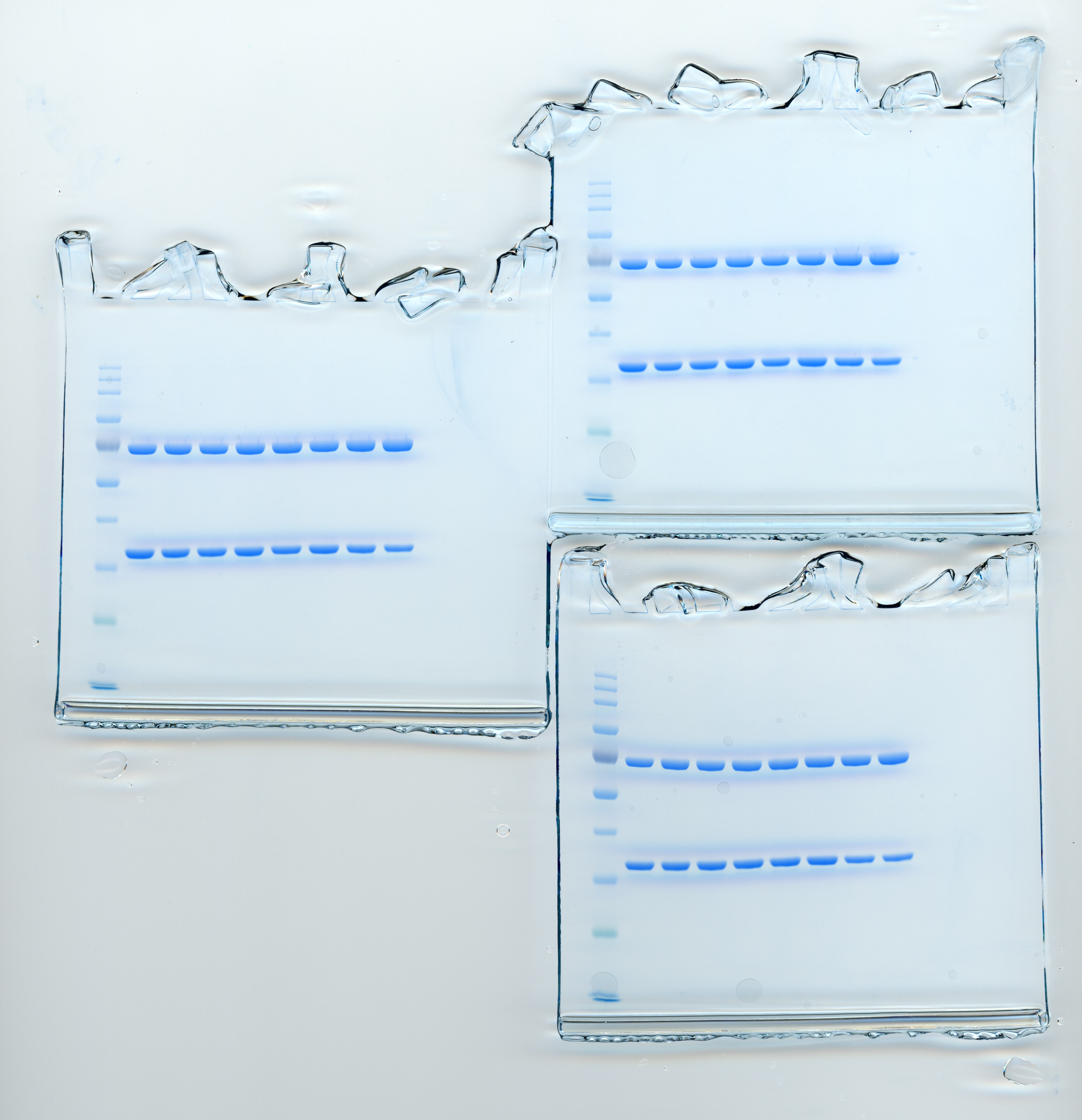

Supplement: Figure 6—figure supplement 1—source data 1. [file elife-69676-fig6-figsupp1-data1.zip › Figure6_figure_supplement1/Xlink_titration_4_008.jpg]

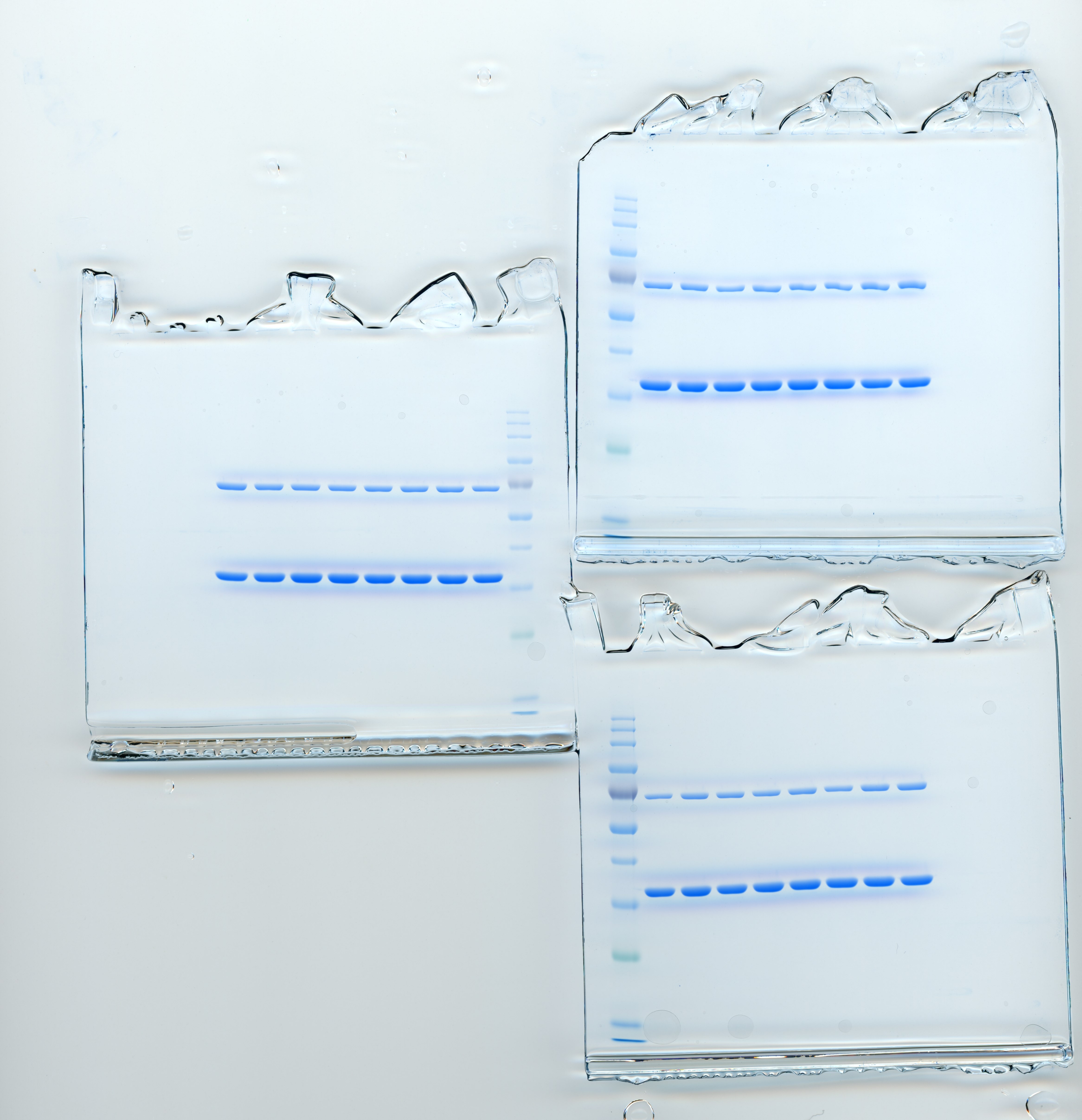

Supplement: Figure 6—figure supplement 1—source data 1. [file elife-69676-fig6-figsupp1-data1.zip › Figure6_figure_supplement1/Xlink_titration_5_009.jpg]

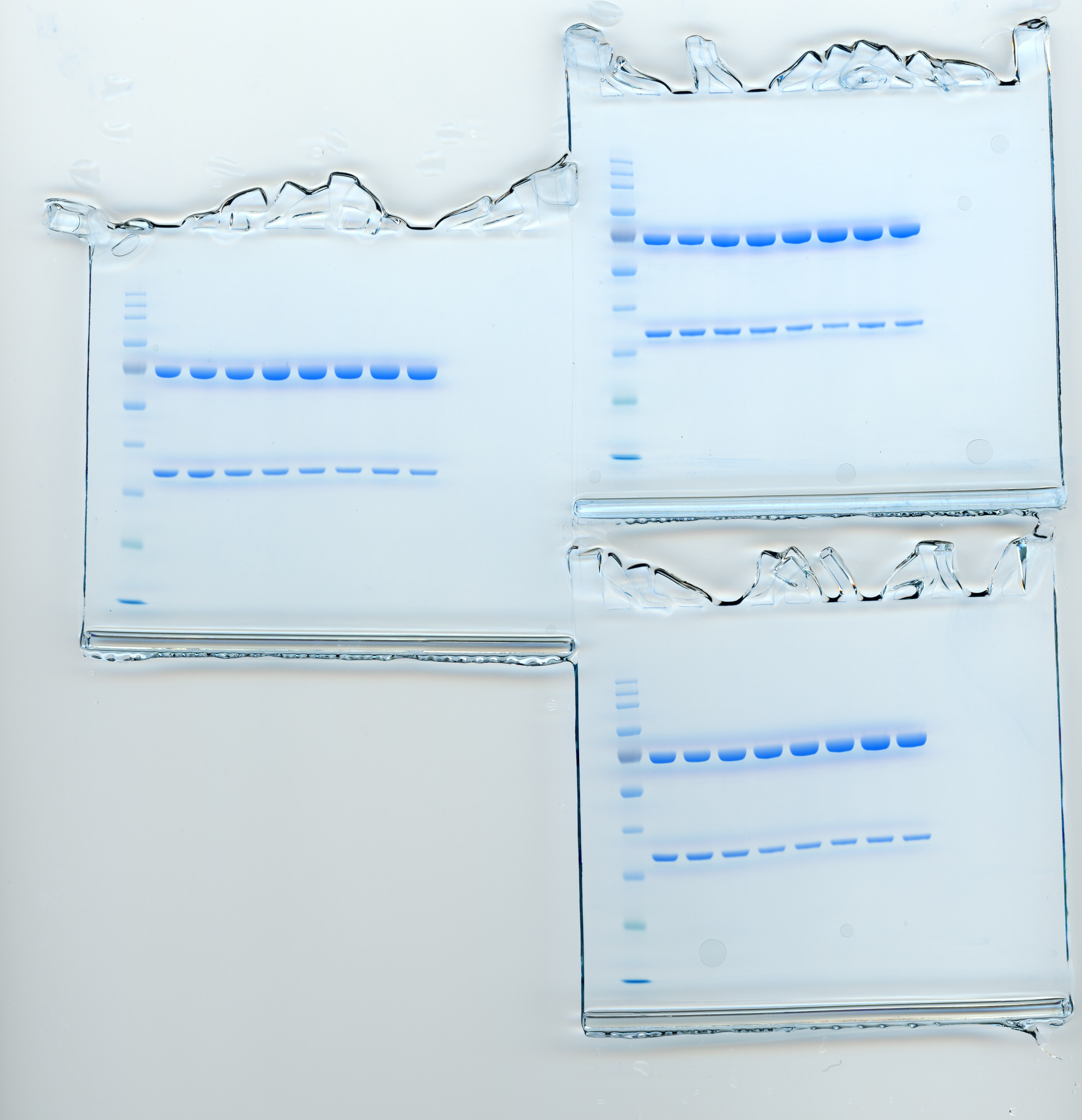

Supplement: Figure 6—figure supplement 1—source data 1. [file elife-69676-fig6-figsupp1-data1.zip › Figure6_figure_supplement1/Xlink_titration_7_006.jpg]

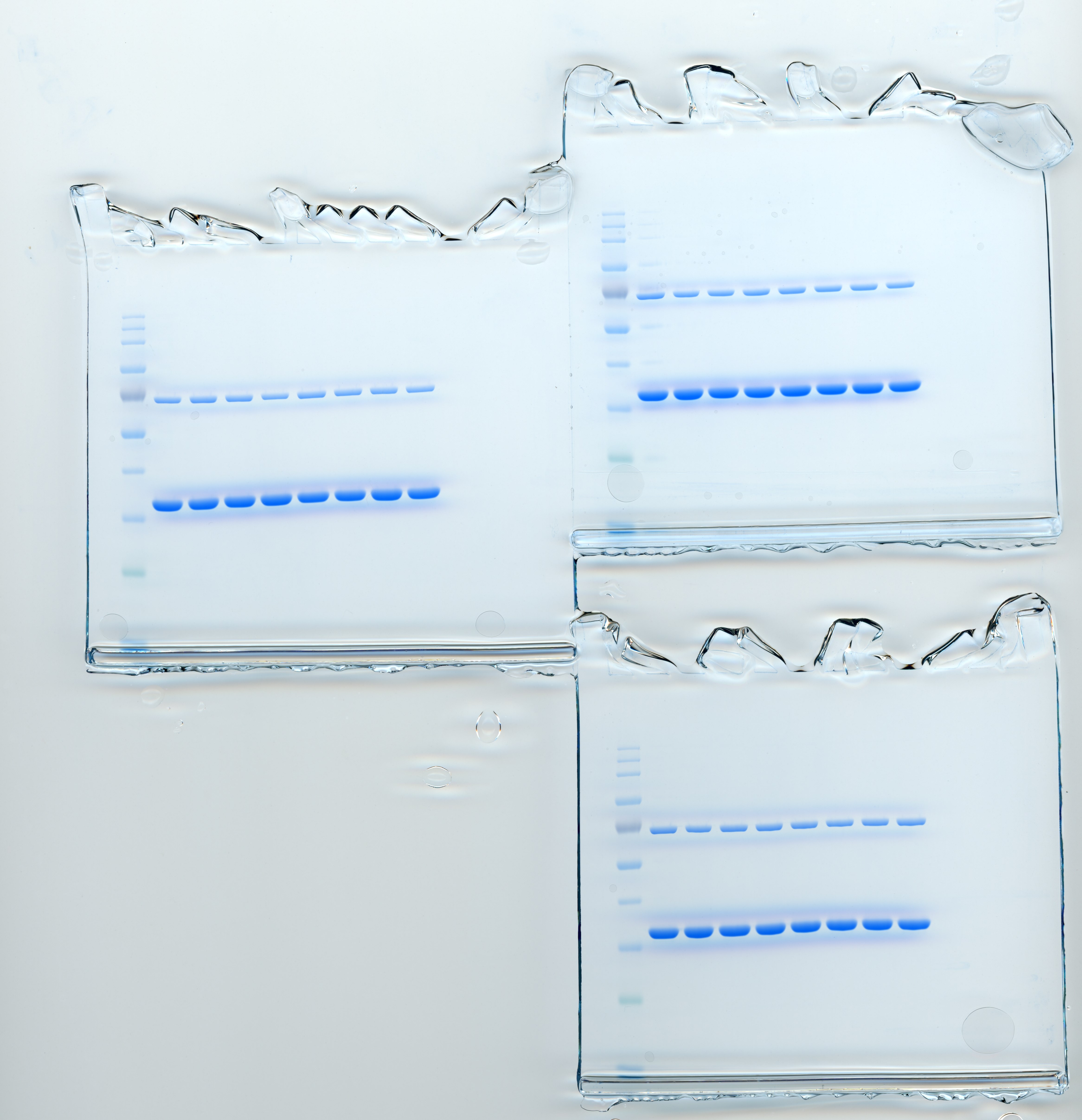

Supplement: Figure 6—figure supplement 1—source data 1. [file elife-69676-fig6-figsupp1-data1.zip › Figure6_figure_supplement1/Xlink_titration_R104A_011.jpg]

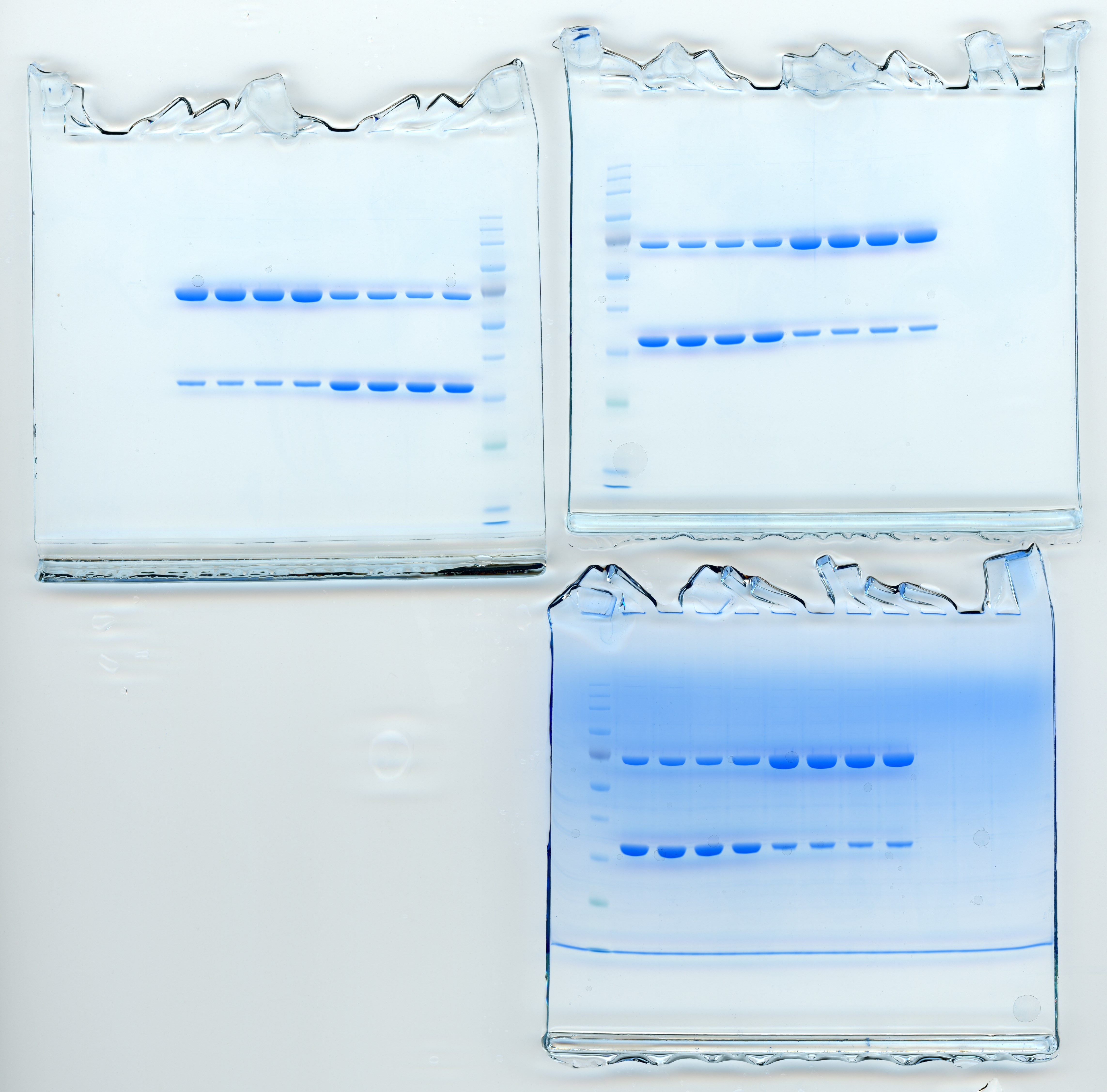

Supplement: Figure 6—figure supplement 1—source data 1. [file elife-69676-fig6-figsupp1-data1.zip › Figure6_figure_supplement1/Xlink_titration_WT_001.jpg]

(A)

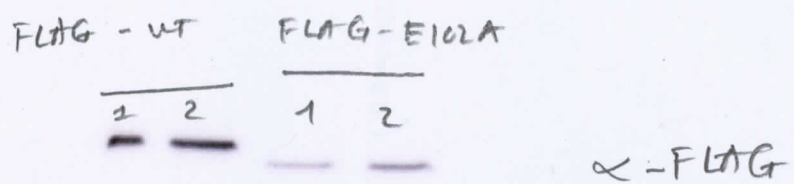

Coomassie

|       |   |      |       |
|-------|---|------|-------|
| 1     | 2 |      |       |
| <hr/> |   | FLAG | FLAG  |
|       |   | WT   | F102A |

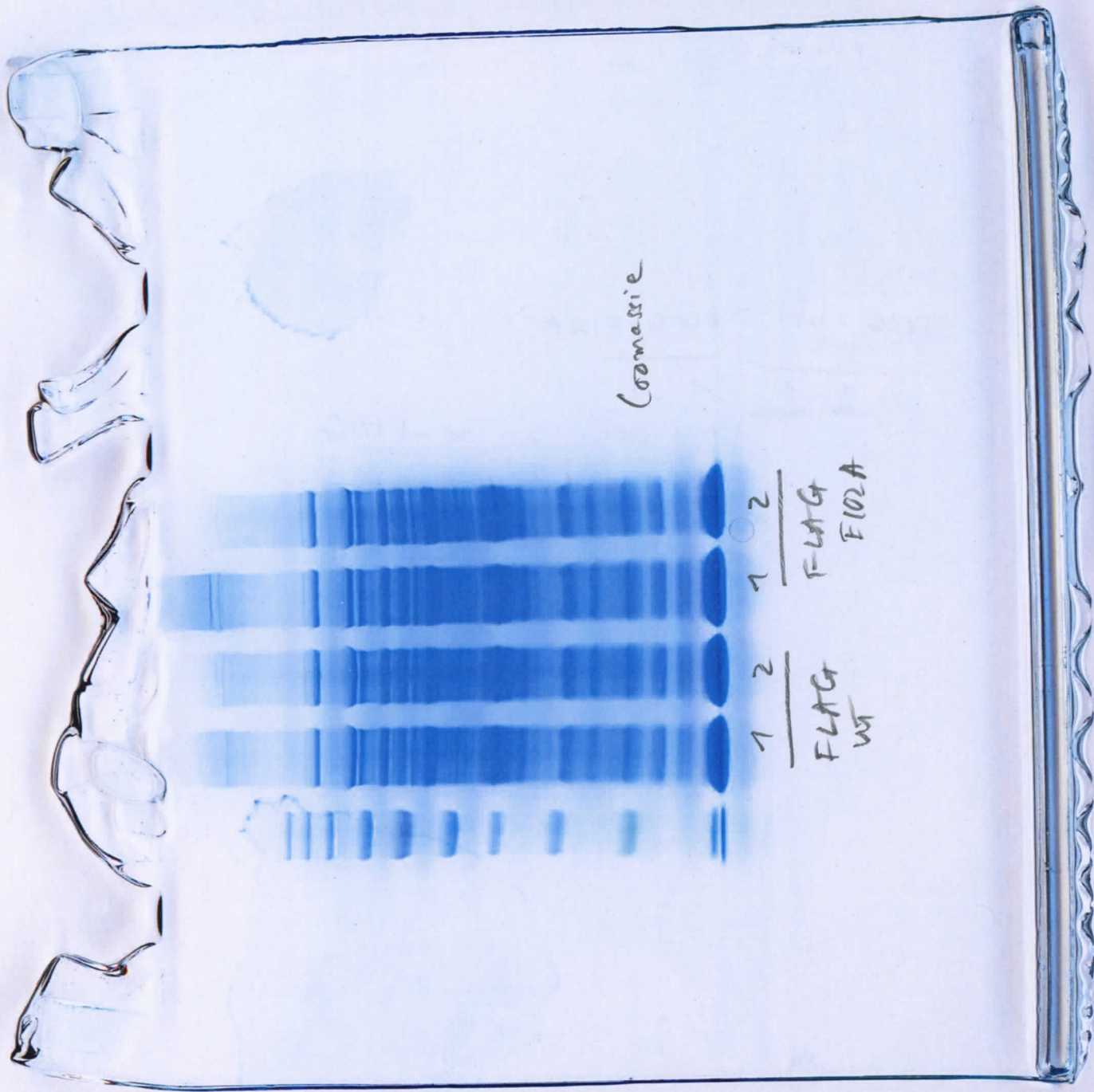

Supplement: Figure 8—figure supplement 1—source data 1. [file elife-69676-fig8-figsupp1-data1.zip › Figure8_figure_supplement1/PanelA/Annotation.pdf]

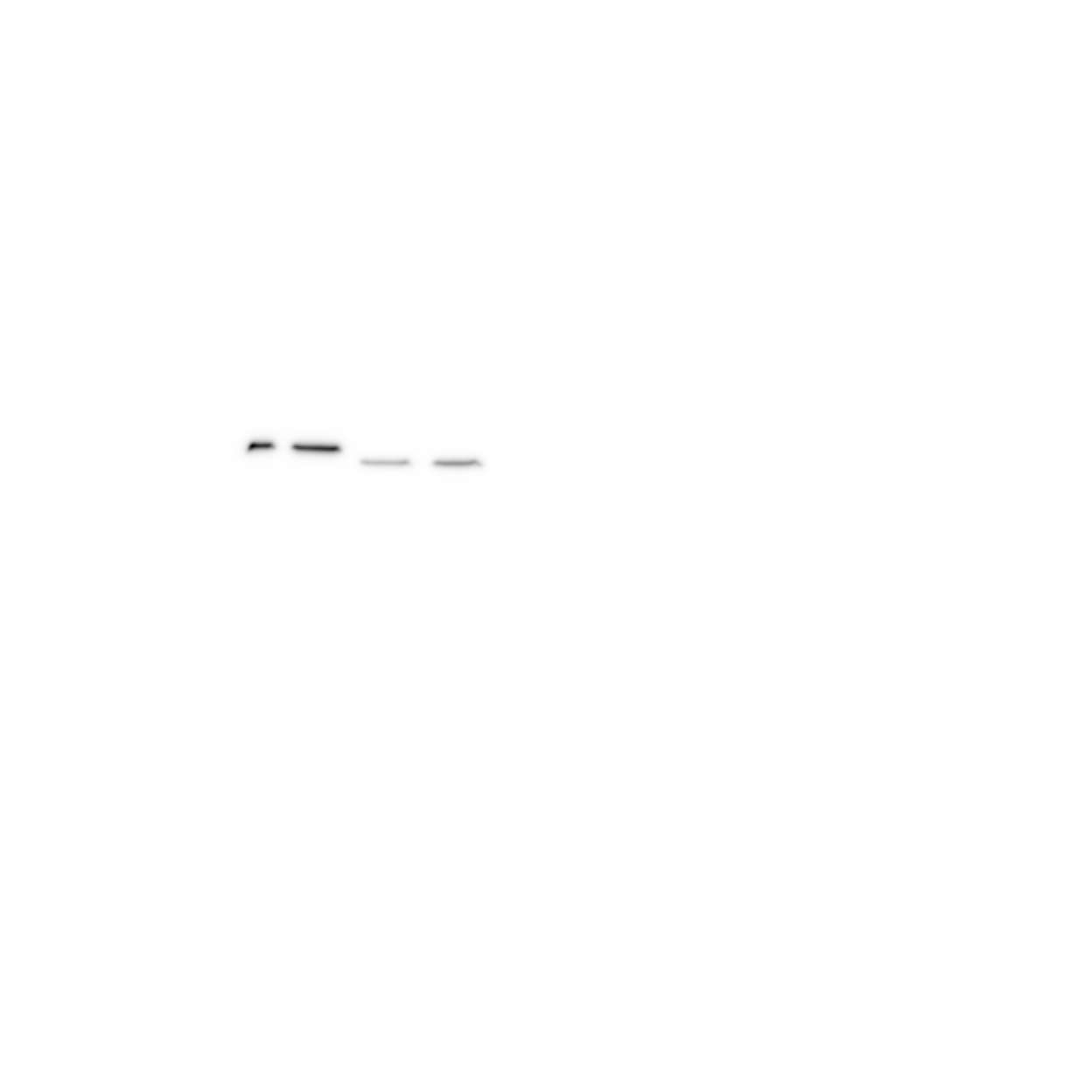

Supplement: Figure 8—figure supplement 1—source data 1. [file elife-69676-fig8-figsupp1-data1.zip › Figure8_figure_supplement1/PanelA/WB_parB 20210315_151332_Ch.tif]

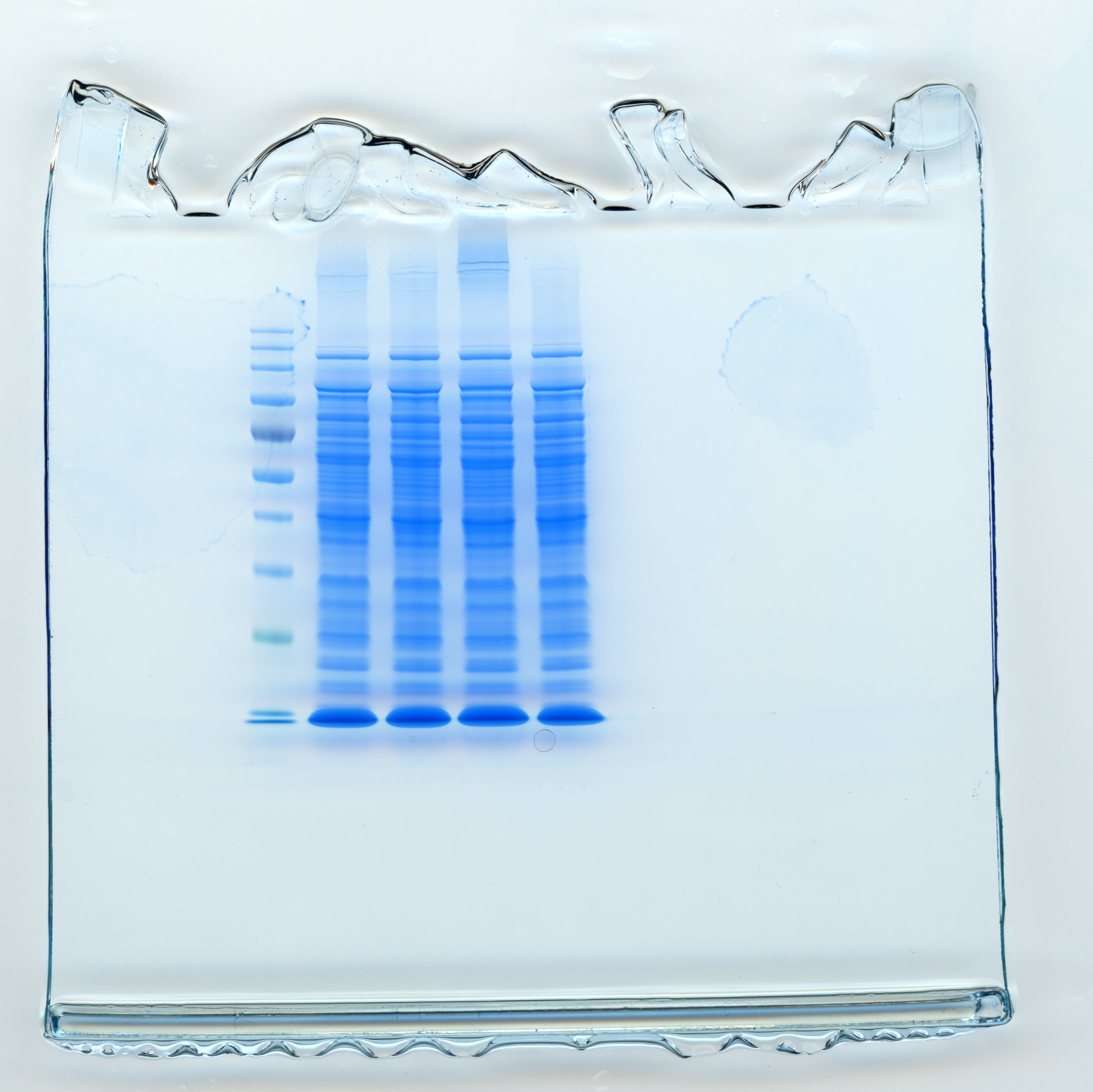

Supplement: Figure 8—figure supplement 1—source data 1. [file elife-69676-fig8-figsupp1-data1.zip › Figure8_figure_supplement1/PanelA/WB_WtvsE102A_Coomassie.tif]

CFP-ParB(wt)    CFP-ParB(E102A)

1    2    1    2

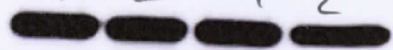

← non-specific band

λ-GFD

] GFP-ParB

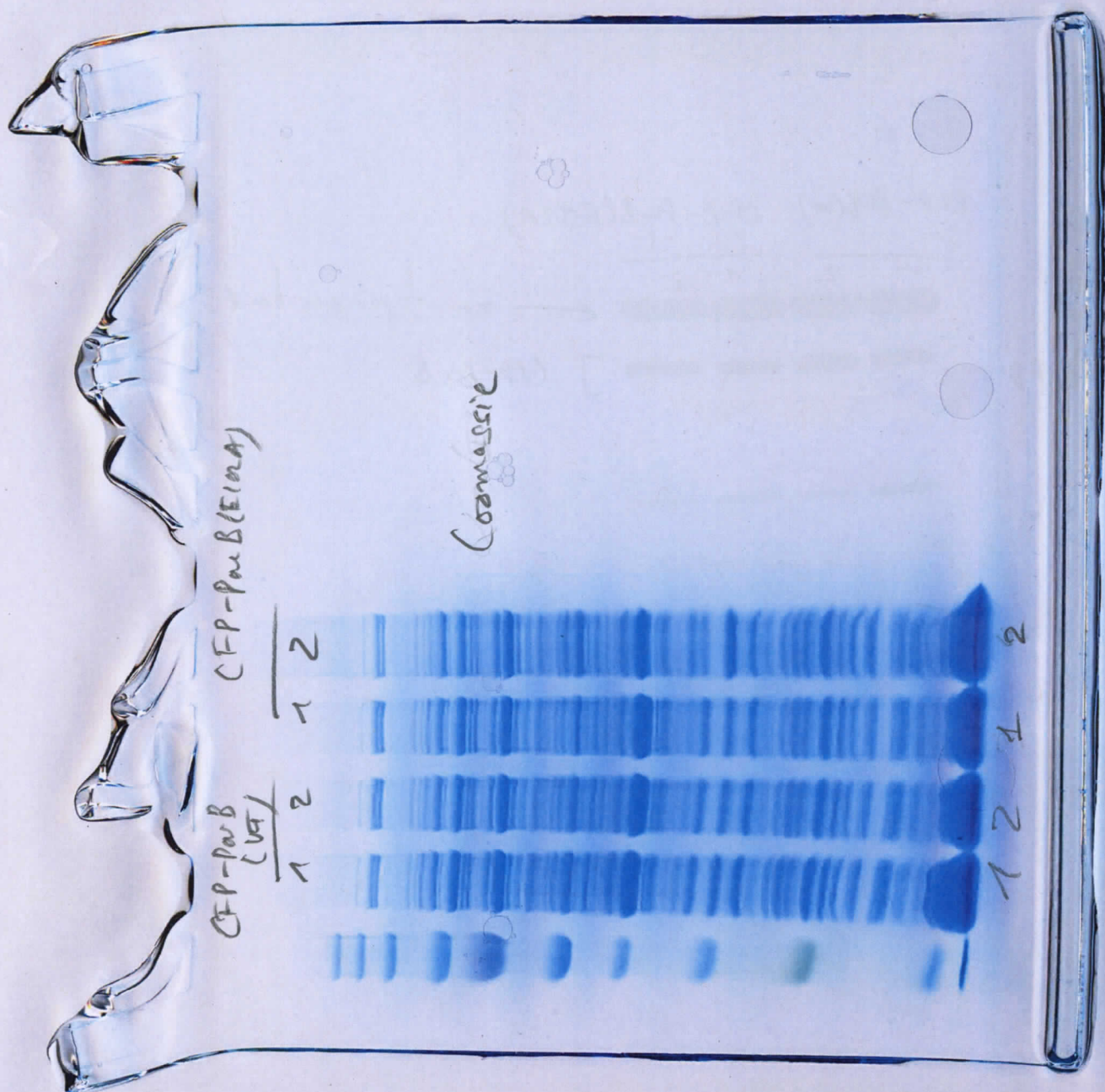

Supplement: Figure 8—figure supplement 1—source data 1. [file elife-69676-fig8-figsupp1-data1.zip › Figure8_figure_supplement1/PanelB/Annotation.pdf]

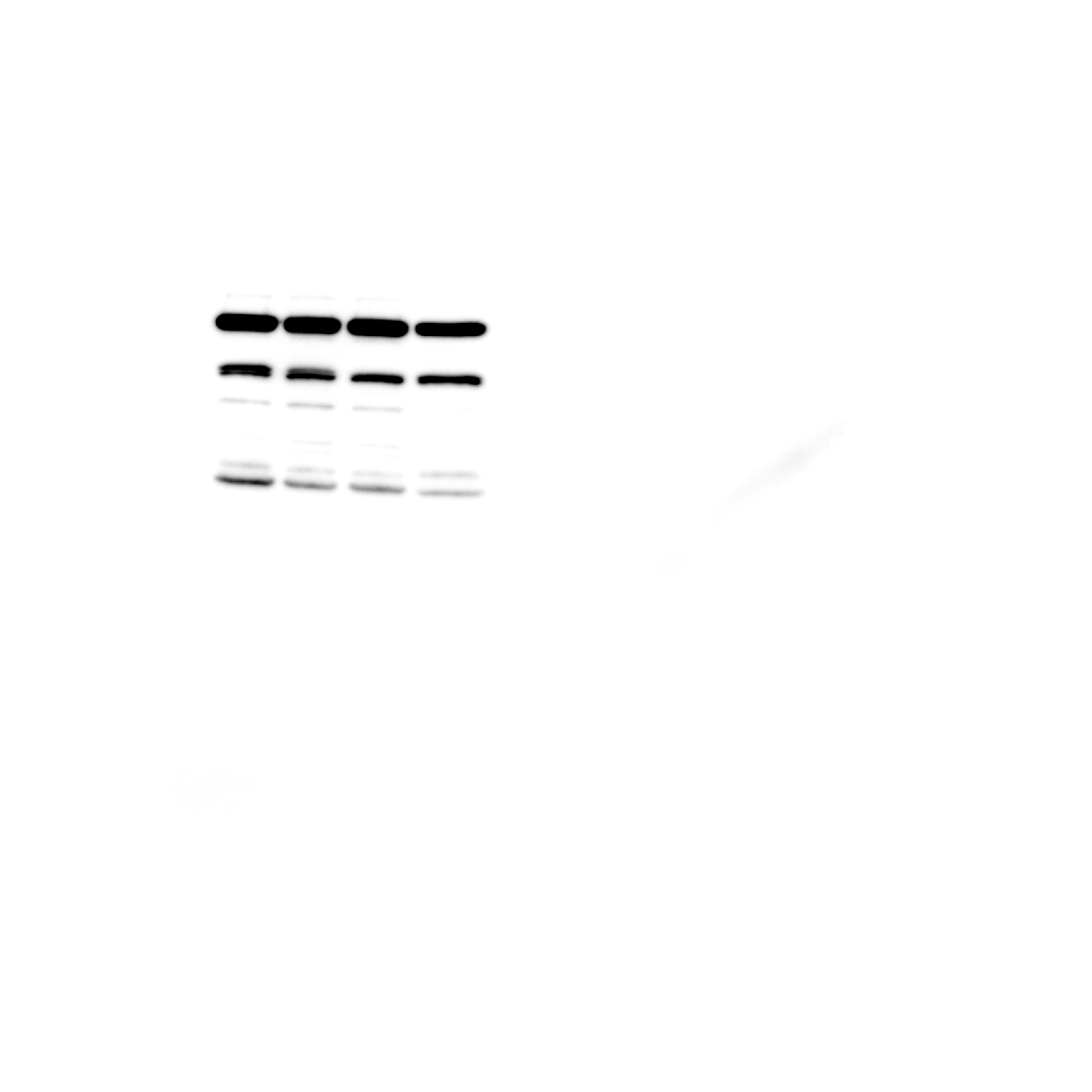

Supplement: Figure 8—figure supplement 1—source data 1. [file elife-69676-fig8-figsupp1-data1.zip › Figure8_figure_supplement1/PanelB/cfp 20210628_135605_Ch.tif]

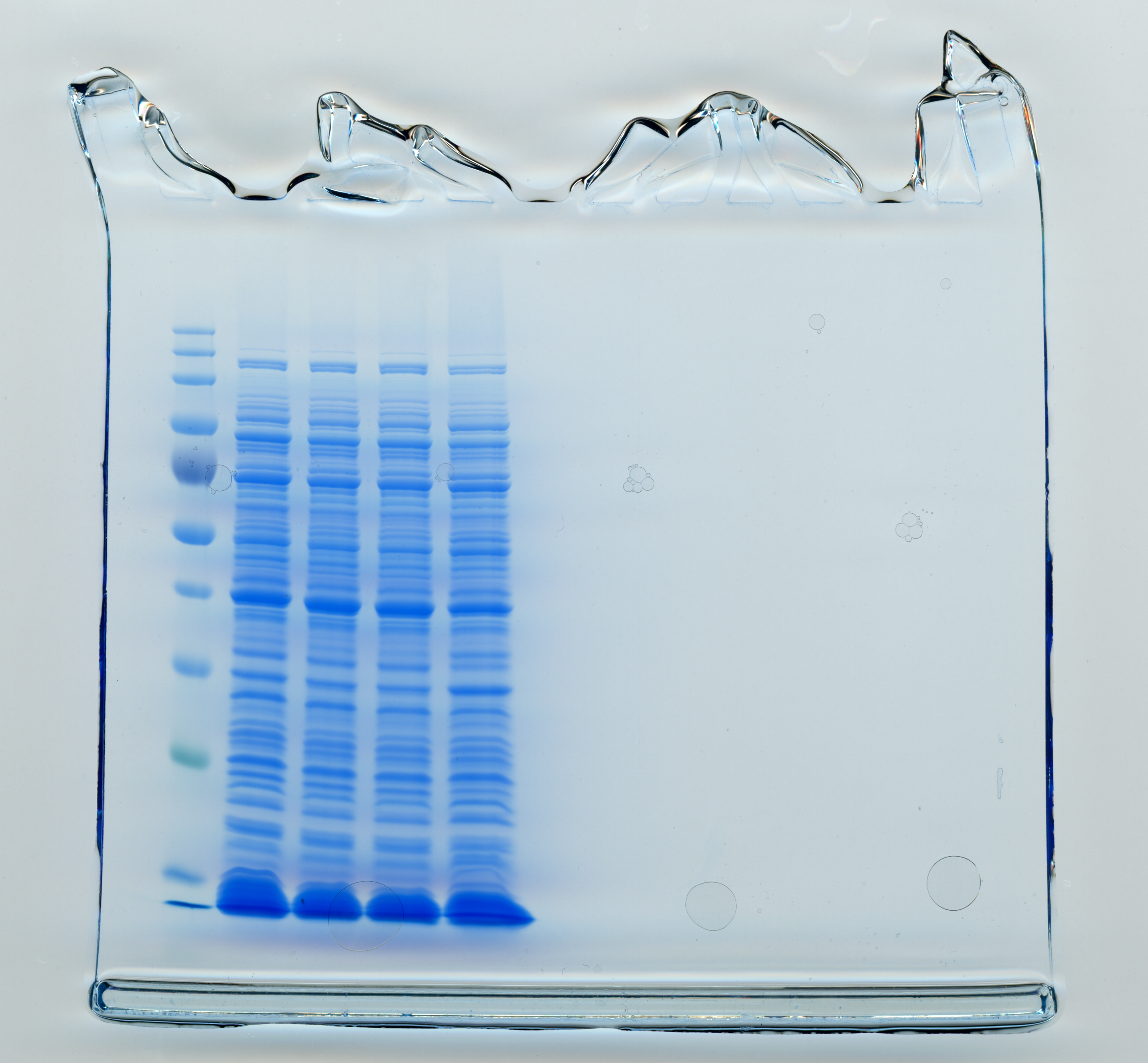

Supplement: Figure 8—figure supplement 1—source data 1. [file elife-69676-fig8-figsupp1-data1.zip › Figure8_figure_supplement1/PanelB/loading_WB_E102Acfp001.tif]

PYE + xylose

WT = ParB (WT)

7=Q58A

10=R60A

13=E102A

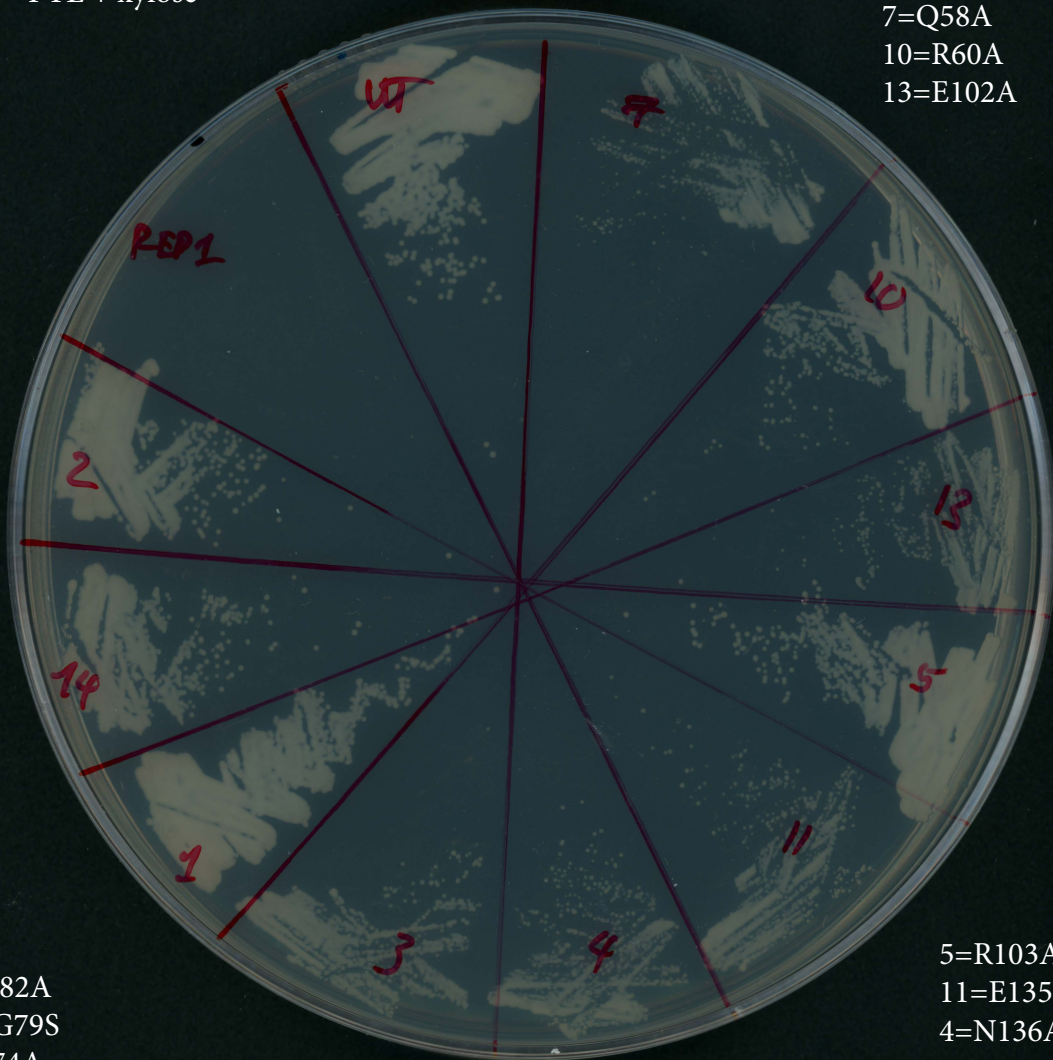

2=Q82A

14=G79S

1=S74A

3=R139A

5=R103A

11=E135A

4=N136A

PYE + glucose

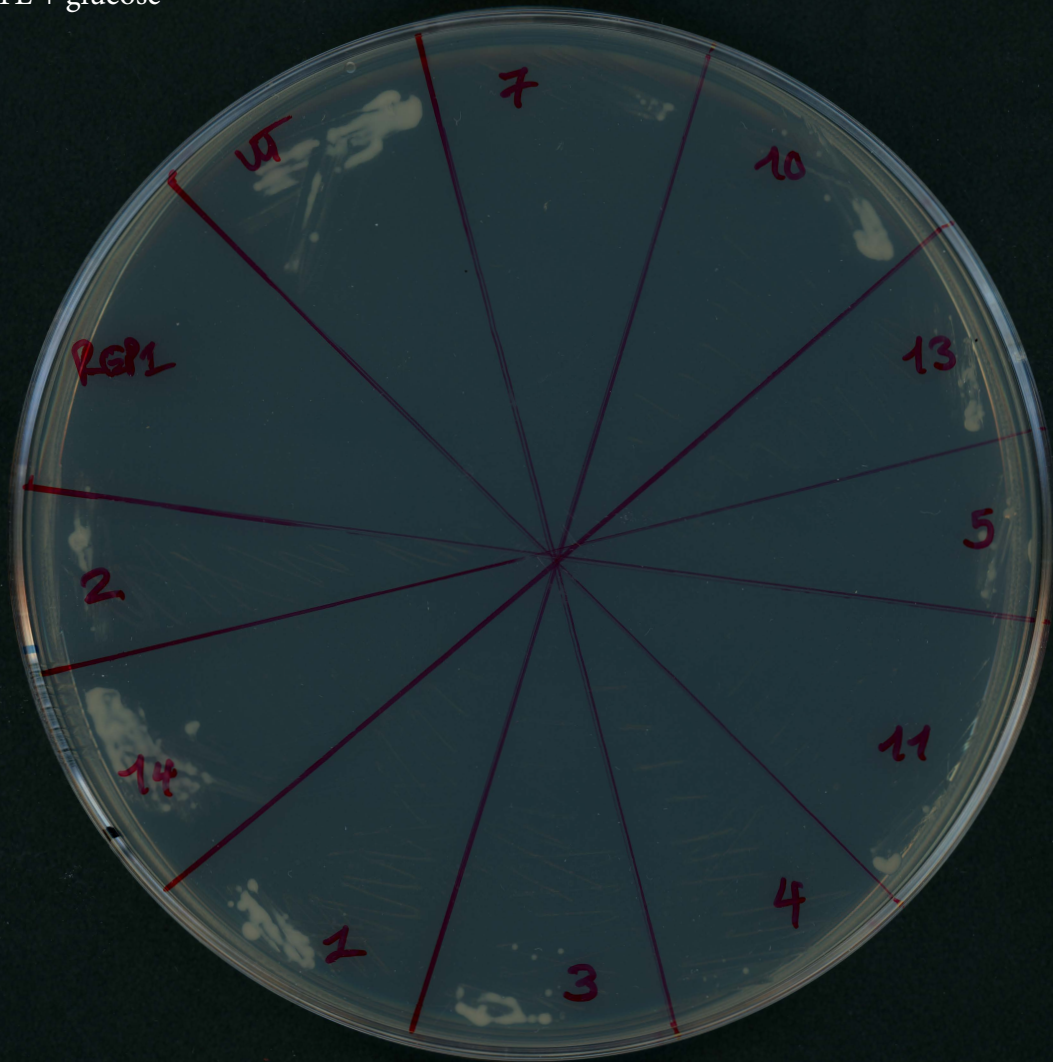

PYE + glucose + vanillate

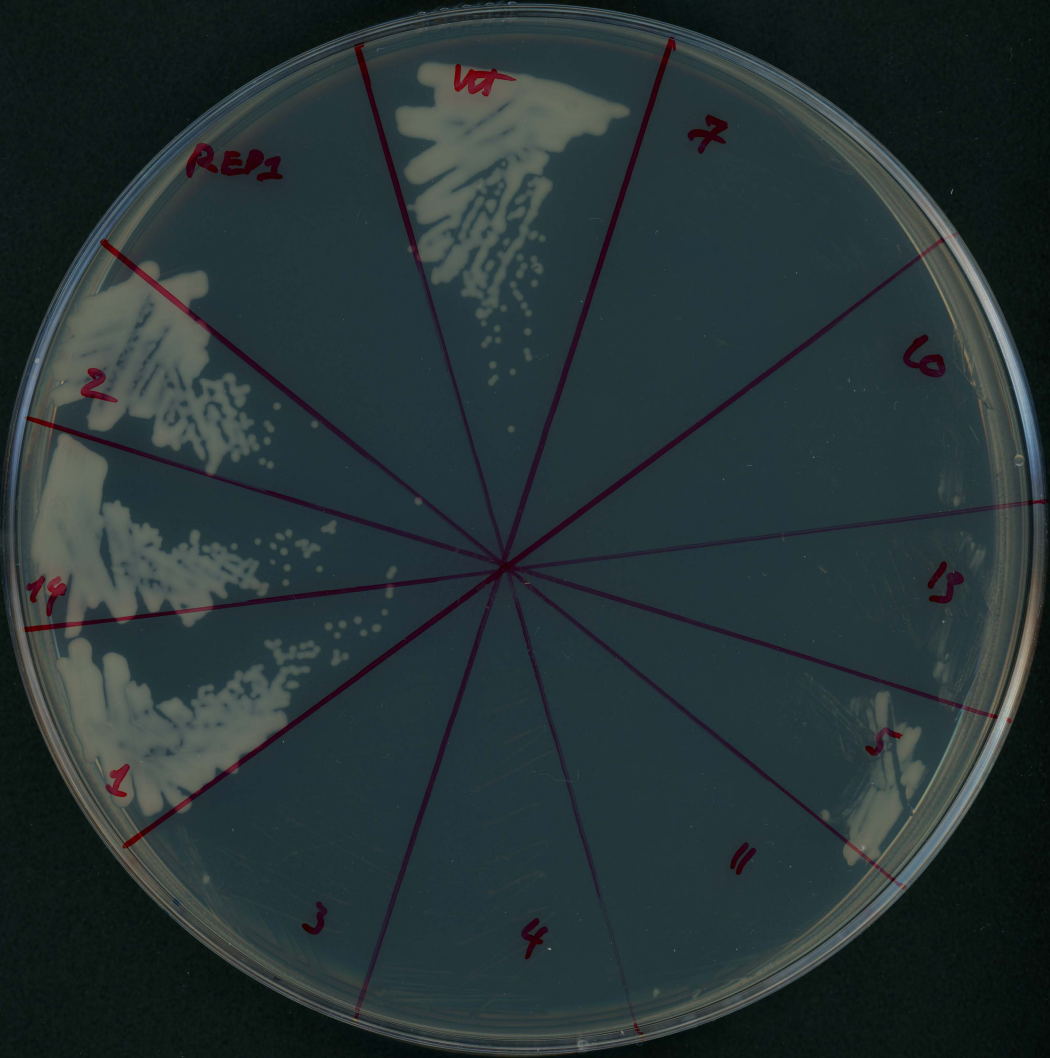

Supplement: Figure 8—figure supplement 2—source data 1. [file elife-69676-fig8-figsupp2-data1.zip › Figure8_figure_supplement2/Annotation.pdf]

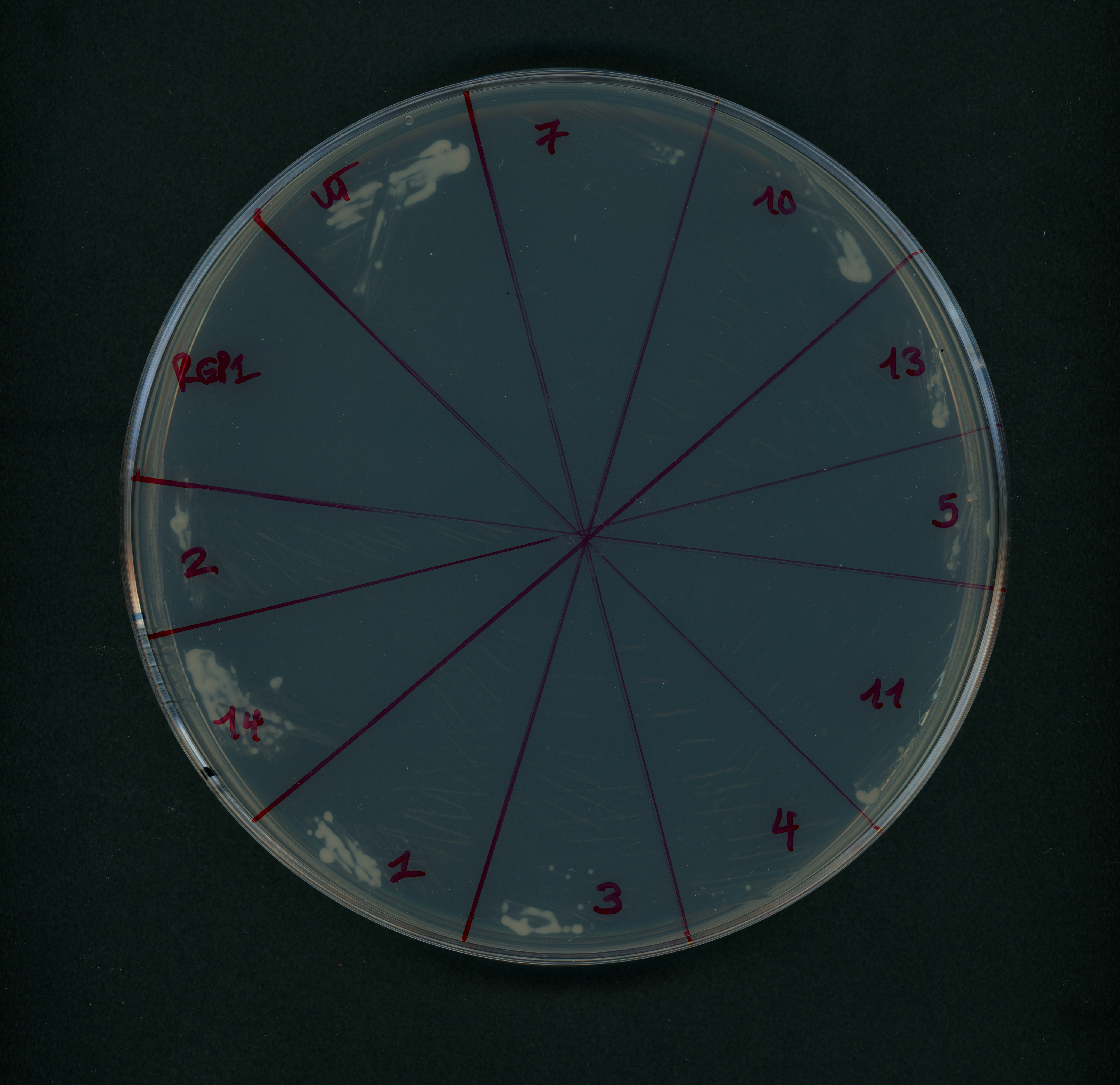

Supplement: Figure 8—figure supplement 2—source data 1. [file elife-69676-fig8-figsupp2-data1.zip › Figure8_figure_supplement2/Rep1_All_Glucose_MT148bg003.tif]

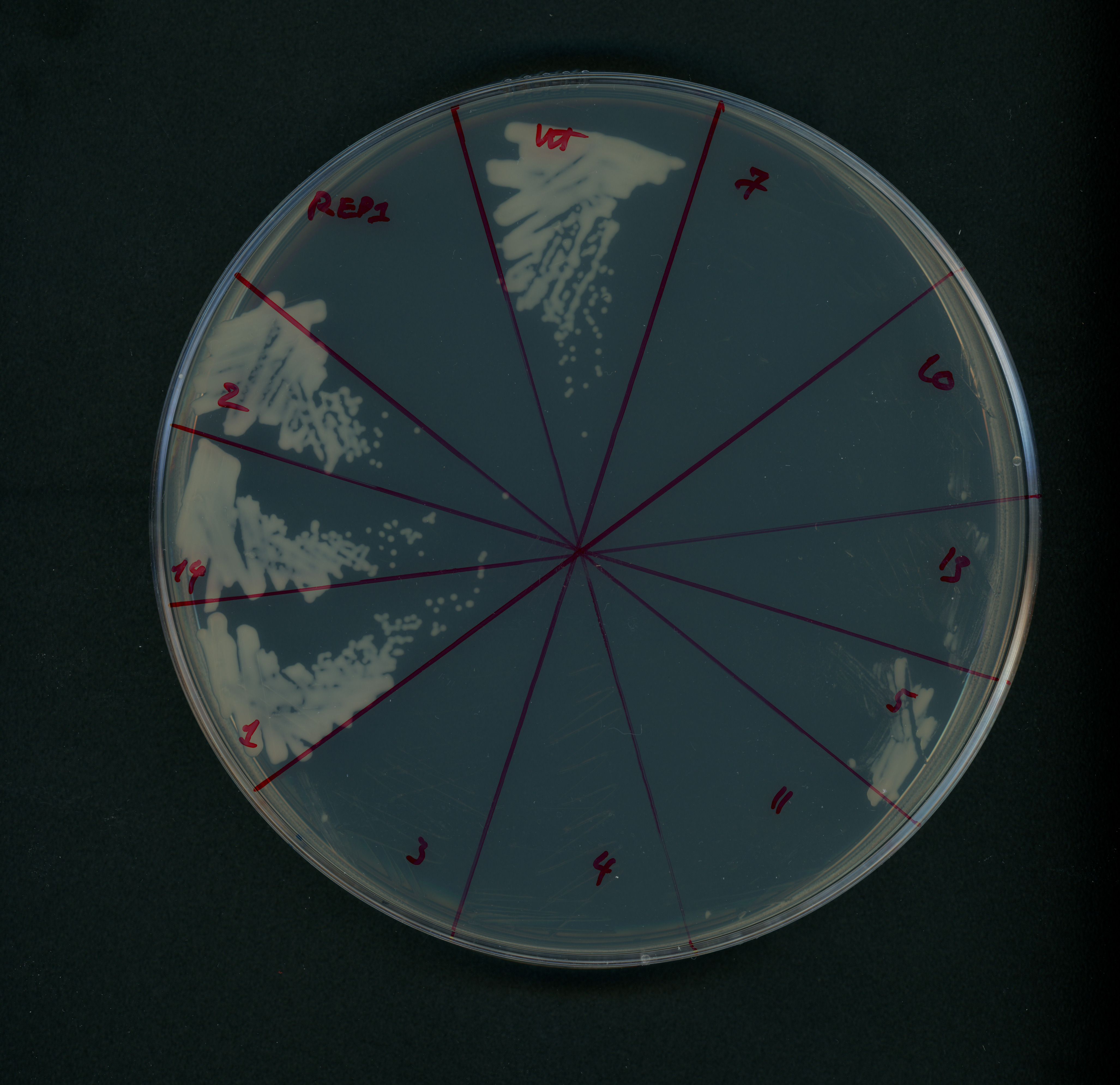

Supplement: Figure 8—figure supplement 2—source data 1. [file elife-69676-fig8-figsupp2-data1.zip › Figure8_figure_supplement2/Rep1_All_GlucoseVan_MT148bg003.tif]

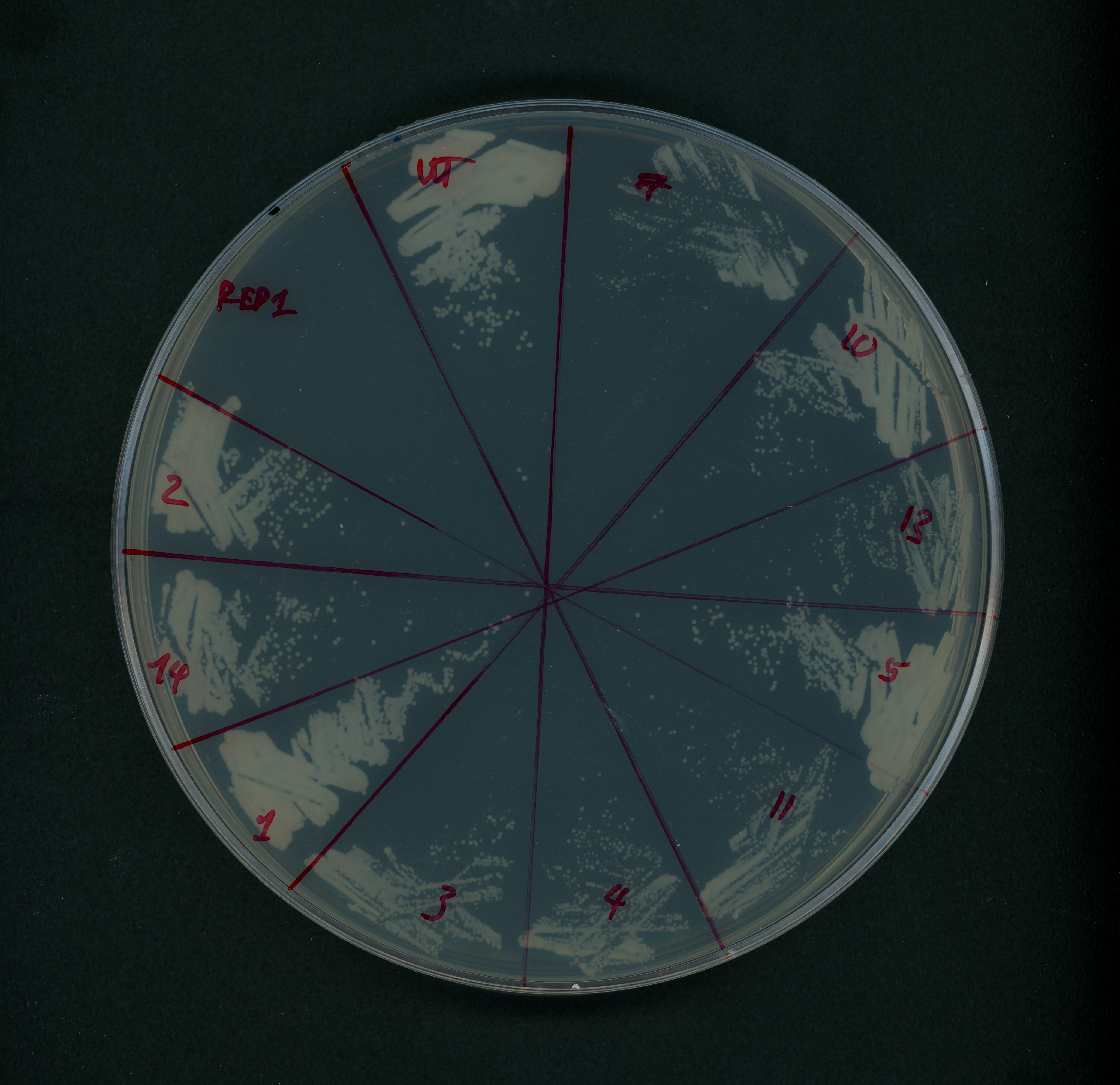

Supplement: Figure 8—figure supplement 2—source data 1. [file elife-69676-fig8-figsupp2-data1.zip › Figure8_figure_supplement2/Rep1_All_Xylose_MT148bg003.tif]

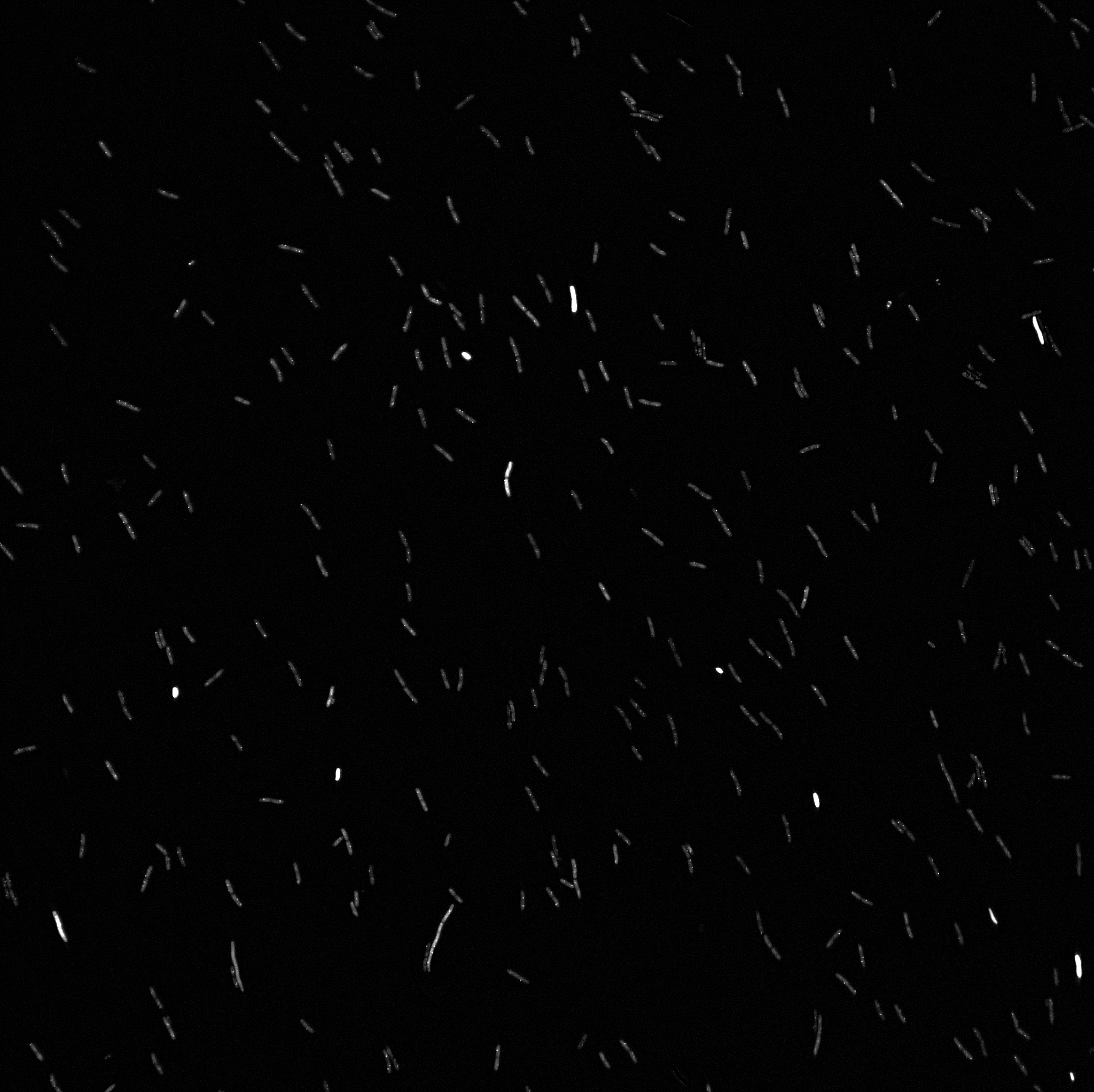

Supplement: Figure 8—figure supplement 3—source data 1. [file elife-69676-fig8-figsupp3-data1.zip › Figure8_figuresupplement3/Processed images/3079_wt/3079_0.25mMIPTG_435nm_1s_004c1.tif]

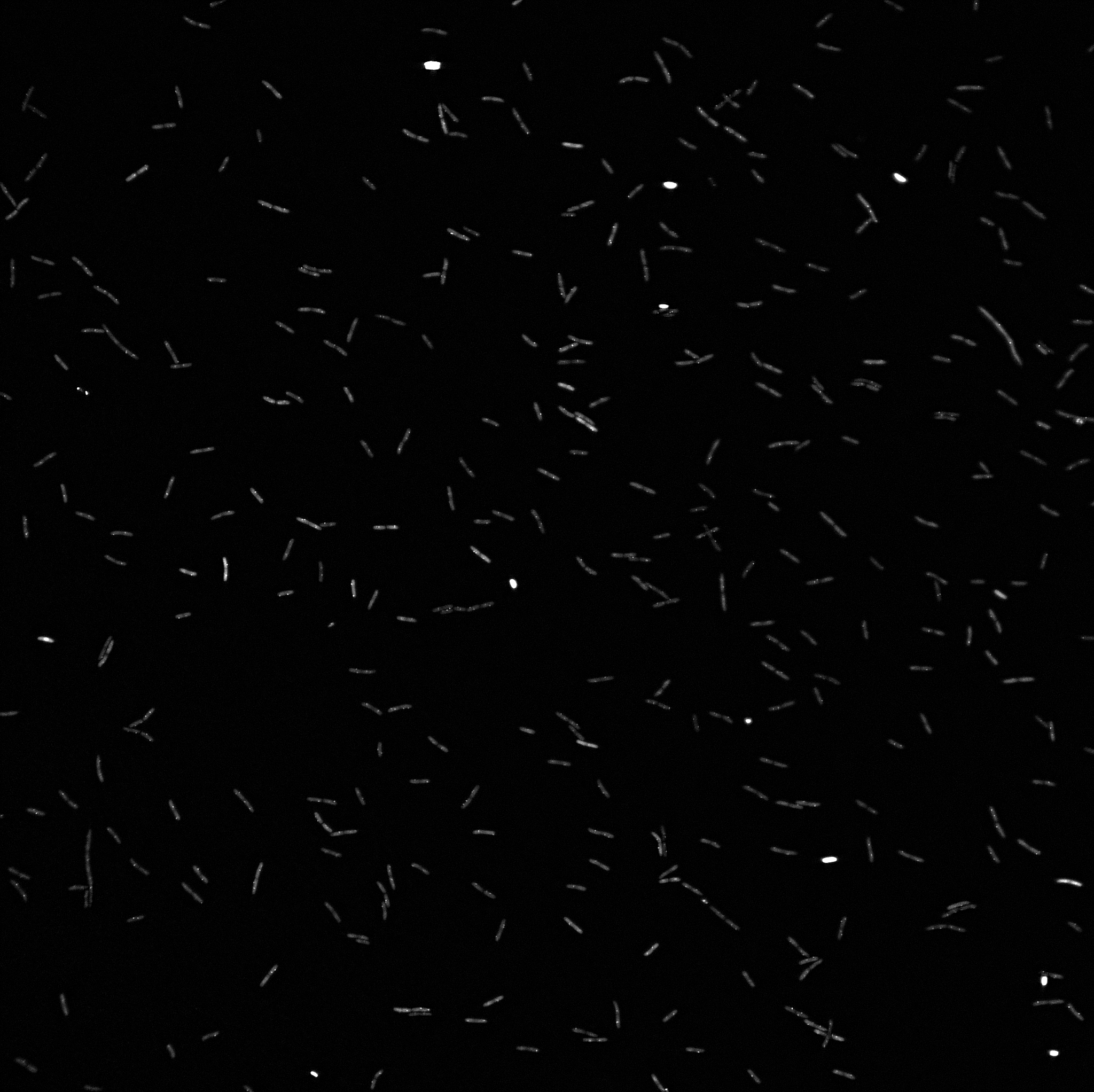

Supplement: Figure 8—figure supplement 3—source data 1. [file elife-69676-fig8-figsupp3-data1.zip › Figure8_figuresupplement3/Processed images/3079_wt/3079_0.25mMIPTG_435nm_1s_015c1.tif]

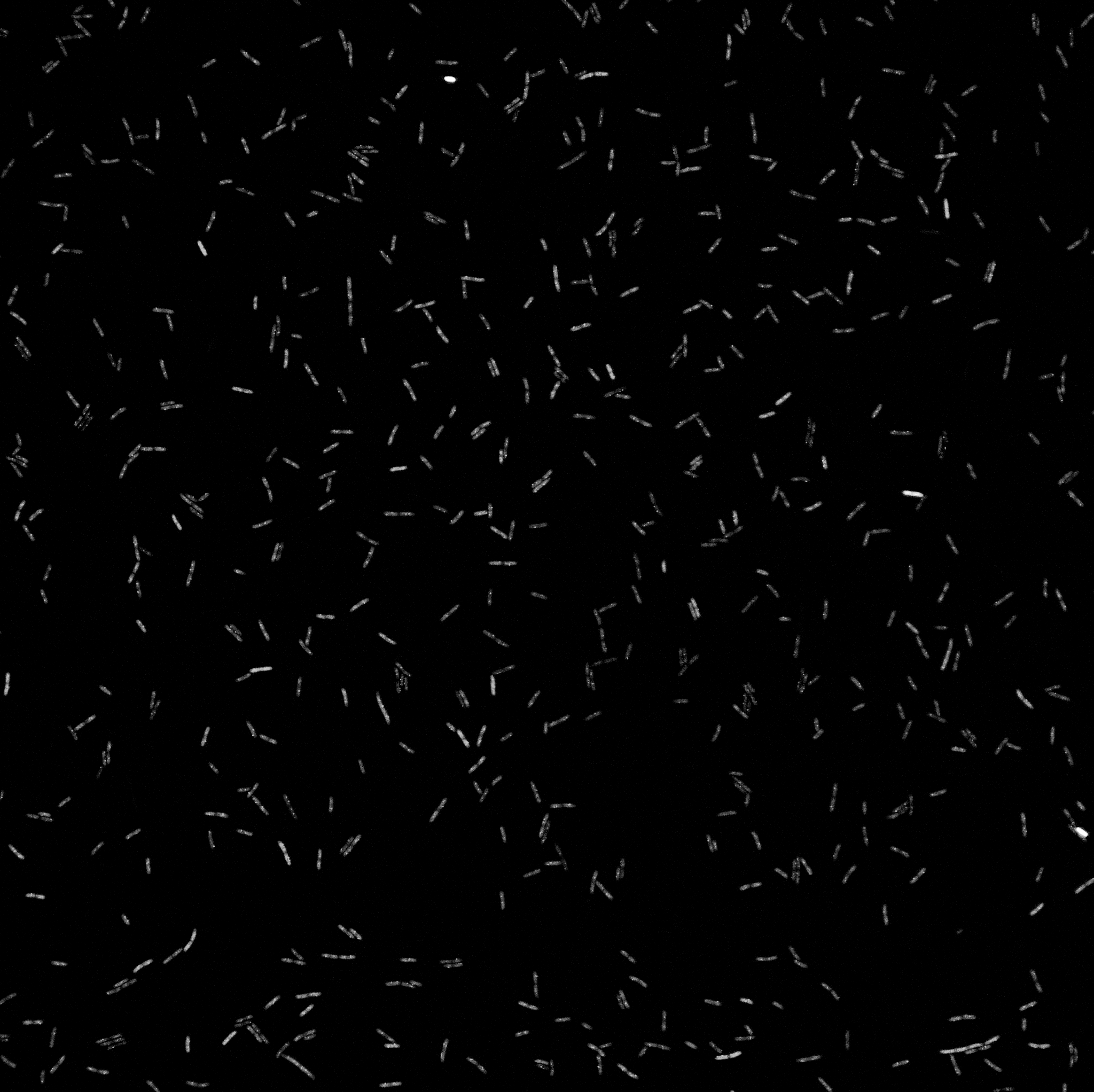

Supplement: Figure 8—figure supplement 3—source data 1. [file elife-69676-fig8-figsupp3-data1.zip › Figure8_figuresupplement3/Processed images/3079_wt/3079_435nm_1s_0.25mMIPTG_c1.tif]

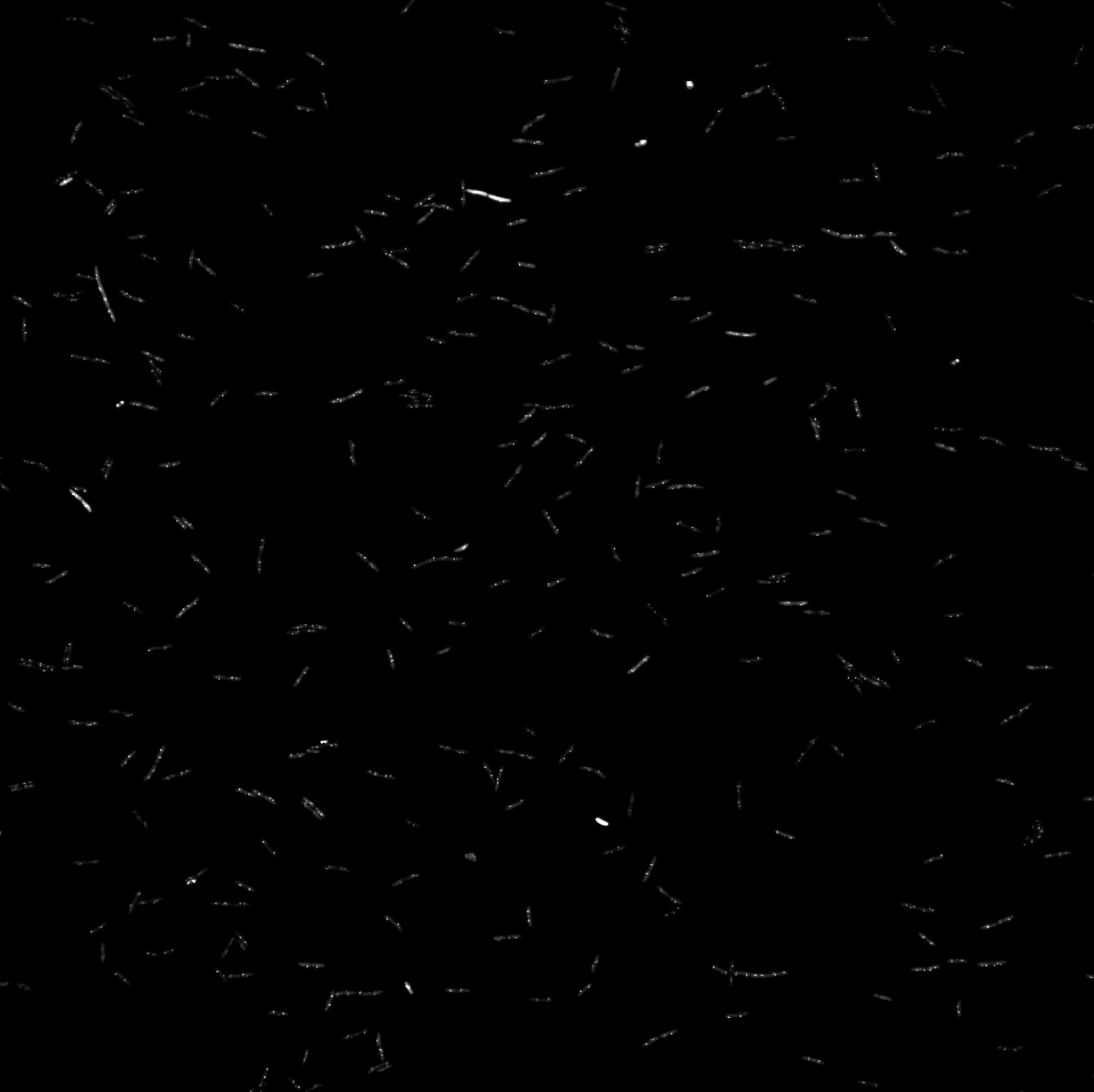

Supplement: Figure 8—figure supplement 3—source data 1. [file elife-69676-fig8-figsupp3-data1.zip › Figure8_figuresupplement3/Processed images/3080_mutant/3080_0.25mMIPTG_435nm_1s_007c1.tif]

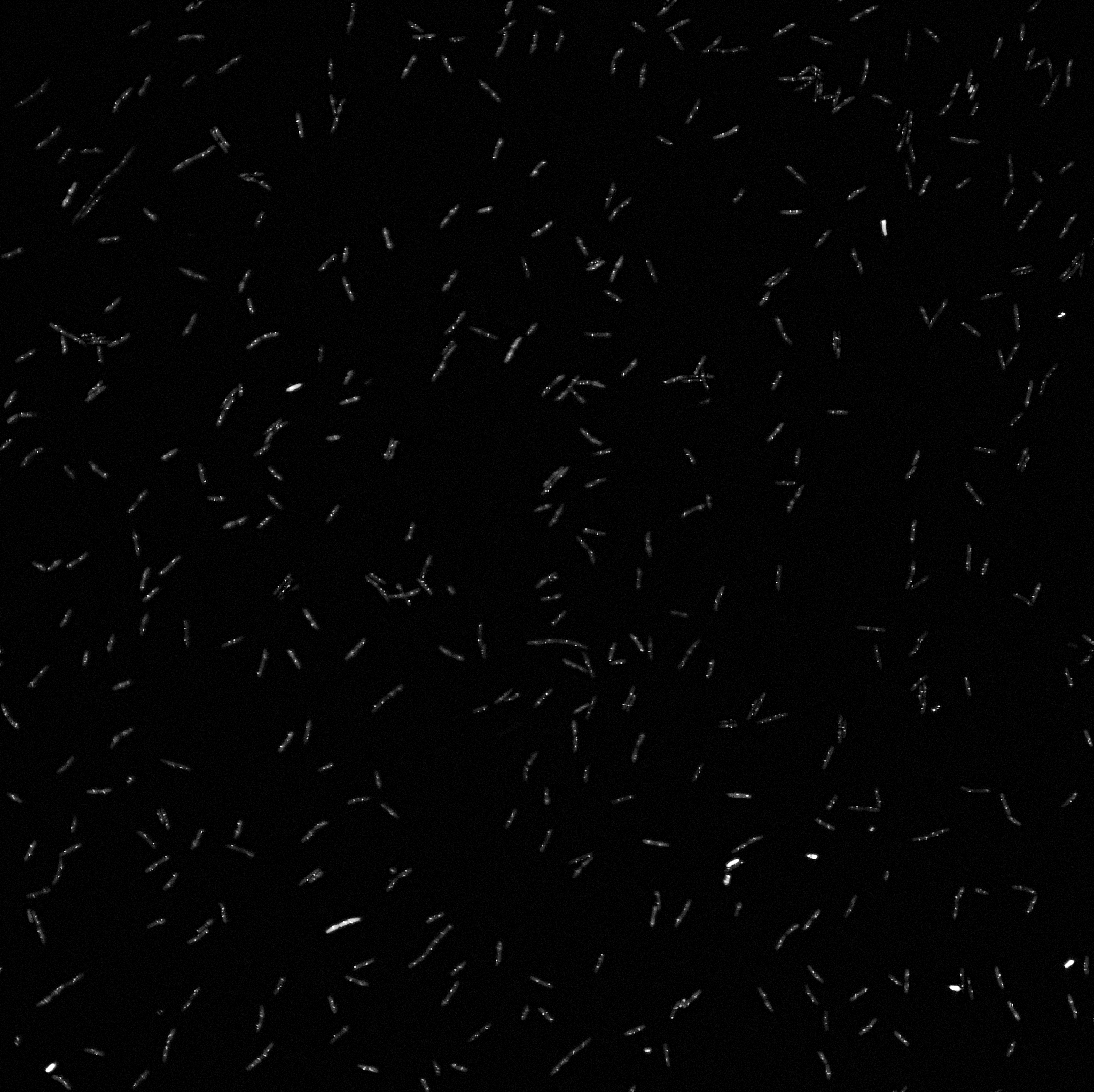

Supplement: Figure 8—figure supplement 3—source data 1. [file elife-69676-fig8-figsupp3-data1.zip › Figure8_figuresupplement3/Processed images/3080_mutant/3080_0.25mMIPTG_435nm_1s_019c1.tif]

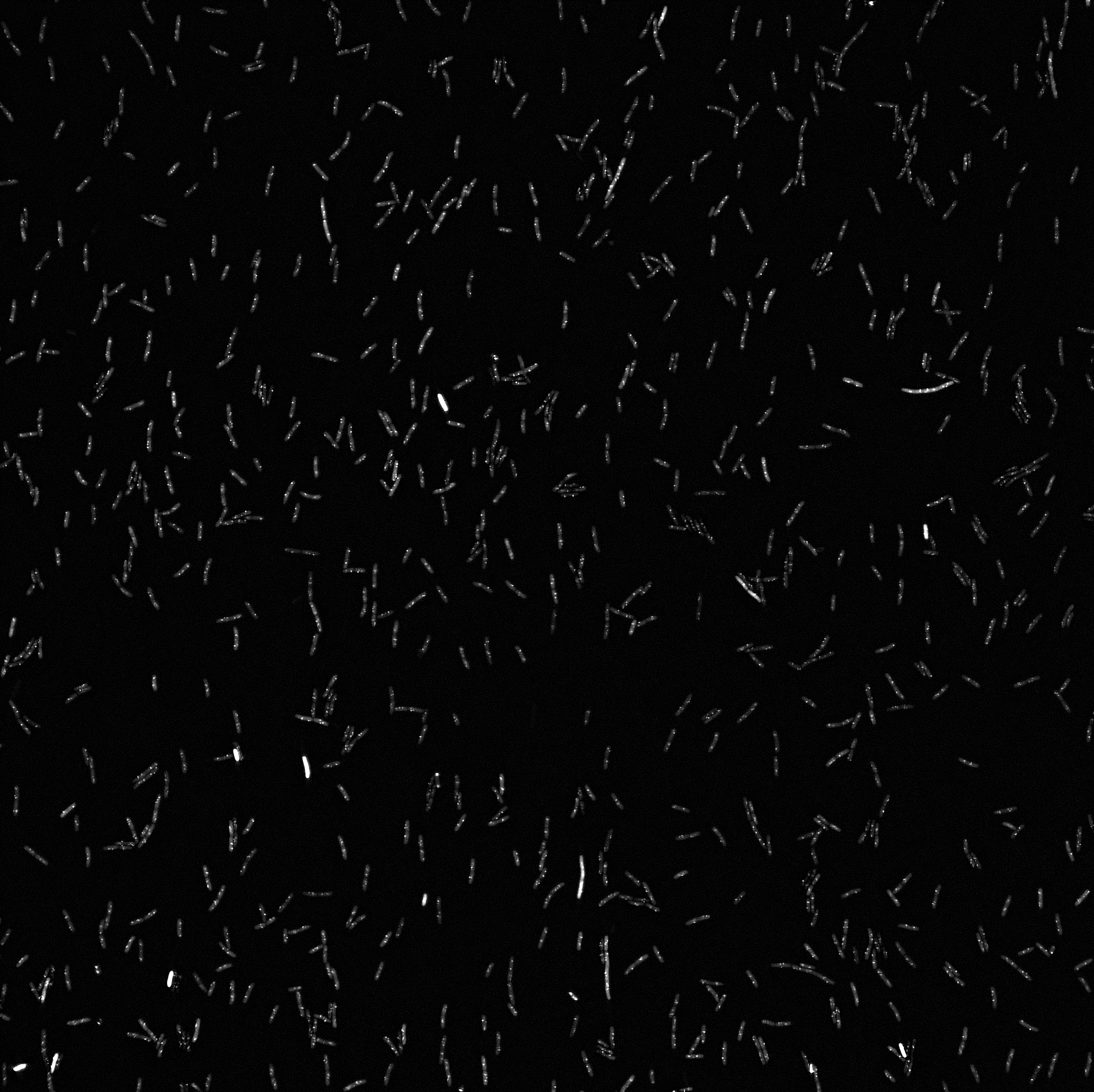

Supplement: Figure 8—figure supplement 3—source data 1. [file elife-69676-fig8-figsupp3-data1.zip › Figure8_figuresupplement3/Processed images/3080_mutant/3080_435nm_1s_0.25mMIPTG_002c1.tif]
