# Supplementary material for: A CTP-dependent gating mechanism enables ParB spreading on DNA
Source: eLife. 2021 Aug 16;10:e69676. doi: 10.7554/eLife.69676 (PMC8367383; doi:10.7554/eLife.69676)
Supplement: Figure 5—figure supplement 3—source data 1. [file elife-69676-fig5-figsupp3-data1.zip › Figure5_figure_supplement3/PanelB/Annotation.pdf]

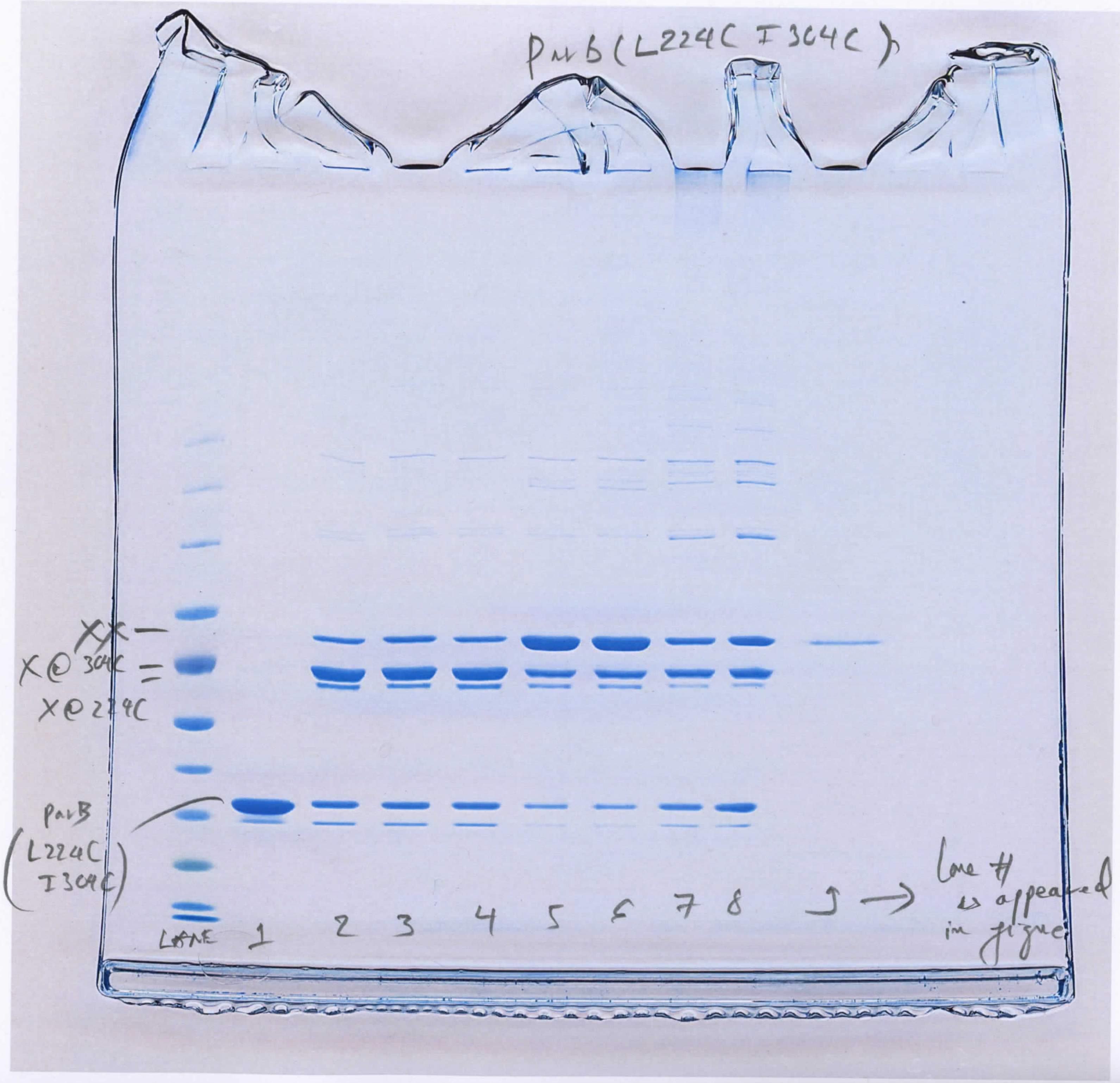

ParB (L224C I304C)
XX —
X@304C =
X@224C
ParB
(L224C
I304C)
LANE
1
2
3
4
5
6
7
8
Lane # as appeared in figure

5/52 B
