# Supplementary material for: A CTP-dependent gating mechanism enables ParB spreading on DNA
Source: eLife. 2021 Aug 16;10:e69676. doi: 10.7554/eLife.69676 (PMC8367383; doi:10.7554/eLife.69676)

Figure 5-
figure supp 3
(B)

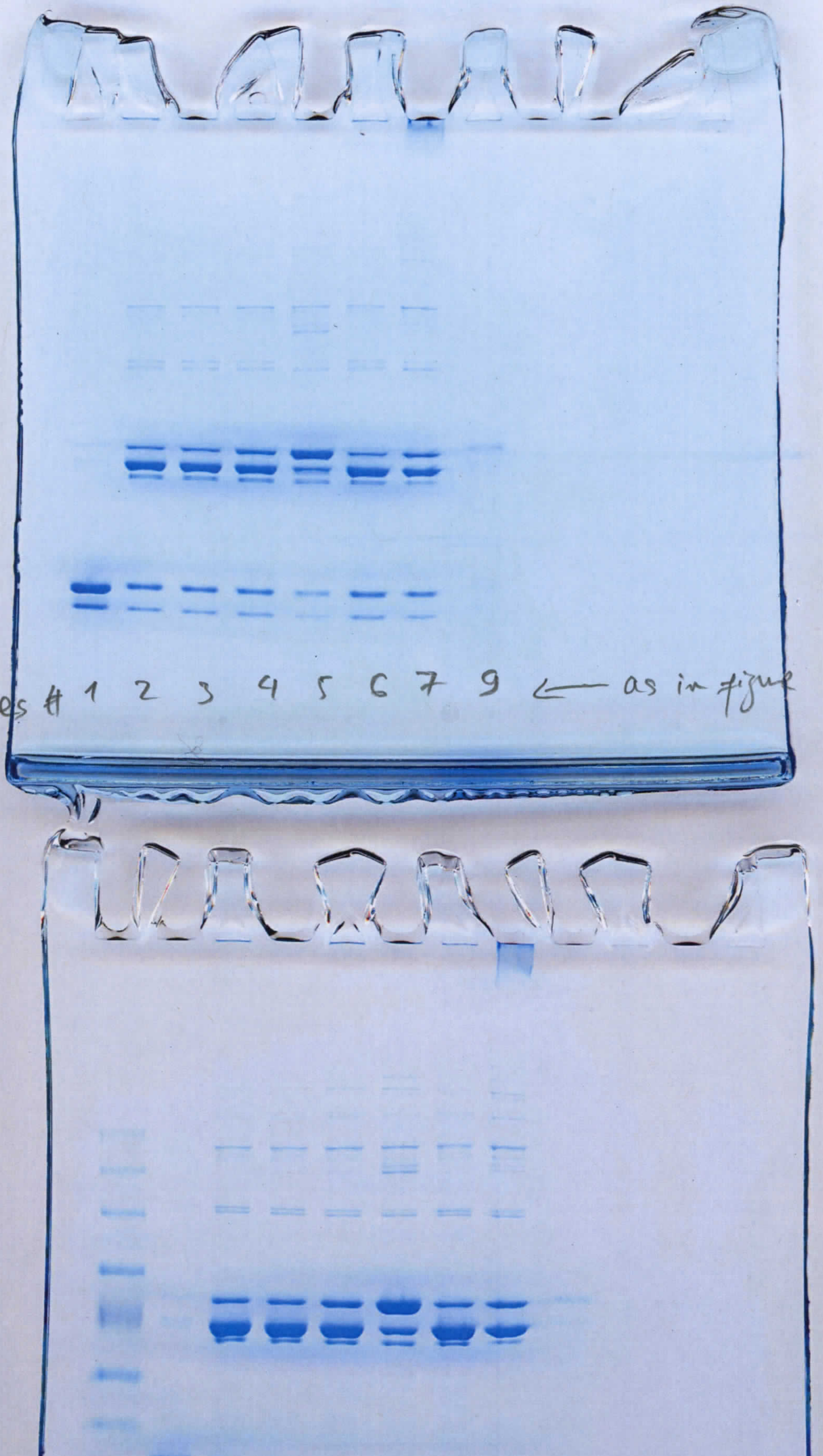

Supplement: Figure 5—figure supplement 3—source data 1. [file elife-69676-fig5-figsupp3-data1.zip › Figure5_figure_supplement3/PanelB/other_replicates/Annotation.pdf]
