# Supplementary material for: A CTP-dependent gating mechanism enables ParB spreading on DNA
Source: eLife. 2021 Aug 16;10:e69676. doi: 10.7554/eLife.69676 (PMC8367383; doi:10.7554/eLife.69676)
Supplement: Figure 6—figure supplement 1—source data 1. [file elife-69676-fig6-figsupp1-data1.zip › Figure6_figure_supplement1/Annotation.pdf]

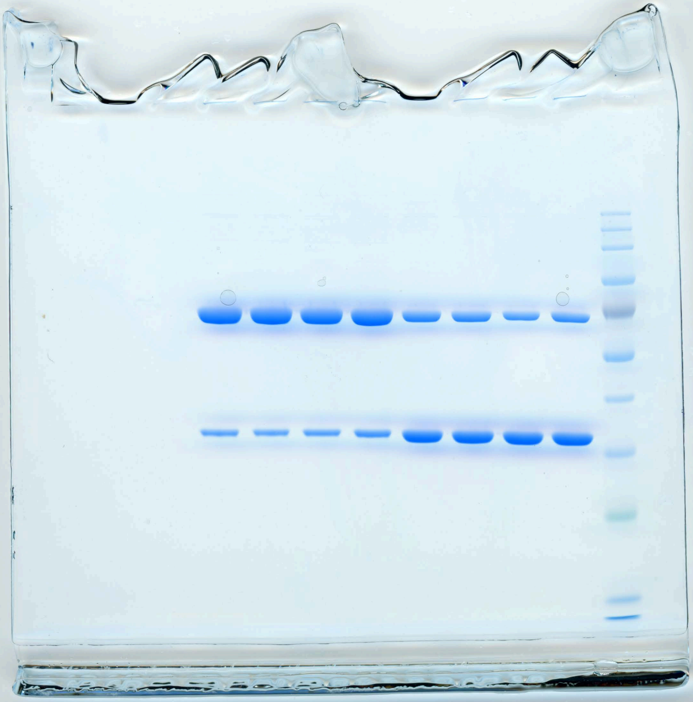
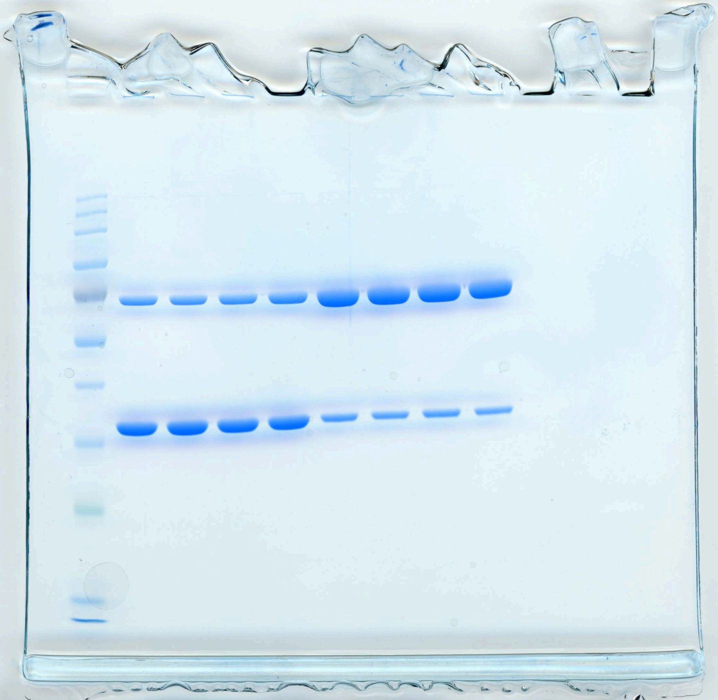

ParB (WT)

lanes# 1 to 8 from ladder, same as appeared in figure

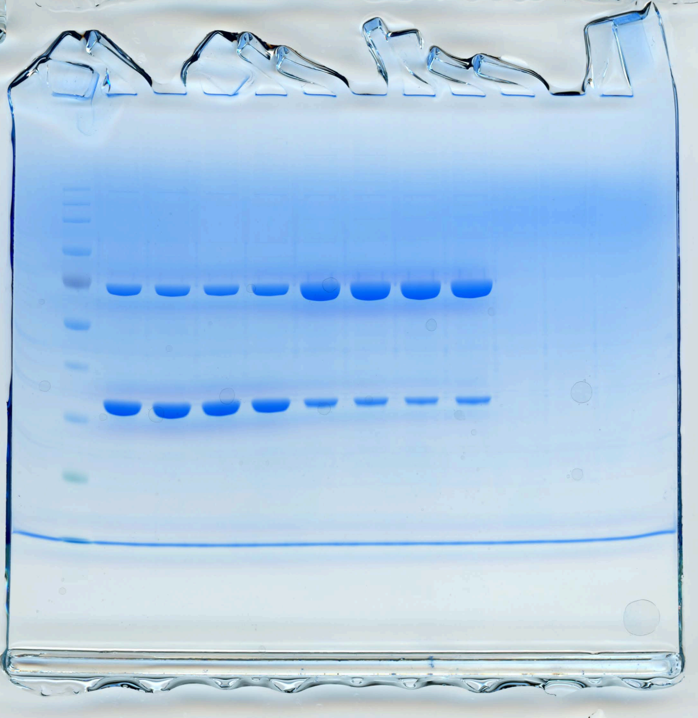

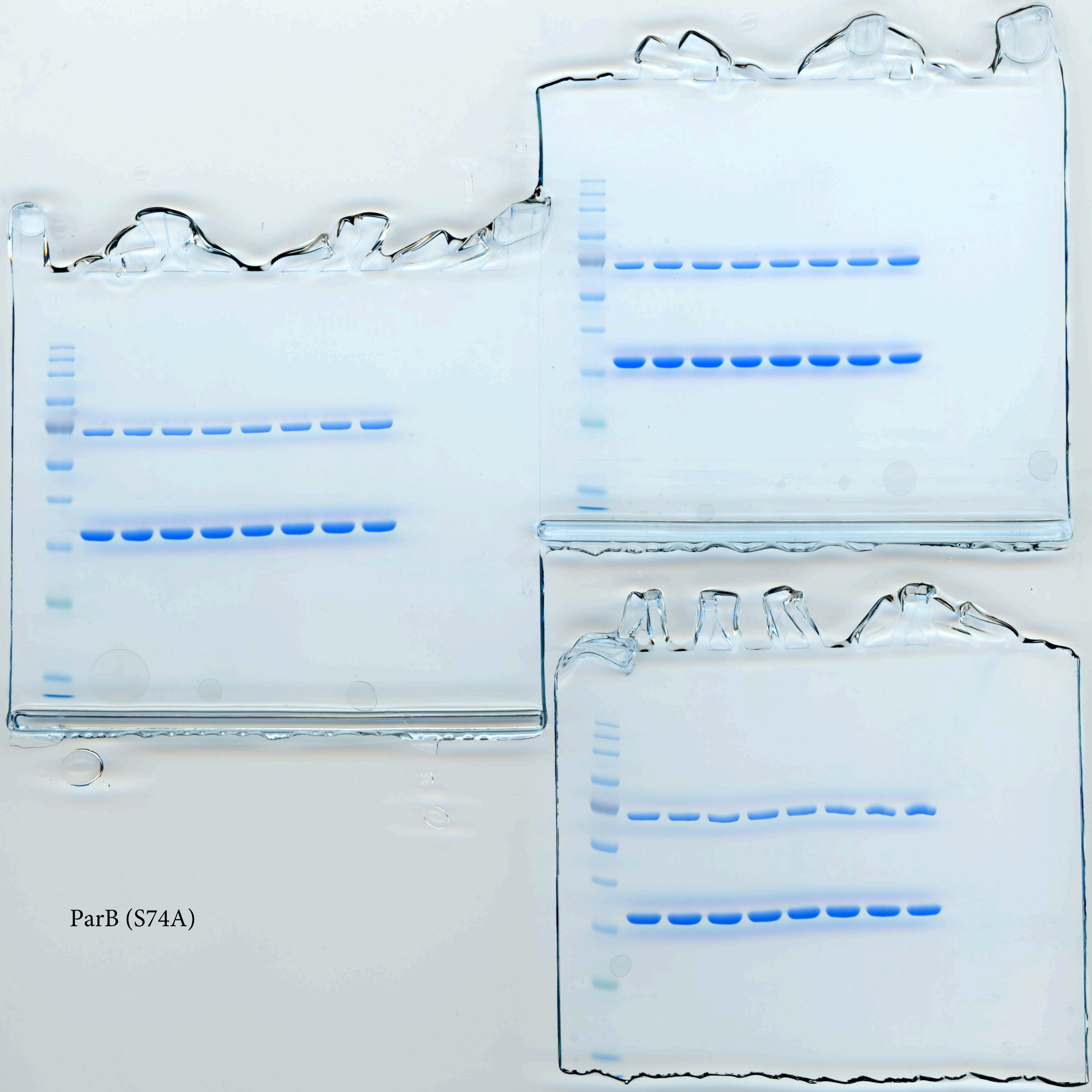

ParB (S74A)

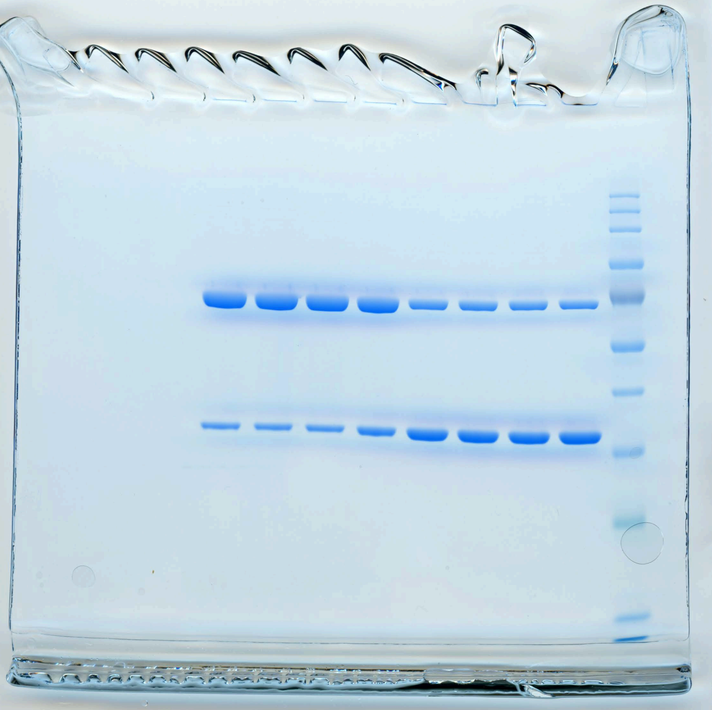

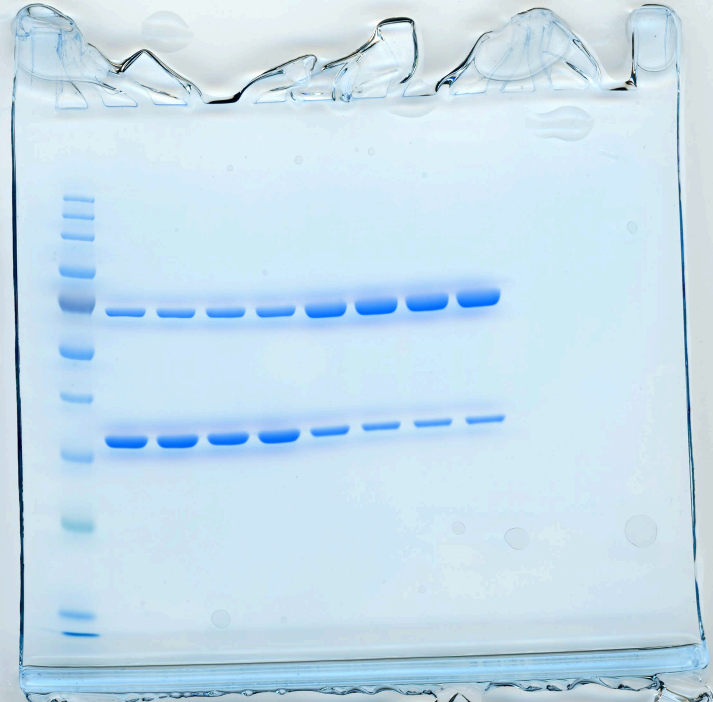

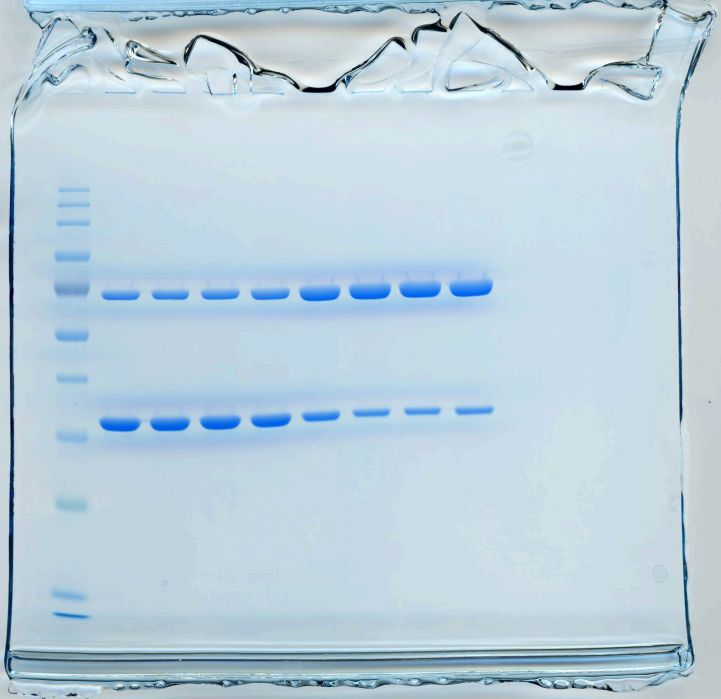

ParB (Q82A)

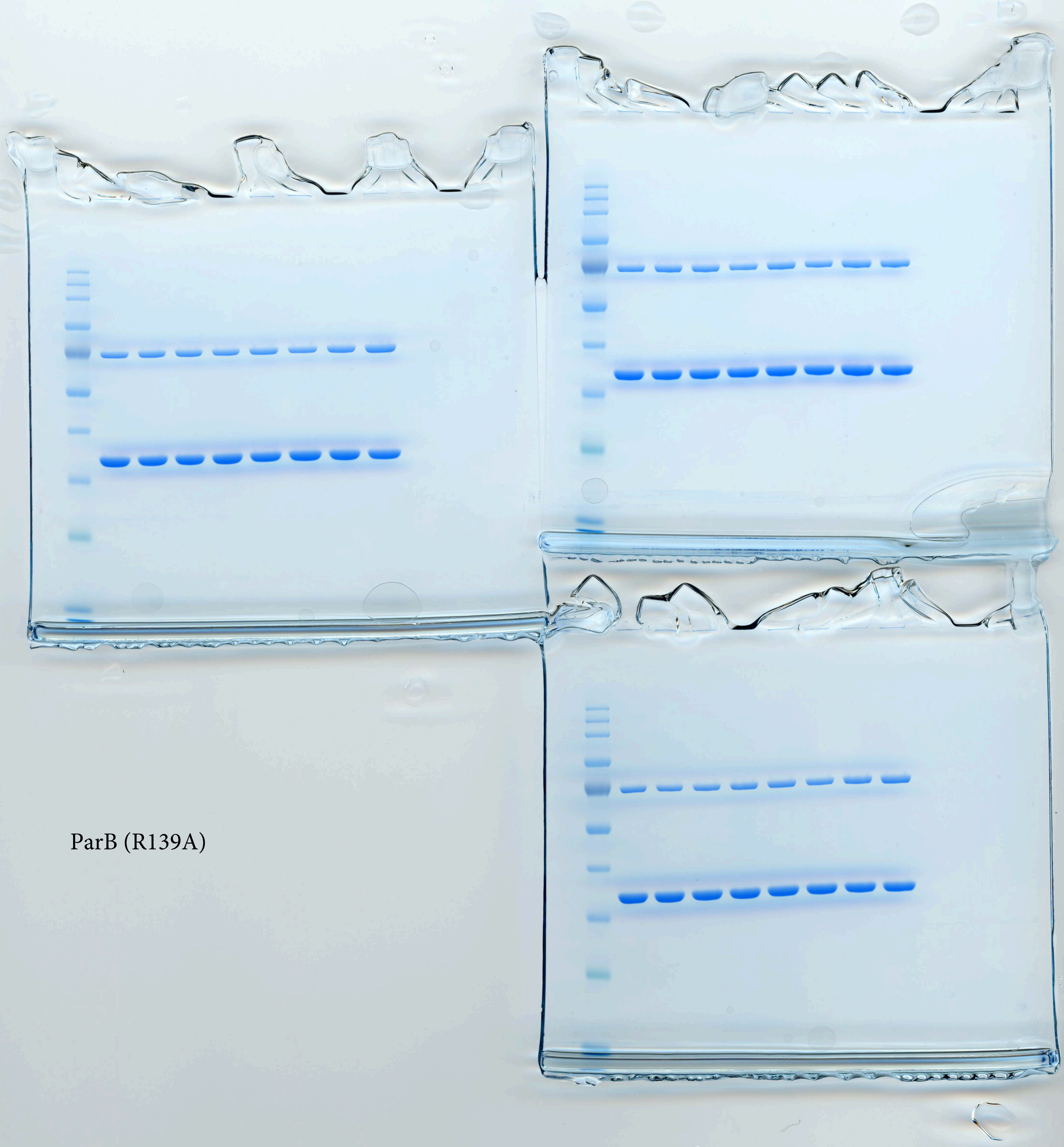

ParB (R139A)

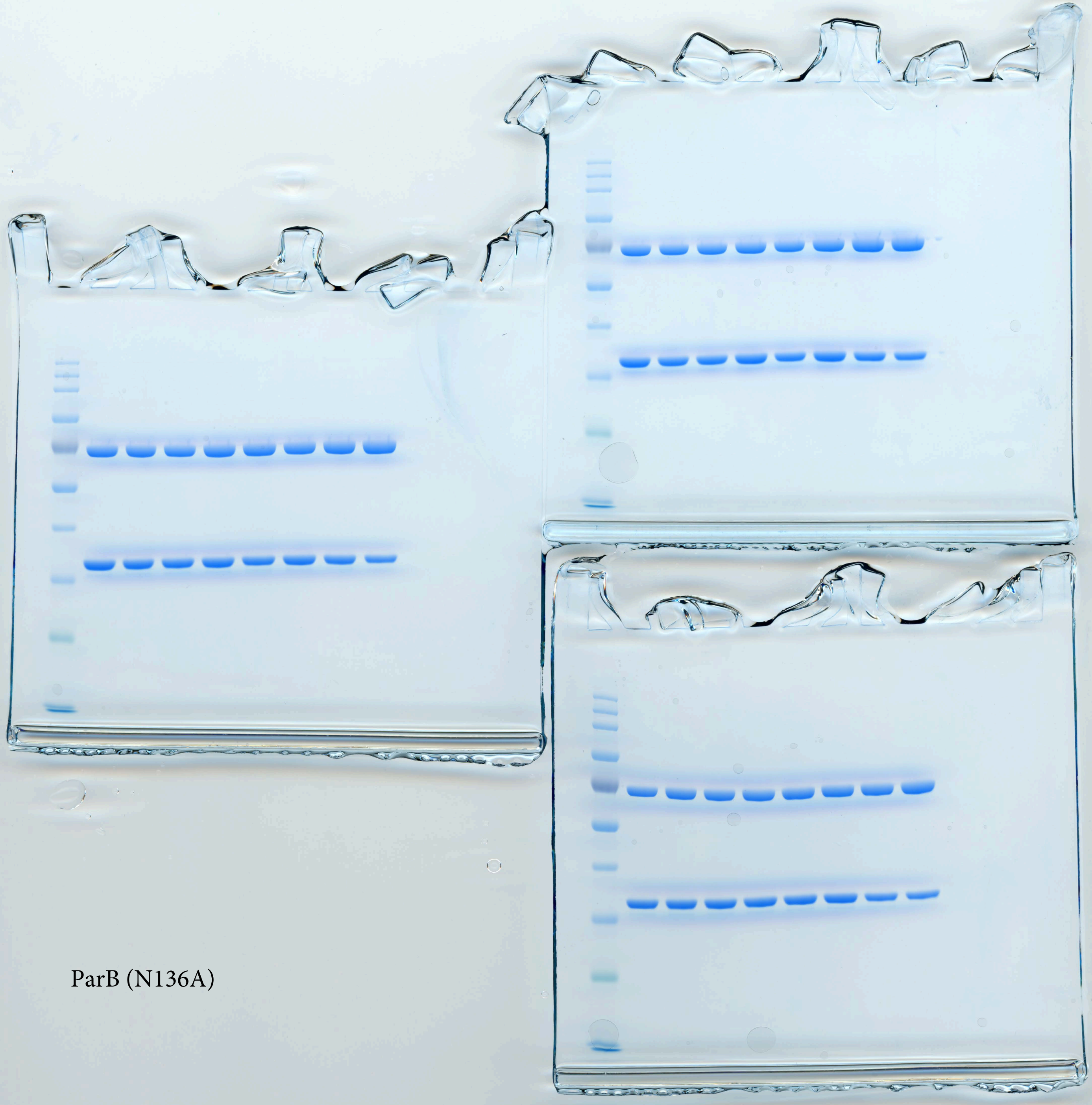

ParB (N136A)

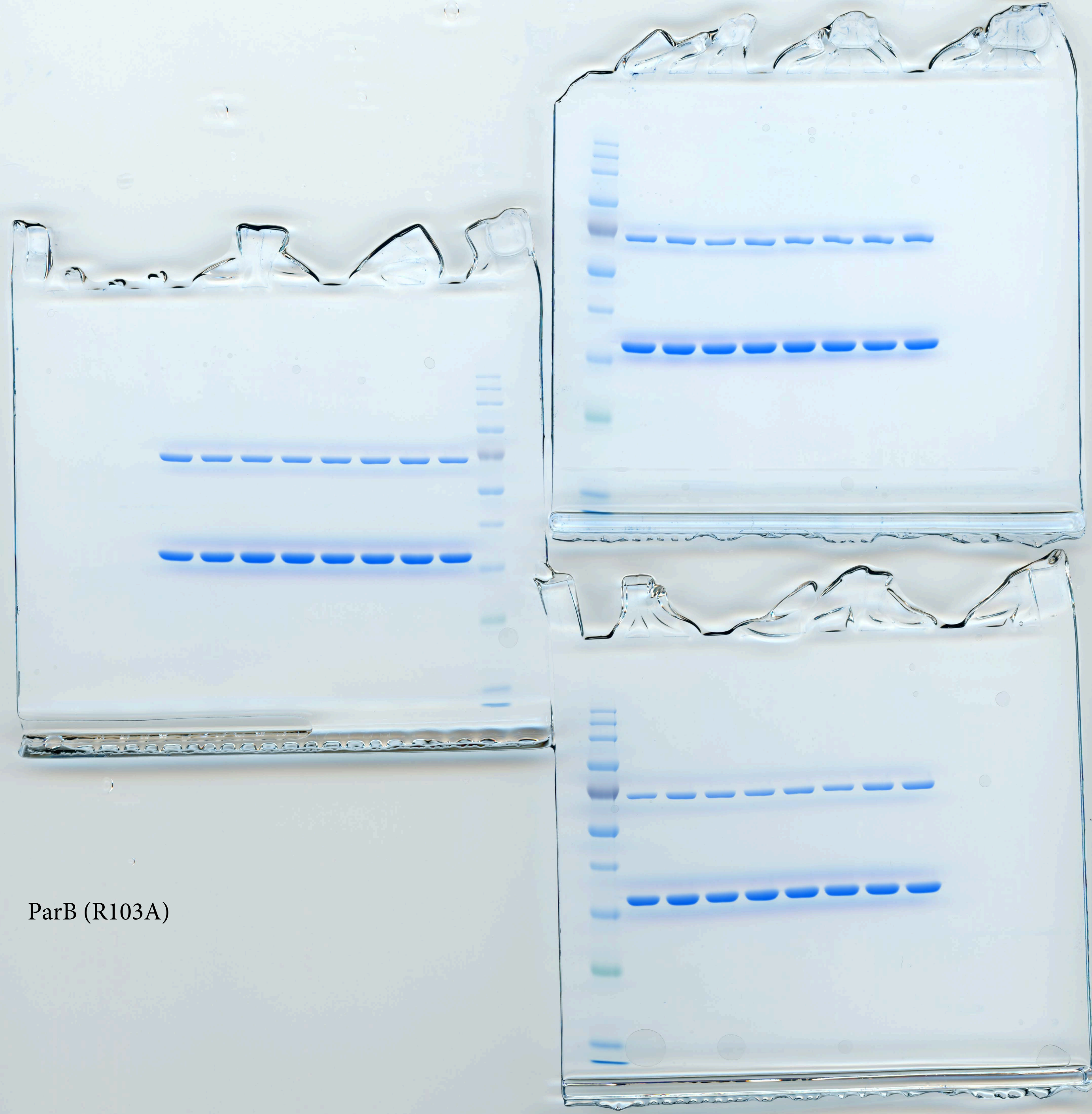

ParB (R103A)

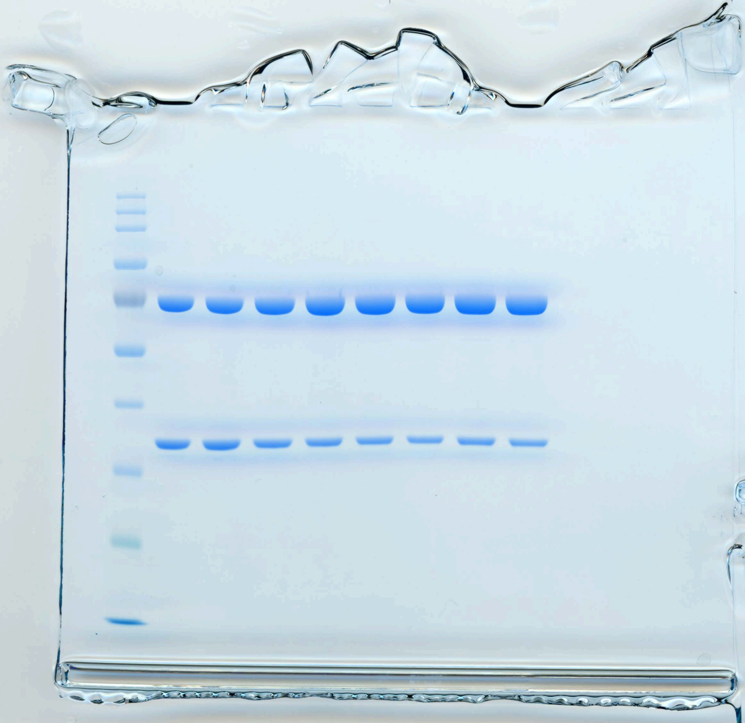

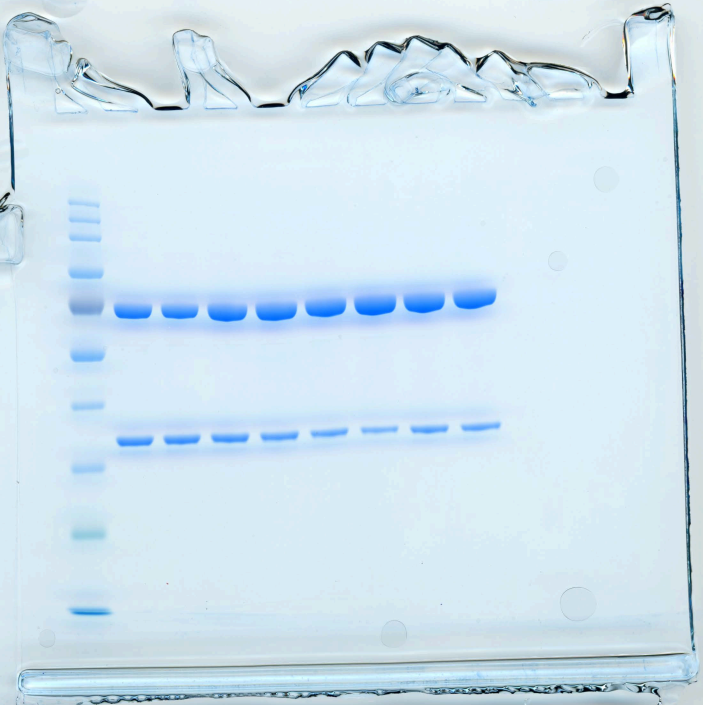

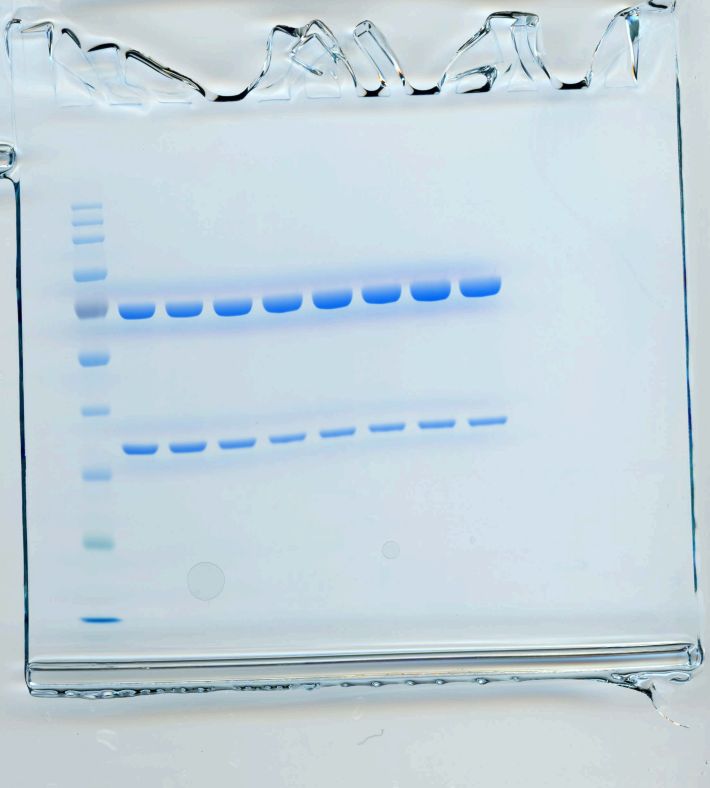

ParB(Q58A)

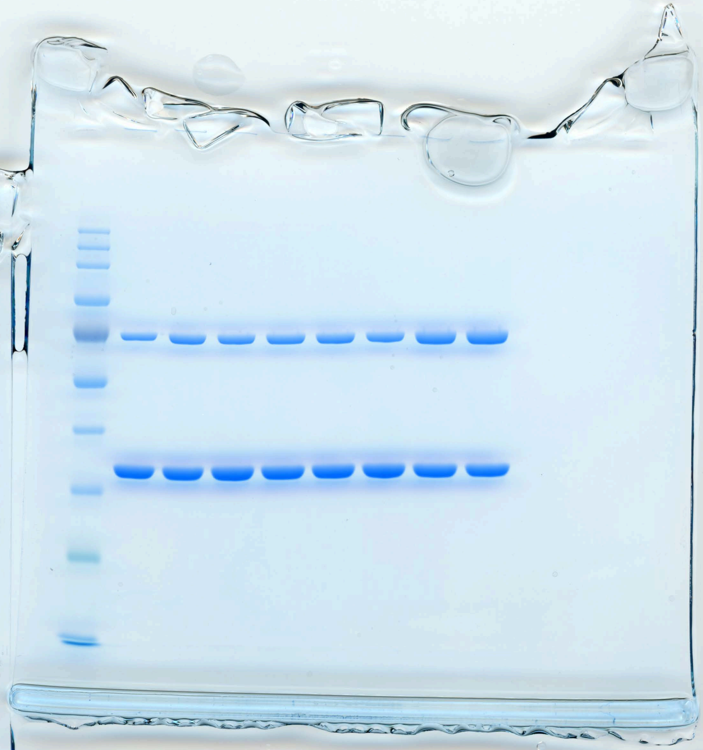

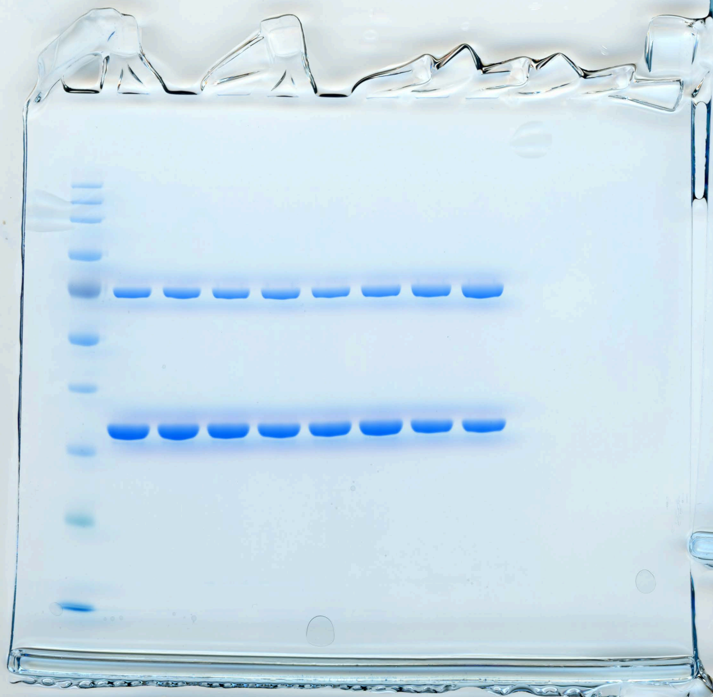

ParB (R60A)

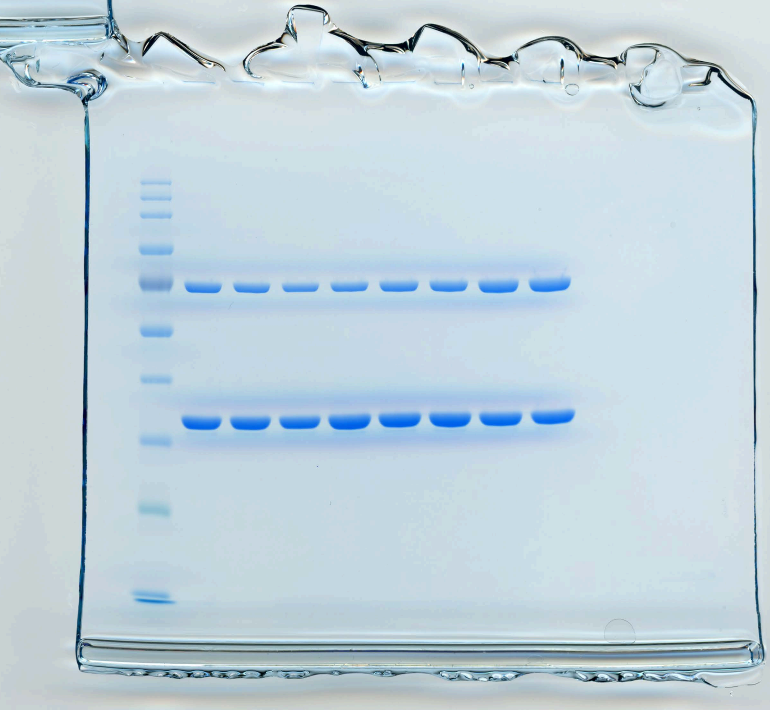

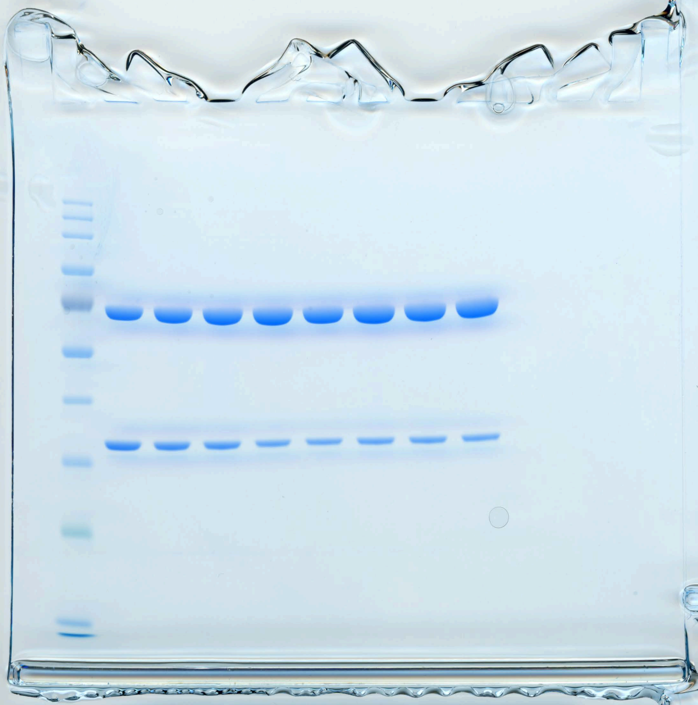

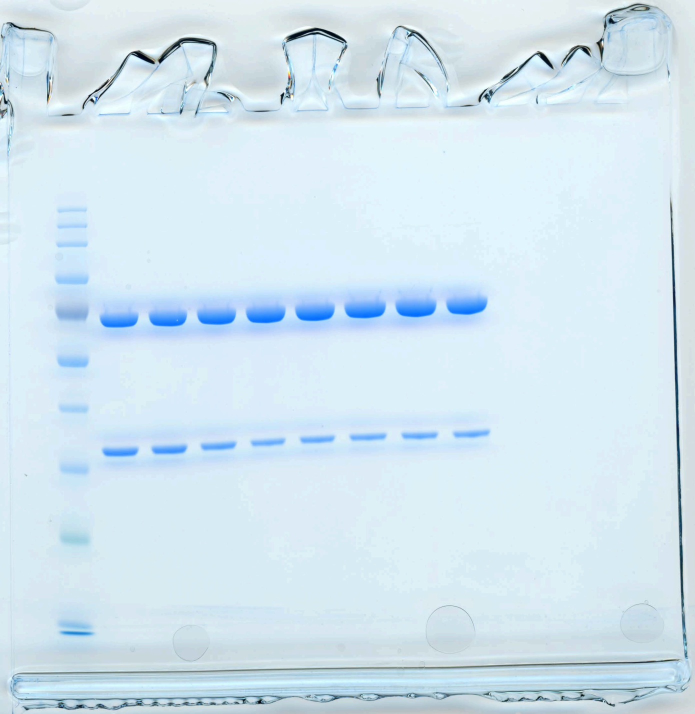

ParB (E135A)

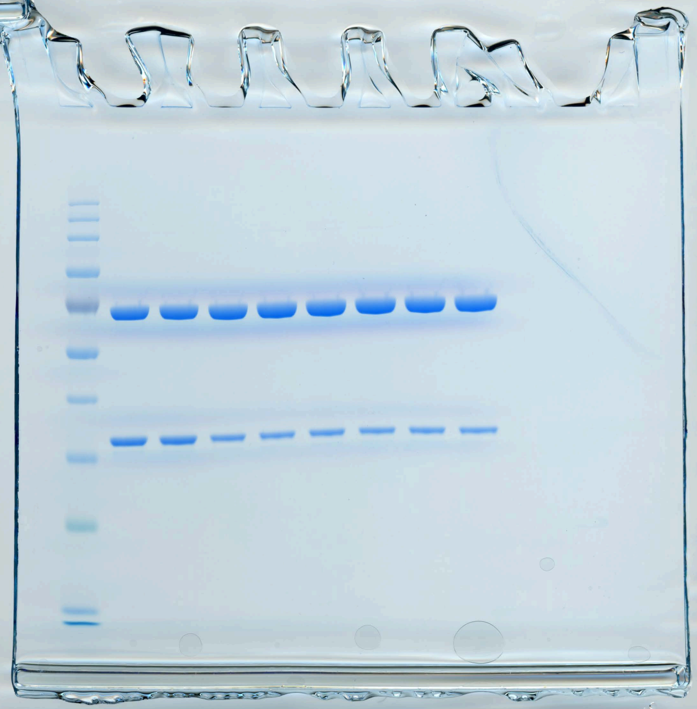

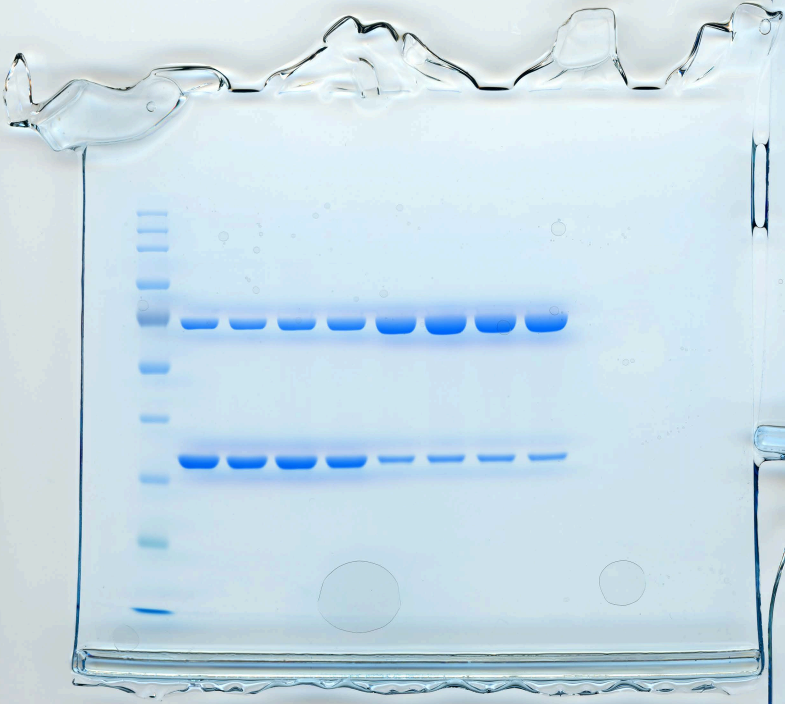

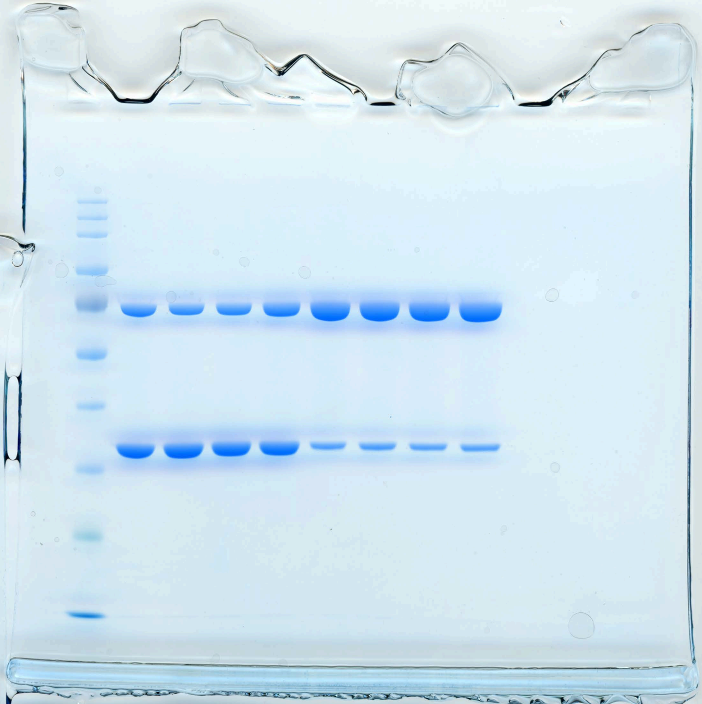

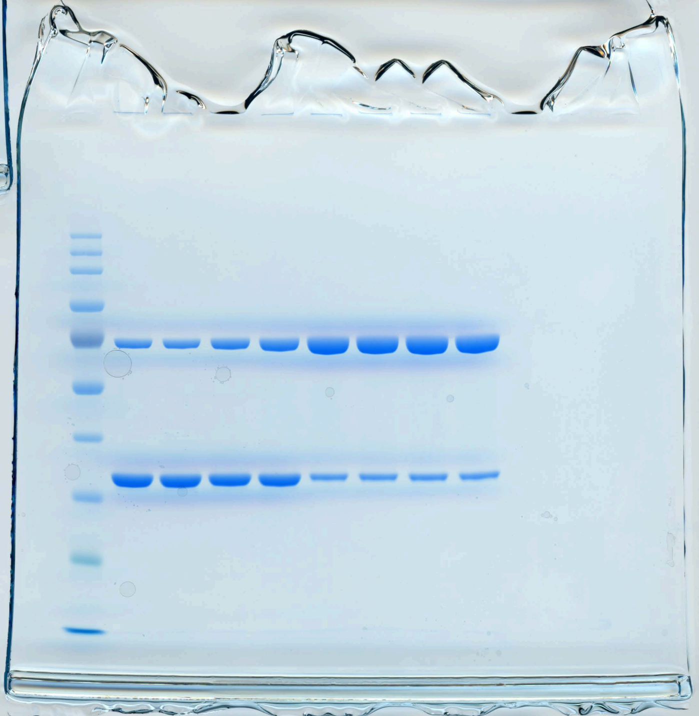

ParB(E102A)

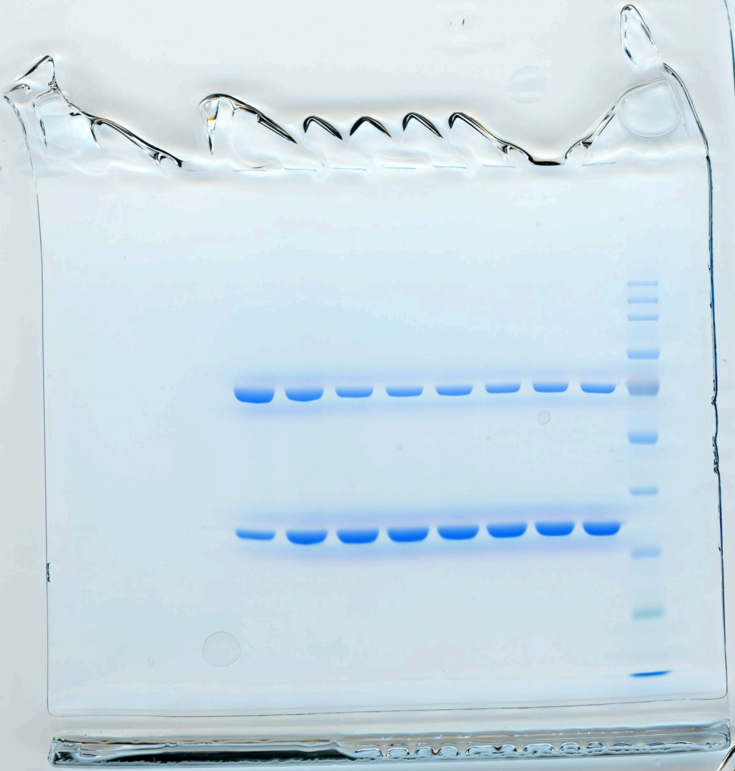
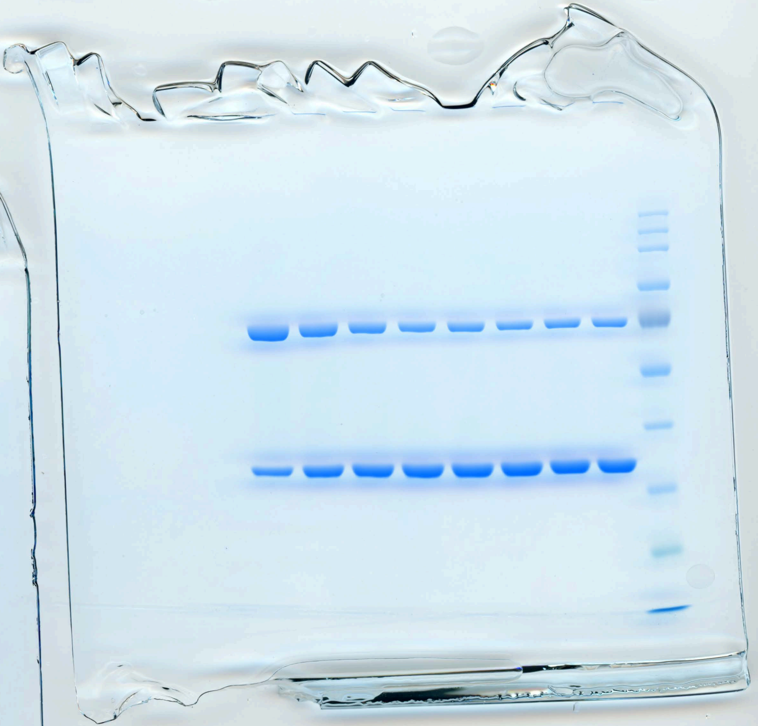
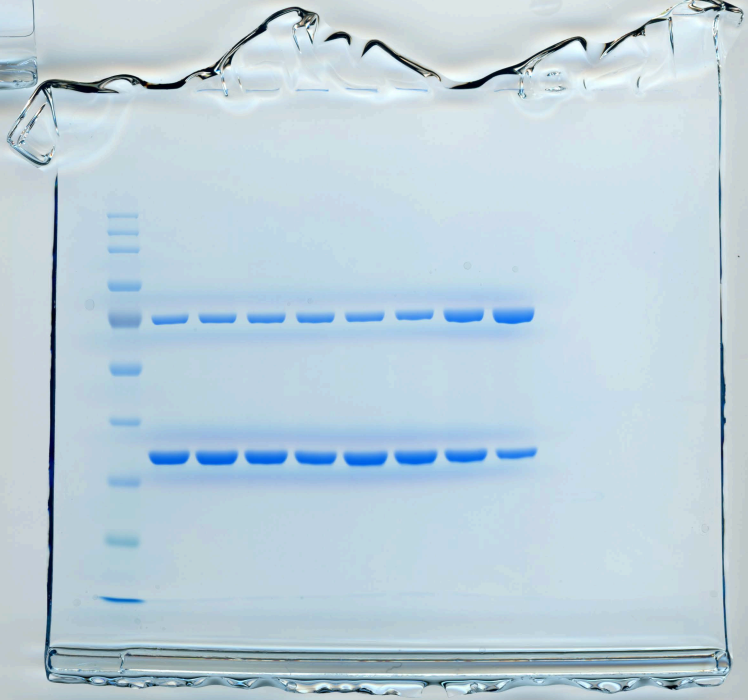

ParB(G79S)

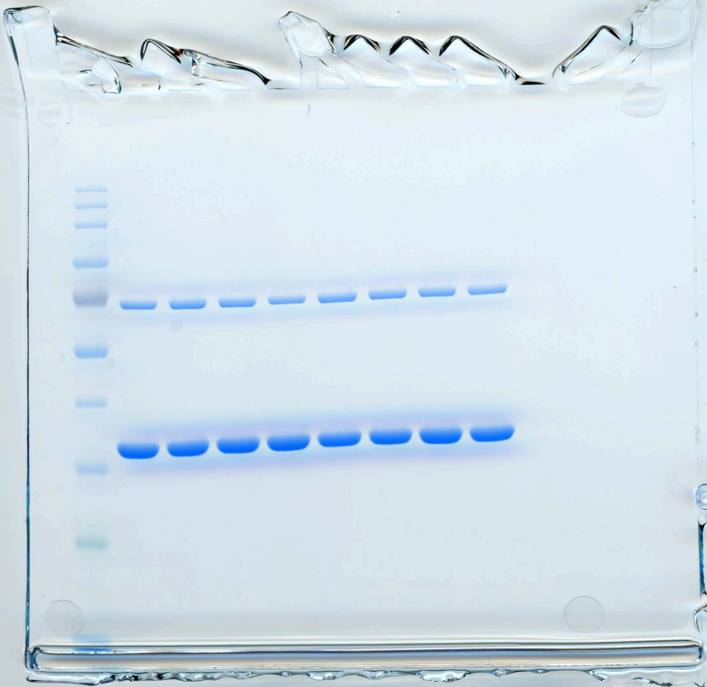
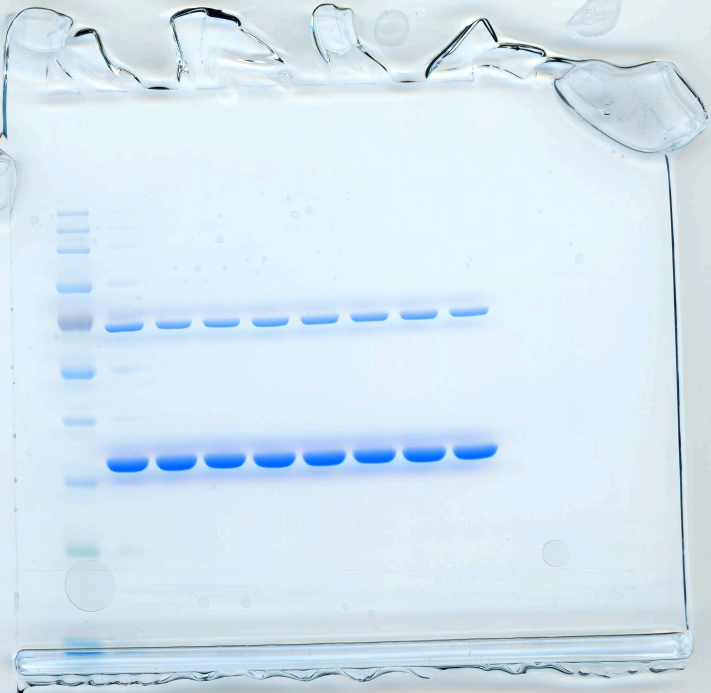
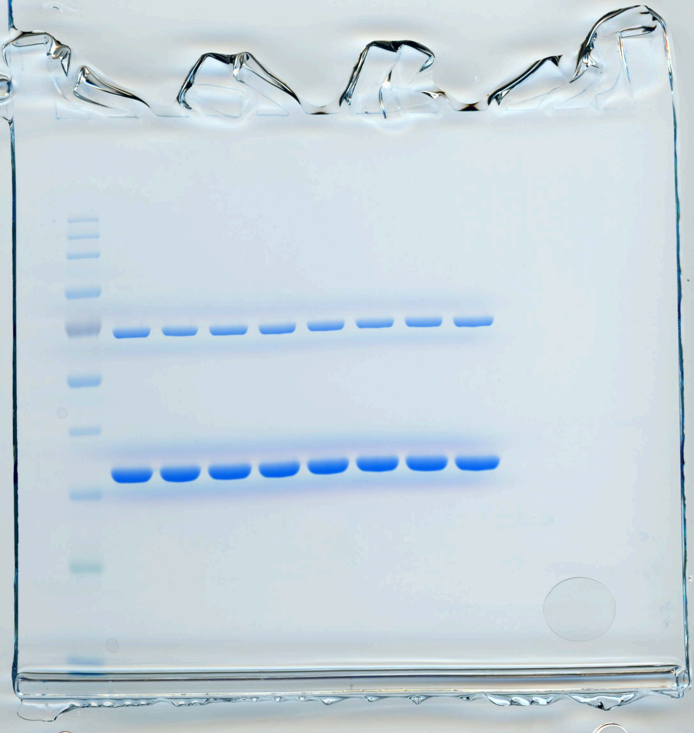

ParB (R104A)
