# Supplementary material for: A CTP-dependent gating mechanism enables ParB spreading on DNA
Source: eLife. 2021 Aug 16;10:e69676. doi: 10.7554/eLife.69676 (PMC8367383; doi:10.7554/eLife.69676)
Supplement: Supplementary file 1. [file elife-69676-supp1.docx]

**SUPPLEMENTARY FILE 1A. STRAINS**

| **Strains** | **Strains/descriptions** | **Source** |
| --- | --- | --- |
| AB1157 | *thr-1, ara-14, leuB6, Δ(gpt-proA)62, lacY1, tsx-33, supE44, galK2, rac-, hisG4(Oc), rfbD1, mgl-51, rpsL31, kdgK51, xyl-5, mtl-1, argE3 (Oc), thi-1, qsr-* | Yale *E. coli* Genetic Stock Center |
| DH5α | *E. coli* host for DNA cloning and propagation of plasmid | Le lab collection |
| Rosetta (DE3) | *E. coli* host for protein overexpression from an IPTG-inducible T7 promoter F^-^ *ompT hsdS*_B_(r_B_^-^ m_B_^-^) *gal dcm* (DE3) pRARE (chloramphenicol^R^) | Merck |
| CB15N | Wild-type synchronizable *Caulobacter crescentus* | Lab collection |
| MT148 | CB15N *parB*::P*_xyl_-parB mipZ::mipZ-yfp* | gift from Martin Thanbichler  (Thanbichler and Shapiro, 2006) |
| TLE3050 | CB15N *parB*::P*_xyl_-parB van::P_van_*-1x*flag*-*parB* (WT) | This study |
| TLE3051 | CB15N *parB*::P*_xyl_-parB van::P_van_*-1x*flag*-*parB* (S74A) | This study |
| TLE3052 | CB15N *parB*::P*_xyl_-parB van::P_van_*-1x*flag*-*parB* (Q82A) | This study |
| TLE3053 | CB15N *parB*::P*_xyl_-parB van::P_van_*-1x*flag*-*parB* (R139A) | This study |
| TLE3054 | CB15N *parB*::P*_xyl_-parB van::P_van_*-1x*flag*-*parB* (N136A) | This study |
| TLE3055 | CB15N *parB*::P*_xyl_-parB van::P_van_*-1x*flag*-*parB* (R103A) | This study |
| TLE3056 | CB15N *parB*::P*_xyl_-parB van::P_van_*-1x*flag*-*parB* (Q58A) | This study |
| TLE3057 | CB15N *parB*::P*_xyl_-parB van::P_van_*-1x*flag*-*parB* (R60A) | This study |
| TLE3058 | CB15N *parB*::P*_xyl_-parB van::P_van_*-1x*flag*-*parB* (E135A) | This study |
| TLE3059 | CB15N *parB*::P*_xyl_-parB van::P_van_*-1x*flag*-*parB* (E102A) | This study |
| TLE3060 | CB15N *parB*::P*_xyl_-parB van::P_van_*-1x*flag*-*parB* (G79S) | This study |
| TLE1146 | AB1157 *ygcE::260-bp parS*^3-4^*::apramycin^R^* | This study |
| TLE3077 | AB1157 + pKTN25-*cfp-parB* (WT) | This study |
| TLE3078 | AB1157 + pKTN25-*cfp-parB* (E102A) | This study |
| TLE3079 | TLE1146 + pKTN25-*cfp-parB* (WT) | This study |
| TLE3080 | TLE1146 + pKTN25-*cfp-parB* (E102A) | This study |
|  | BL21 Rosetta (DE3) + various pET21b-based protein overexpression vectors (see the plasmid list for the complete collection of protein overexpression plasmids) | This study |

**SUPPLEMENTARY FILE 1B. PLASMIDS, OLIGOS, AND PROTEIN SEQUENCES**

| **Plasmids/DNA** | **Description** | **Source** |
| --- | --- | --- |
| pET21b::*C. crescentus* ParB-His_6_ | Overexpression of a C-terminally His_6_-tagged *C. crescentus* ParB, carbenicillin^R^  >*C. crescentus* ParB (WT)-His_6_  MSEGRRGLGRGLSALLGEVDAAPAQAPGEQLGGSREAPIEILQRNPDQPRRTFREEDLEDLSNSIREKGVLQPILVRPSPDTAGEYQIVAGERRWRAAQRAGLKTVPIMVRELDDLAVLEIGIIENVQRADLNVLEEALSYKVLMEKFERTQENIAQTIGKSRSHVANTMRLLALPDEVQSYLVSGELTAGHARAIAAAADPVALAKQIIEGGLSVRETEALARKAPNLSAGKSKGGRPPRVKDTDTQALESDLSSVLGLDVSIDHRGSTGTLTITYATLEQLDDLCNRLTRGIKLAAALEHHHHHH* | Gift from C. Jacob-Wagner (Lim et al., 2014) |
| pET21b::*C. crescentus* ParB∆CTD-His_6_ | Overexpression of a C-terminally truncated His_6_-tagged *C. crescentus* ParB (the last 50 amino acids of ParB were removed), carbenicillin^R^ | This study |
| pET21b::*C. crescentus* ParB (Q58A)-His_6_ | Overexpression of a C-terminally His_6_-tagged *C. crescentus* ParB (Q58A), carbenicillin^R^ | This study |
| pET21b::*C. crescentus* ParB (R60A)-His_6_ | Overexpression of a C-terminally His_6_-tagged *C. crescentus* ParB (R60A), carbenicillin^R^ | This study |
| pET21b::*C. crescentus* ParB (E102A)-His_6_ | Overexpression of a C-terminally His_6_-tagged *C. crescentus* ParB (E102A), carbenicillin^R^ | This study |
| pET21b::*C. crescentus* ParB (R103A)-His_6_ | Overexpression of a C-terminally His_6_-tagged *C. crescentus* ParB (R103), carbenicillin^R^ | This study |
| pET21b::*C. crescentus* ParB (R104A)-His_6_ | Overexpression of a C-terminally His_6_-tagged *C. crescentus* ParB (R104A), carbenicillin^R^ | This study |
| pET21b::*C. crescentus* ParB (E135A)-His_6_ | Overexpression of a C-terminally His_6_-tagged *C. crescentus* ParB (E135A), carbenicillin^R^ | This study |
| pET21b::*C. crescentus* ParB (N136A)-His_6_ | Overexpression of a C-terminally His_6_-tagged *C. crescentus* ParB (N136A), carbenicillin^R^ | This study |
| pET21b::*C. crescentus* ParB (R139A)-His_6_ | Overexpression of a C-terminally His_6_-tagged *C. crescentus* ParB (R139A), carbenicillin^R^ | This study |
| pET21b::*C. crescentus* ParB (S74A)-His_6_ | Overexpression of a C-terminally His_6_-tagged *C. crescentus* ParB (S74A), carbenicillin^R^ | This study |
| pET21b::*C. crescentus* ParB (G79S)-His_6_ | Overexpression of a C-terminally His_6_-tagged *C. crescentus* ParB (G79S), carbenicillin^R^ | This study |
| pET21b::*C. crescentus* ParB (Q82A)-His_6_ | Overexpression of a C-terminally His_6_-tagged *C. crescentus* ParB (Q82A), carbenicillin^R^ | This study |
| pET21b::*C. crescentus* ParB (Q35C C297S)-His_6_ | Overexpression of a C-terminally His_6_-tagged *C. crescentus* ParB (Q35C C297S), carbenicillin^R^ | (Jalal et al., 2020) |
| pET21b::*C. crescentus* ParB (L224C C297S)-His_6_ | Overexpression of a C-terminally His_6_-tagged *C. crescentus* ParB (Q35C C297S), carbenicillin^R^ | This study |
| pET21b::*C. crescentus* ParB (I304C C297S)-His_6_ | Overexpression of a C-terminally His_6_-tagged *Caulobacter* ParB (Q35C C297S), carbenicillin^R^ | This study |
| pET21b::*C. crescentus* ParB (Q58A Q35C C297S)-His_6_ | Overexpression of a C-terminally His_6_-tagged *C. crescentus* ParB (Q58A Q35C C297S), carbenicillin^R^ | This study |
| pET21b::*C. crescentus* ParB (R60A Q35C C297S)-His_6_ | Overexpression of a C-terminally His_6_-tagged *C. crescentus* ParB (R60A Q35C C297S), carbenicillin^R^ | This study |
| pET21b::*C. crescentus* ParB (E102A Q35C C297S)-His_6_ | Overexpression of a C-terminally His_6_-tagged *C. crescentus* ParB (E102A Q35C C297S), carbenicillin^R^ | This study |
| pET21b::*C. crescentus* ParB (R103A Q35C C297S)-His_6_ | Overexpression of a C-terminally His_6_-tagged *C. crescentus* ParB (R103 Q35C C297S), carbenicillin^R^ | This study |
| pET21b::*C. crescentus* ParB (R104A Q35C C297S)-His_6_ | Overexpression of a C-terminally His_6_-tagged *C. crescentus* ParB (R104A Q35C C297S), carbenicillin^R^ | This study |
| pET21b::*C. crescentus* ParB (E135A Q35C C297S)-His_6_ | Overexpression of a C-terminally His_6_-tagged *C. crescentus* ParB (E135A Q35C C297S), carbenicillin^R^ | This study |
| pET21b::*C. crescentus* ParB (N136A Q35C C297S)-His_6_ | Overexpression of a C-terminally His_6_-tagged *C. crescentus* ParB (N136A Q35C C297S), carbenicillin^R^ | This study |
| pET21b::*C. crescentus* ParB (R139A Q35C C297S)-His_6_ | Overexpression of a C-terminally His_6_-tagged *C. crescentus* ParB (R139A Q35C C297S), carbenicillin^R^ | This study |
| pET21b::*C. crescentus* ParB (S74A Q35C C297S)-His_6_ | Overexpression of a C-terminally His_6_-tagged *C. crescentus* ParB (S74A Q35C C297S), carbenicillin^R^ | This study |
| pET21b::*C. crescentus* ParB (G79S Q35C C297S)-His_6_ | Overexpression of a C-terminally His_6_-tagged *C. crescentus* ParB (G79S Q35C C297S), carbenicillin^R^ | This study |
| pET21b::*C. crescentus* ParB (Q82A Q35C C297S)-His_6_ | Overexpression of a C-terminally His_6_-tagged *C. crescentus* ParB (Q82A Q35C C297S), carbenicillin^R^ | This study |
| pET21b::*C. crescentus* ParB (Q35C I304C C297S)-His_6_ | Overexpression of C-terminally His_6_-tagged *C. crescentus* ParB (Q35C I304C C297S), carbenicillin^R^ | This study |
| pET21b::*C. crescentus* ParB (L224C I304C C297S)-His_6_ | Overexpression of C-terminally His_6_-tagged *C. crescentus* ParB ( L224C I304C C297S), carbenicillin^R^ | This study |
| pET21b::*C. crescentus* ParB (Q35C I304C C297S)-His_6_ | Overexpression of C-terminally His_6_-tagged *C. crescentus* ParB (Q35C I304C C297S), carbenicillin^R^ | This study |
| pET21b::*C. crescentus* ParB (L224C I304C C297S)-TEV-His_6_ | Overexpression of a C-terminally His_6_-tagged ParB (L224C I304C C297S)-TEV (a TEV cleavage site was engineered in between the C-terminal domain and the DBD-CTD linker of ParB), carbenicillin^R^  >*C. crescentus* ParB (L224C I304C C297S)-TEV  MSEGRRGLGRGLSALLGEVDAAPAQAPGEQLGGSREAPIEILQRNPDQPRRTFREEDLEDLSNSIREKGVLQPILVRPSPDTAGEYQIVAGERRWRAAQRAGLKTVPIMVRELDDLAVLEIGIIENVQRADLNVLEEALSYKVLMEKFERTQENIAQTIGKSRSHVANTMRLLALPDEVQSYLVSGELTAGHARAIAAAADPVALAKQIIEGGCSVRETEALARKAPNLSAGKSKGGRPPRVKD**ENLYFQSGGGS**TDTQALESDLSSVLGLDVSIDHRGSTGTLTITYATLEQLDDLSNRLTRGCKL AAALEHHHHHH*  (**TEV cleavage site in bold)** | This study |
| pUC19::260bp-*parS* | pUC19 plasmid with 260-bp insert that contains *parS* sites, carbenicillin^R^  >260-bp_*Caulobacter*_*parS*_fragment_cloned_into_pUC19  caagacgctcgcctcaatgcgaacgcccccgggttcgagcgggggcg  ctggactcgatctatacgccaatcaggcgagcgggtcgatgtgactcatc  ggcgtttcacgtgaaacacccccaccgcagctgtgagcggcctgtggac  aatattggggatgttccacgtgaaacatcacttgccgatacagaaggtcg  aaaagacccgtccaagaacgtcctcaggatcgatacggccggagatg  cgctccagggcccgggc | (Jalal et al., 2020) |
| pUC19::260bp-scrambled *parS* | pUC19 plasmid with 260-bp insert that contains scrambled *parS* sites, carbenicillin^R^  >260-bp_scrambled_*Caulobacter*_*parS*_fragment_cloned_into_pUC19  caagacgctcgcctcaatgcgaacgcccccgggttcgagcgggggcg  ctggactcgatctatacgccaatcaggcgagcgggtcgatgtgactcatc  ggacagctcgagattcatcccccaccgcagctgtgagcggcctgtggac  aatattggggaatcgagtatacgctactcacttgccgatacagaaggtcg  aaaagacccgtccaagaacgtcctcaggatcgatacggccggagatg  cgctccagggcccgggc | (Jalal et al., 2020) |
| pENTR::*attL1-parB* (WT/mutant)-*attL2* | pENTR-based plasmid with phage attachment sites *attL1 attL2* flanking the coding sequence of ParB (WT/mutants), Gateway-cloning compatible, kanamycin^R^ | Lab stock |
| pML477 | Gateway-cloning destination vector for fusion of protein interest to an N-terminally FLAG tag, xylose-inducible promoter, medium-copy number  plasmid, spectinomycin^R^ | Gift from M. Laub |
| pMT571-1xFLAG-DEST | Gateway-cloning compatible, destination vector, tetracycline^R^ | This study |
| pMT571-1xFLAG-*parB* (WT/mutants) | Expressing a FLAG-tagged version of *C. crescentus* ParB (WT/mutants), integrative at the *vanA* locus, tetracycline^R^ | This study |
| pKTN25 | Vector for bacterial two-hybrid assay, medium-copy number, kanamycin^R^ | (Karimova et al., 1998) |
| pKTN25-*cfp-parB* (WT) | Expressing a CFP-tagged version of *C. crescentus* ParB (WT), P*_lac_*, medium-copy number plasmid, kanamycin^R^ | This study |
| pKTN25-*cfp-parB* (E102A) | Expressing a CFP-tagged version of *C. crescentus* ParB (E102A), P*_lac_*, medium-copy number plasmid, kanamycin^R^ | This study |
| 170bp_*parS* | cgccagggttttcccagtcacgacgttgtaaaacgacggccagtgaattcgagctcggtac  ccgcaggaggacgtagggtaggggga**tgtttcacgtgaaaca**ggggatcctctagagtc  gacctgcaggcatgcaagcttggcgtaatcatggtcatagctgtttcct  (***parS* site in bold**) | (Jalal et al., 2020) |
| FLAG-*attR1-ccdB*-chloramphenicol^R^-*attR2* cassette | gtggactacaaggacgacgacgacaagggctcggtcgaatca**acaagtttgtacaaaaaagctgaacgagaaacgtaaaatgatataaatatcaatatattaaattagattttgcataaaaaacagactacataatactgtaaaacacaacatatccagtcactatg**gcggccgcattaggcaccccaggctttacactttatgcttccggctcgtataatgtgtggattttgagttaggatccggcgagattttcaggagctaaggaagctaaaatggagaaaaaaatcactggatataccaccgttgatatatcccaatggcatcgtaaagaacattttgaggcatttcagtcagttgctcaatgtacctataaccagaccgttcagctggatattacggcctttttaaagaccgtaaagaaaaataagcacaagttttatccggcctttattcacattcttgcccgcctgatgaatgctcatccggaattccgtatggcaatgaaagacggtgagctggtgatatgggatagtgttcacccttgttacaccgttttccatgagcaaactgaaacgttttcatcgctctggagtgaataccacgacgatttccggcagtttctacacatatattcgcaagatgtggcgtgttacggtgaaaacctggcctatttccctaaagggtttattgagaatatgtttttcgtctcagccaatccctgggtgagtttcaccagttttgatttaaacgtggccaatatggacaacttcttcgcccccgttttcaccatgggcaaatattatacgcaaggcgacaaggtgctgatgccgctggcgattcaggttcatcatgccgtctgtgatggcttccatgtcggcagaatgcttaatgaattacaacagtactgcgatgagtggcagggcggggcgtaaagatctggatccggcttactaaaagccagataacagtatgcgtatttgcgcgctgatttttgcggtataagaatatatactgatatgtatacccgaagtatgtcaaaaagaggtgtgctatgaagcagcgtattacagtgacagttgacagcgacagctatcagttgctcaaggcatatatgatgtcaatatctccggtctggtaagcacaaccatgcagaatgaagcccgtcgtctgcgtgccgaacgctggaaagcggaaaatcaggaagggatggctgaggtcgcccggtttattgaaatgaacggctcttttgctgacgagaacagggactggtgaaatgcagtttaaggtttacacctataaaagagagagccgttatcgtctgtttgtggatgtacagagtgatattattgacacgcccgggcgacggatggtgatccccctggccagtgcacgtctgctgtcagataaagtctcccgtgaactttacccggtggtgcatatcggggatgaaagctggcgcatgatgaccaccgatatggccagtgtgccggtctccgttatcggggaagaagtggctgatctcagccaccgcgaaaatgacatcaaaaacgccattaacctgatgttctggggaatataaatgtcaggctcccttatacacagccagtctgcaggtcgac**catagtgactggatatgttgtgttttacagtattatgtagtctgttttttatgcaaaatctaatttaatatattgatatttatatcattttacgtttctcgttcagctttcttgtacaaagtggt**  **(1x*flag* is underlined, *attR1* and *attR2* in bold)** | This study |
| 260bp-*parS*^3-4^ -aac(3)IV apramycin^R^ cassette | ggggaatgtggattcgatccagagctggtcgaatgcgtaa**gcccgggccctggagcgcatctccggccgtatcgatcctgaggacgttcttggacgggtcttttcgaccttctgtatcggcaagtgatgtttcacgtggaacatccccaatattgtccacaggccgctcacagctgcggtgggggtgtttcacgtgaaacgccgatgagtcacatcgacccgctcgcctgattggcgtatagatcgagtccagcgcccccgctcgaacccgggggcgttcgcattgaggcgagcg**tcttgatccttttggttcatgtgcagctccatcagcaaaaggggatgataagtttatcaccaccgactatttgcaacagtgccgttgatcgtgctatgatcgactgatgtcatcagcggtggagtgcaatgtcgtgcaatacgaatggcgaaaagccgagctcatcggtcagcttctcaaccttggggttacccccggcggtgtgctgctggtccacagctccttccgtagcgtccggcccctcgaagatgggccacttggactgatcgaggccctgcgtgctgcgctgggtccgggagggacgctcgtcatgccctcgtggtcaggtctggacgacgagccgttcgatcctgccacgtcgcccgttacaccggaccttggagttgtctctgacacattctggcgcctgccaaatgtaaagcgcagcgcccatccatttgcctttgcggcagcggggccacaggcagagcagatcatctctgatccattgcccctgccacctcactcgcctgcaagcccggtcgcccgtgtccatgaactcgatgggcaggtacttctcctcggcgtgggacacgatgccaacacgacgctgcatcttgccgagttgatggcaaaggttccctatggggtgccgagacactgcaccattcttcaggatggcaagttggtacgcgtcgattatctcgagaatgaccactgctgtgagcgctttgccttggcggacaggtggctcaaggagaagagccttcagaaggaaggtccagtcggtcatgcctttgctcggttgatccgctcccgcgacattgtggcgacagccctgggtcaactgggccgagatccgttgatcttcctgcatccgccagaggcgggatgcgaagaatgcgatgccgctcgccagtcgattggctgagtgaggaaggccgggcgggaaactgcccggcctgaacata  (**260-bp *parS*^3-4^ in bold**) | This study |
| **Oligos** | **Description** | **Source** |
| 22-bp *parS* | ggatgtttcacgtgaaacatcc | (Jalal et al., 2020) |
| NdeI-Ct-ParB-F | taactttaagaaggagatatacatatgtccgaagggcgtcgtggtctgggtc | This study |
| HindIII-Ct-ParB-R | ggtggtgctcgagtgcggccgcaagcttgtccttcacgcgtggggggcggcc | This study |
| P1304 | ggggaatgtggattcgatccagagctggtcgaatgcgtaagcccgggccctggagcgcatctc | This study |
| P1305 | agctgcacatgaaccaaaaggatcaagacgctcgcctcaatgcgaacgc | This study |
| P1306 | atccttttggttcatgtgcagctcc | This study |
| P1307 | tatgttcaggccgggcagtttcccgcccggccttcctcactcagccaatcgactggcgagcg | This study |
| P1952 | ccacgatgcgaggaaacgcatatggactacaaggacgacgacgacaagggctcgg | This study |
| P1953 | actagtggatcccccgggctgcagaccactttgtacaagaaagctgaacgagaaacg | This study |
| P3392 | taattaatatgcatggtaccttaagatctagcgaaggtcgtcgtggcttgggac | This study |
| P3393 | cagctagcaccggtacgcgtaacgttcgttaaattccacgggtaaggcggttac | This study |
| P3396 | cagctatgaccatgattacgccaagcttggtgagcaagggcgaggagctgttcac | This study |
| P3397 | tgcaccatattacttagttatatcgatttaaattccacgggtaaggcggttacac | This study |

**SUPPLEMENTARY FILE 1C. ChIP-SEQ DATASET**

| **ChIP-seq datasets** | **GEO** |
| --- | --- |
| CB15N *parB::*P*_xyl_-parB van::*P*_van_-1xflag-parB* (WT), fixation with 1% formaldehyde, α-FLAG antibody (Sigma), ChIP fraction, replicate 1 | GSE168968, this study |
| CB15N *parB::*P*_xyl_-parB van::*P*_van_-1xflag-parB* (WT), fixation with 1% formaldehyde, α-FLAG antibody (Sigma), ChIP fraction, replicate 2 | GSE168968, this study |
| CB15N *parB::*P*_xyl_-parB van::*P*_van_-1xflag-parB* (E102A), fixation with 1% formaldehyde, α-FLAG antibody (Sigma), ChIP fraction, replicate 1 | GSE168968, this study |
| CB15N *parB::*P*_xyl_-parB van::*P*_van_-1xflag-parB* (E102A), fixation with 1% formaldehyde, α-FLAG antibody (Sigma), ChIP fraction, replicate 2 | GSE168968, this study |
| TLE3079 AB1157 *ygcE::260bp parS*^3-4^*::apramycin^R^ _­_*+ pKTN25-*cfp-parB* (WT), fixation with 1% formaldehyde, α-GFP antibody (Abcam), ChIP fraction, replicate 1 | GSE168968, this study |
| TLE3079 AB1157 *ygcE::260bp parS*^3-4^*::apramycin^R^ _­_*+ pKTN25-*cfp-parB* (WT), fixation with 1% formaldehyde, α-GFP antibody (Abcam), ChIP fraction, replicate 2 | GSE168968, this study |
| TLE3080 AB1157 *ygcE::260bp parS*^3-4^*::apramycin^R^ _­_*+ pKTN25-*cfp-parB* (E102A), fixation with 1% formaldehyde, α-GFP antibody (Abcam), ChIP fraction, replicate 1 | GSE168968, this study |
| TLE3080 AB1157 *ygcE::260bp parS*^3-4^*::apramycin^R^ _­_*+ pKTN25-*cfp-parB* (E102A), fixation with 1% formaldehyde, α-GFP antibody (Abcam), ChIP fraction, replicate 2 | GSE168968, this study |
