# Supplementary material for: A CTP-dependent gating mechanism enables ParB spreading on DNA
Source: eLife. 2021 Aug 16;10:e69676. doi: 10.7554/eLife.69676 (PMC8367383; doi:10.7554/eLife.69676)
Supplement: Transparent reporting form [file elife-69676-transrepform1.docx]

***eLife’s* transparent reporting form**

We encourage authors to provide detailed information *within their submission* to facilitate the interpretation and replication of experiments. Authors can upload supporting documentation to indicate the use of appropriate reporting guidelines for health-related research (see [EQUATOR Network](http://www.equator-network.org/%20)), life science research (see the [BioSharing Information Resource](https://biosharing.org/)), or the [ARRIVE guidelines](http://www.plosbiology.org/article/info:doi/10.1371/journal.pbio.1000412) for reporting work involving animal research. Where applicable, authors should refer to any relevant reporting standards documents in this form.

If you have any questions, please consult our Journal Policies and/or contact us: [editorial@elifesciences.org](mailto:editorial@elifesciences.org).

**Sample-size estimation**

- You should state whether an appropriate sample size was computed when the study was being designed
- You should state the statistical method of sample size computation and any required assumptions
- If no explicit power analysis was used, you should describe how you decided what sample (replicate) size (number) to use

Please outline where this information can be found within the submission (e.g., sections or figure legends), or explain why this information doesn’t apply to your submission:

*-No power analysis were employed to prepare statistical samples. For assays used in this study, technical and biological replicates are the primary concern.*

**Replicates**

- You should report how often each experiment was performed
- You should include a definition of biological versus technical replication
- The data obtained should be provided and sufficient information should be provided to indicate the number of independent biological and/or technical replicates
- If you encountered any outliers, you should describe how these were handled
- Criteria for exclusion/inclusion of data should be clearly stated
- High-throughput sequence data should be uploaded before submission, with a private link for reviewers provided (these are available from both GEO and ArrayExpress)

Please outline where this information can be found within the submission (e.g., sections or figure legends), or explain why this information doesn’t apply to your submission:

*-The number of replicates was reported in the figure legends and in the Materials and Methods. Each experiment was performed at least three times.*

*-The definition of biological and technical replicates can be found in the Materials and Methods section (Page 20, lines 596-598 of the current manuscript).*

*-The exclusion of outliers was not required for this study.*

**Statistical reporting**

- Statistical analysis methods should be described and justified
- Raw data should be presented in figures whenever informative to do so (typically when N per group is less than 10)
- For each experiment, you should identify the statistical tests used, exact values of N, definitions of center, methods of multiple test correction, and dispersion and precision measures (e.g., mean, median, SD, SEM, confidence intervals; and, for the major substantive results, a measure of effect size (e.g., Pearson's r, Cohen's d)
- Report exact p-values wherever possible alongside the summary statistics and 95% confidence intervals. These should be reported for all key questions and not only when the p-value is less than 0.05.

Please outline where this information can be found within the submission (e.g., sections or figure legends), or explain why this information doesn’t apply to your submission:

*-Precision and dispersion measures (standard deviation) were used in this study. The details can be found in the Materials and Methods section, and in the figure legends.*

*-The name and functions of software used in this study were reported in the Materials and Methods section.*

(For large datasets, or papers with a very large number of statistical tests, you may upload a single table file with tests, Ns, etc., with reference to sections in the manuscript.)

**Group allocation**

- Indicate how samples were allocated into experimental groups (in the case of clinical studies, please specify allocation to treatment method); if randomization was used, please also state if restricted randomization was applied
- Indicate if masking was used during group allocation, data collection and/or data analysis

Please outline where this information can be found within the submission (e.g., sections or figure legends), or explain why this information doesn’t apply to your submission:

*-No special method for group allocation was used.*

*-For each replicate, experimental and control groups were imaged, processed, and analyzed together. A detailed description of the processing and analysis was reported in the Materials and Methods section.*

**Additional data files (“source data”)**

- We encourage you to upload relevant additional data files, such as numerical data that are represented as a graph in a figure, or as a summary table
- Where provided, these should be in the most useful format, and they can be uploaded as “Source data” files linked to a main figure or table
- Include model definition files including the full list of parameters used
- Include code used for data analysis (e.g., R, MatLab)
- Avoid stating that data files are “available upon request”

Please indicate the figures or tables for which source data files have been provided:

*The accession number for the sequencing data reported in this paper is GSE168968. Atomic coordinates for protein crystal structures reported in this paper were deposited in the RCSB Protein Data Bank with the accession number 6T1F and 7BM8.*

*All of these data are already open to the public. Validation reports for PDB files are also uploaded to the accompany this submission.*

*For figures containing gels or blots, raw unedited images (and an accompanying annotated figures) have been uploaded as source data (Fig. 5, Fig.6, Fig.8, Fig5-fig supplement1, 2, and 3, Fig6-fig supplement1 and 2, and Fig8-fig supplement1 and 2).*
